# Dipolar Microenvironment Engineering Enabled by Electron Beam Irradiation for Boosting Catalytic Performance

**DOI:** 10.1002/advs.202401562

**Published:** 2024-06-11

**Authors:** Zhiyan Chen, Shuai Hao, Haozhe Li, Xiaohan Dong, Xihao Chen, Jushigang Yuan, Alexander Sidorenko, Jiang Huang, Yanlong Gu

**Affiliations:** ^1^ Huazhong University of Science and Technology 1037 Luoyu Road Hongshan District Wuhan 430074 China; ^2^ Key Laboratory of Material Chemistry for Energy Conversion and Storage Ministry of Education Hubei Key Laboratory of Material Chemistry and Service Failure Huazhong University of Science and Technology Wuhan 430074 China; ^3^ State Key Laboratory of Advanced Electromagnetic Engineering and Technology Huazhong University of Science and Technology Wuhan 430074 China; ^4^ Institute of Chemistry of New Materials of National Academy of Sciences of Belarus Minsk 220084 Belarus

**Keywords:** biomass‐derived platform molecules, dipolar catalyst, electron beam irradiation, microenvironment

## Abstract

Creating a diverse dipolar microenvironment around the active site is of great significance for the targeted induction of intermediate behaviors to achieve complicated chemical transformations. Herein, an efficient and general strategy is reported to construct hypercross‐linked polymers (HCPs) equipped with tunable dipolar microenvironments by knitting arene monomers together with dipolar functional groups into porous network skeletons. Benefiting from the electron beam irradiation modification technique, the catalytic sites are anchored in an efficient way in the vicinity of the microenvironment, which effectively facilitates the processing of the reactants delivered to the catalytic sites. By varying the composition of the microenvironment scaffold structure, the contact and interaction behavior with the reaction participants can be tuned, thereby affecting the catalytic activity and selectivity. As a result, the framework catalysts produced in this way exhibit excellent catalytic performance in the synthesis of glycinate esters and indole derivatives. This manipulation is reminiscent of enzymatic catalysis, which adjusts the internal polarity environment and controls the output of products by altering the scaffold structure.

## Graphical abstract

Creating diverse dipolar microenvironments on the catalyst surface not only effectively promotes the synthesis of indole from aniline and α‐hydroxyacetophenone and the conversion of glyoxal into ethyl phenylglycinate derivatives, but also reduces dependence on unfriendly solvents.

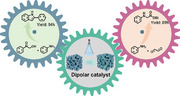

## 1 Introduction

In nature, enzymes serve as advanced catalysts that efficiently catalyze chemical transformations by providing favorable intermolecular non‐covalent interactions in well‐designed spaces where catalytic sites are surrounded by amino acid residues.^[^ ^1^, ^2^ ^]^ Inspired by the enzymes, a promising strategy that has emerged in heterogeneous catalysis is to create suitable reaction microenvironments around active sites to enhance the performance of catalysts through specific interactions.^[^ ^3^, ^4^, ^5^ ^]^ The use of these non‐covalent interactions for accurately manipulating molecular behaviors is not only viable but also highly effective in achieving complicated catalytic transformations.^[^ ^6^, ^7^, ^8^ ^]^ Practically, dipolar modification on the catalyst surface through the introduction of functional groups might confer potential opportunities to facilely modulate or alter the intermolecular forces.^[^ ^9^, ^10^, ^11^ ^]^ Optimizing these interactions facilitates the formation of desired intermediates, which is highly beneficial to achieving the optimal balance of high activity and selectivity. Despite the fact that the catalysts with dipolar microenvironments have been proven to have the ability to fulfill the goal of catalytic applications in an environmentally friendly manner, their restricted functionality and monotonous structure have resulted in limited applicability.^[^ ^12^, ^13^, ^14^ ^]^

Hypercrosslinked polymers (HCPs) are a class of porous materials featuring low cost, high stability, and ease of functionalization.^[^ ^15^, ^16^, ^17^, ^18^ ^]^ Because a variety of arene units can be easily knitted into a network structure, which in turn serves as a platform to manipulate molecular behaviors by providing adequate contacts with the substrates, HCPs should be appropriate options to flexibly fabricate a diversified microenvironment. Although profitable, effective chemical modification is cumbersome and time‐consuming. Hence, it is imperative to introduce a novel technology to enhance the efficiency of surface modification.

On the other hand, electron beam irradiation (EBI) is considered a clean, environmentally friendly technology that can precisely control the modification process and achieve targeted surface treatment.^[^ ^19^, ^20^, ^21^ ^]^ The EBI process involves the direct injection of high‐energy electron beams into the material, triggering chemical reactions and structural changes, thereby improving the performance and quality of the material through precise control of irradiation parameters.^[^ ^22^ ^]^ This technology not only greatly enhances production efficiency and energy conservation but also prevents the generation of chemical waste and hazardous byproducts by eliminating the need for catalysts or additives. The EBI technology provides us with a remarkable opportunity for preparing dipolar catalysts with tunable microenvironments that are unattainable with traditional methods.

In this work, we report a general and feasible strategy for the construction of dipolar catalysts with tunable polarity by EBI technology. A series of HCPs‐catalysts with diverse dipolar regions have been fabricated to study the effect of the surface interactions and their resultant influence on the formation of the reaction products. The catalysts can be used in the synthesis of indoles from anilines and α‐hydroxyacetophenones, as well as in the conversion of glyoxal into ethyl phenylglycinate derivatives via Henys rearrangement. The unique microenvironment with multifunctional dipolar species at the surface can significantly enhance catalytic performance, allowing us to maximize the efficiency of the targeted reactions. Our finding not only brings innovation to the design of catalysts, but also provides a cost‐effective approach to addressing the challenge of the trade‐off effect between catalytic activity and selectivity.

## 2 Results and Discussion

### 2.1 Preparation and Characterization of Dipolar Catalysts

**Scheme 1:**
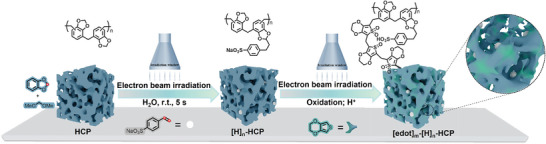
Schematic illustration of the preparation process of [edot]_m_‐[H]_n_‐HCP catalysts.

The dipolar catalysts with tunable properties were prepared through the combination of traditional knitting strategies and the EBI process, as illustrated in **Scheme** **1**. In typical synthetic procedures, 1,3‐benzodioxole was used as an arena unit along with an electrophilic cross‐linker, dimethoxymethane, to fabricate the precursor of the HCP framework (Figures S1–S3, Supporting Information). The methylenedioxy moiety in 1,3‐benzodioxole confers significant reactivity, which can serve hopefully as a reactive site to anchor the catalytically active functional group through radical reactions. Unfortunately, owing to the poor reactivity, this grafting reaction has been rarely used under conventional conditions.^[^ ^23^, ^24^, ^25^ ^]^ In this work, sodium *p*‐styrenesulfonate as a sulfonate‐inducing reagent was efficiently installed on the primary architecture of HCP with the assistance of the EBI technique via rapid free radical reaction.^[^ ^26^, ^27^ ^]^ This was a key for catalysts to ensure good catalytic performance. The EBI treatment was implemented at room temperature for 5 s in H_2_O. Then, the obtained material was treated again under knitting conditions by using 3,4‐ethylenedioxythiophene as a two‐site structural monomer in conjunction with using dimethoxymethane as the electrophilic linker. The thereby obtained material was then subjected to a facile oxidation and acidification treatment.

These procedures allowed us to weave the pore walls around the active sites with dipolar functional groups, providing adequate access to the substrate. Benefiting from this, these tailored functional groups, which can serve as “dipole modulators”, were capable of influencing the catalytic reaction process through non‐covalent interactions with the reactants. This parallels the method by which homogeneous catalysts can be modulated through non‐covalent interactions.^[^ ^28^ ^]^ Therefore, the catalytic active units along with the dipolar groups embedded in a specific microenvironment allow modifications to the framework to be correlated with catalytic metrics. The obtained material was denoted as [edot]_m_‐[H]_n_‐HCP, where *m* and *n* represent the molar percentage of functional groups in the entire groups, and the grafting content of the catalytic center, respectively.

**Figure 1:**
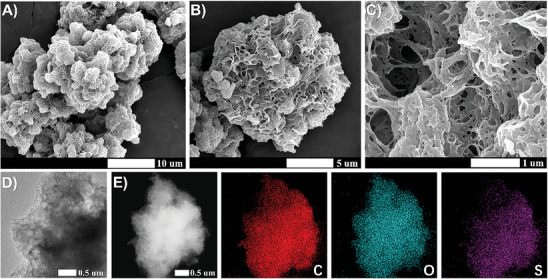
A–C) Representative SEM images of [edot]_0.34_‐[H]_0.60_‐HCP samples, scale bars denote 10, 5, and 1 µm, respectively. D) TEM image and E) aberration‐corrected HAADF‐STEM image corresponding elemental distribution mappings for the selected area of [edot]_0.34_‐[H]_0.60_‐HCP.

**Figure 2:**
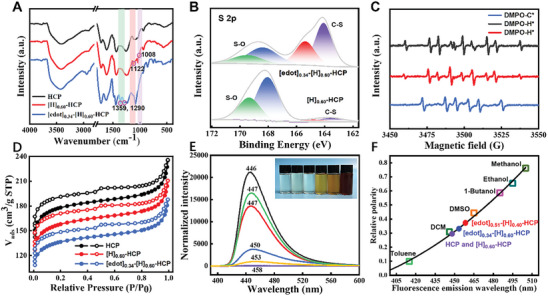
A) The FT‐IR spectra of three representative samples. B) the variation of XPS spectra for S 2p in [H]_0.60_‐HCP and [edot]_0.34_‐[H]_0.60_‐HCP. C) EPR spectra for detecting different types of free radicals with 5,5‐dimethyl‐1‐pyrroline *N*‐oxide (DMPO), in situ test results for the mixture were depicted by green and blue lines, and the red line represents the test results in pure water. D) N_2_ sorption isotherms. E) Normalized emission spectra of Prodan in dichloromethane solution or supernatants with different additives. The lines from top to bottom represent pure Prodan in dichloromethane and the addition of HCP, [H]_0.60_‐HCP, [edot]_0.34_‐[H]_0.60_‐HCP^a^, [edot]_0.34_‐[H]_0.60_‐HCP, and [edot]_0.51_‐[H]_0.40_‐HCP, where the superscript represents the unoxidized sample. The inset shows the color change of representative HCP‐based samples (corresponding to the samples from top to bottom) after the addition of a solution of Prodan in dichloromethane. F) Relative surface polarity of representative HCP‐based samples.

As a representative sample among the synthesized porous solid catalysts, [edot]_0.34_‐[H]_0.60_‐HCP, detailed spectroscopic characterizations were described in **Figure** **1**. The microstructures of the optimized catalysts were characterized by field emission scanning electron microscopy (FE‐SEM) and transmission electron microscopy (TEM), and the results revealed that the catalysts displayed a hierarchical porous structure (Figure 1A–D; Figure S6, Supporting Information). Compared with the original particulate architecture (Figure S7, Supporting Information), the introduction of linear molecules successfully forms a network structure. Moreover, the high‐angle annular dark‐field scanning transmission electron microscopy (HAADF‐STEM) image and corresponding elemental mappings confirmed the uniform distribution of our dipolar catalysts, which can reasonably be attributed to the successful programming of diversified dipolar fragments into the pre‐designed HCP structure (Figure 1E). To gain a better understanding of the structural composition of porous frameworks, the materials were subjected to characterization with FT‐IR and Raman spectra. The profiles of fresh and the corresponding post‐modified samples were highly comparable, suggesting that the chemical structure was not significantly changed under the electron beam irradiation. In the FT‐IR spectrum of [H]_0.60_‐HCP, the appearance of peaks at 1008 and 1122 cm^−1^ was attributed to the stretching vibration of the S═O bonds of the aromatic sulfonic acid group (**Figure** **2**A; Figures S8 and S9, Supporting Information).^[^ ^29^ ^]^ This was confirmed by the Raman spectrum (Figure S10, Supporting Information).^[^ ^30^ ^]^ The characteristic peak of [edot]_0.34_‐[H]_0.60_‐HCP at 1359 cm^−1^ was ascribed to the C═C asymmetric stretching vibrations of the oxidized thiophene ring.^[^ ^31^, ^32^ ^]^ After oxidation treatment, the new band at 1290 cm^−1^ corresponds to the asymmetric vibrations of the ─SO_2_ group, indicating the formation of sulfone groups.^[^ ^33^, ^34^ ^]^ These results demonstrate the success of programming various predetermined species on the catalyst surface. Additionally, X‐ray photoelectron spectroscopy (XPS) was executed to probe the variation of electronic properties for sulfur elements on the surface of the dipolar catalysts (Figure 2B), whereas the detailed characterization data of other elements are provided in Figures S11–S13 (Supporting Information). In the S 2p XPS spectrum of initial HCP, no characteristic peak of sulfur was observed. Compared with the pure specimen, conspicuous signals of S 2p in [H]_0.60_‐HCP appeared at 168.04 and 169.34 eV, with a peak area ratio of ≈2:1, which was assigned to the S─O bond.^[^ ^35^ ^]^ Correspondingly, the pattern of the S 2p region showed peaks located at 163.45 and 164.75 eV attributed to C─S bonds.^[^ ^36^ ^]^ It was clearly shown that the species with acid sites were successfully introduced on the surface of the catalysts. Furthermore, we can observe that the S 2p peaks of [edot]_0.34_‐[H]_0.60_‐HCP shifted to higher binding energy after the oxidation treatment, indicating that a portion of the sulfur element in the pre‐catalyst is in a more stable aromatization state.^[^ ^37^ ^]^ Besides, the successful modification was also verified by ^13^C MAS NMR spectra (Figure S14, Supporting Information).

To further explore the grafting process by electron beam irradiation, Electron Paramagnetic Resonance (EPR) experiments were also conducted (Figure 2C). The signals of alkyl radicals and hydrogen radicals can be detected during the process of EBI and we verified that the generation of hydrogen radicals comes from the ionization of water molecules.^[^ ^38^, ^39^ ^]^ The results indicate that EBI effectively induces the generation of free radicals from the methylene functional group in 1,3‐benzodioxole, thus facilitating subsequent functionalization processes.^[^ ^40^ ^]^ Indeed, electron beam energy and absorbed dose are crucial parameters in the electron irradiation process as they significantly influence the structure and properties.^[^ ^41^, ^42^ ^]^ Hence, it is highly desirable to choose the appropriate electron beam energy and absorbed dose to ensure the desired irradiation effects are achieved while minimizing potential adverse effects. Taking into account these factors, we performed simulation calculations using the Monte Carlo code FLUKA to study the distribution of electron absorbed dose and penetration depth at the catalyst surface (Figures S21–S26, Supporting Information).^[^ ^43^ ^]^ The findings illustrate that the electron beams possess exceptional penetrating abilities and generate uniform absorbed doses in materials, highlighting their potential as tools for triggering chemical reactions.

To study the chemical and physical properties of catalysts, powder X‐ray diffraction (XRD) patterns were investigated (Figure S15, Supporting Information). The evidence reveals that the samples exhibit distinct amorphous structures. Interestingly, with the increase in modification content, the peaks of the amorphous phase gradually narrow, implying an enhancement of local structural ordering in the amorphous material.^[^ ^44^ ^]^ Moreover, nitrogen sorption isotherms (Figure 2D; Figure S6, Supporting Information) and Brunauer–Emmett–Teller (BET) analysis indicate that the typical samples possess both mesoporous and macroporous structures, with surface areas ranging from 512 to 351 m^2^ g^−1^. The observed decreases were likely ascribed to the occupation of mass and pores by the dipolar decorations. Meanwhile, the thermogravimetric analysis (TGA) shows that all of the catalysts were highly stable below 200 °C, suggesting that conducting chemical reactions under conventional conditions is feasible (Figure S16, Supporting Information). To test the polarity of the resultant catalysts, the characterization of the contact angle was investigated (Figure S17, Supporting Information). The resulting HCP catalyst initially exhibited a contact angle of 106°, indicating that it had a hydrophobic structure. The contact angles of the post‐modified samples [H]_0.60_‐HCP and [edot]_0.34_‐[H]_0.60_‐HCP were 84° and 101°, respectively. While contact angle measurements were related to surface polarity, assessing the correlation between surface polarity and catalytic performance was complex for porous materials. Based on this, the use of organic dyes not only effectively probes polarity but also establishes good relationships, as their electronic energy levels are susceptible to non‐covalent interactions with their surrounding environments.^[^ ^45^, ^46^, ^47^ ^]^ Prodan, a solvatochromic dye, has been used as a microenvironment‐sensitive probe to detect changes in local polarity through Stokes shift, which relies on the environment to stabilize its excited state.^[^ ^48^, ^49^ ^]^ The variation of the polarity was recorded for Prodan adsorbed on the representative HCP‐based materials by fluorescence spectroscopy (Figure 2E). The shortest fluorescence emission wavelength, *λ*_max_ 446 nm, in the homogeneous solution, was consistent with its lower polarity compared to heterogeneous solutions to which various dipolar catalysts were added. Notably, the gradual shift in *λ*_max_, especially for HCP‐based samples modified with oxidized 3,4‐ethylenedioxythiophene, indicates that the surface polarity can be tuned or altered. On the other hand, we observed a significant decrease or even disappearance of the fluorescence intensity (Figure S18, Supporting Information), which was attributed to the formation of twisted intramolecular charge transfer (TICT) states in polar environments.^[^ ^50^ ^]^ These evidences suggest that oxidized 3,4‐ethylenedioxythiophene molecules can act as dipole modulators to modulate the polar environment on the catalyst surface. To further quantify this relationship, we use relative surface polarity for evaluation, as the relationship between pairs of *λ*_max_ values is linear. Specifically, the relative surface polarity values for various dipolar catalysts were derived employing the *λ*_max_ values of the adsorbed Prodan and interpolating the *λ*_max_ values measured in different solvents with known polarities (Figure 2F; Table S2, Supporting Information). The results showed that the relative surface polarity of the prepared catalyst in dichloromethane ranged from 0.298 to 0.374, which falls between the polarities of two different solvents. Consequently, customized dipolar catalysts were expected to serve as reaction media to precisely regulate the surface properties and affect the output of the reaction.

To test the catalytic ability of dipolar catalysts,^[^ ^51^ ^]^ our investigation was then focused on the valorization conversion of biomass platform molecules derived from lignin, α‐hydroxyacetophenones, to synthesize value‐added products of 2‐phenylindole derivatives, which were important in the fine chemical and pharmaceutical industry.^[^ ^52^, ^53^ ^]^ Although this was a concise approach to synthesizing 2‐phenylindoles, previous reports indicate that its applicability was limited to substrates with electron‐donating groups, and the use of simple anilines often resulted in low yields due to the difficulty in controlling the highly reactive aldehyde species generated during the reaction process.^[^ ^54^ ^]^ It is well known that the activity of aldehyde molecules can be regulated by the polarity of the solvent, and molecular dynamics simulations have verified that this process was dipole‐driven.^[^ ^55^ ^]^ On the basis of this consideration, a series of control experiments were carried out to illustrate that the dipolar HCP‐based frameworks can effectively catalyze the reaction (**Table** **1**). When [H]_0.40_‐HCP was used as a catalyst, the desired product **3a** was obtained in 32% of yield, indicating that the customized catalyst holds promise for achieving good results (entries 1–2). Considering the impact of acid‐active sites on conversion efficiency, we then validated that the prepared catalyst was capable of completing such conversion (entries 3–4). This was further supported by the use of temperature‐programmed desorption (TPD), which serves as an informative tool for determining the acidity of multiple acid sites (Figure S19, Supporting Information). Subsequently, various solvents were also screened, and the reaction, when performed in isopropyl acetate (^i^PrOAc) afforded the product with a yield of 51% (Table S3, Supporting Information, entry 5). To our delight, catalysts decorated with the dipolar species exhibited good capability for the reaction (entry 6). In contrast, we observed a significant jump in reactivity with the aid of [edot]_0.34_‐[H]_0.60_‐HCP. The yield of the desired product reached an impressive 94% (entry 7). This is indicative of the diverse dipolar environments that were effective in promoting catalytic performance. Further modulation of the polarity environment leads to a slight decrease in efficiency, but compared to the unmodified matrix, these functional groups still accelerate catalysis (entry 8). Apparently, these results indicate that the catalysts decorated with the tunable microenvironments have the ability to generate positive impacts, possibly due to the specific spatial structure facilitating enhanced favorable interactions between catalyst and substrate.

**Table 1:** Condition optimization for the synthesis of 2‐phenylindole from aniline and α‐hydroxyacetophenone.

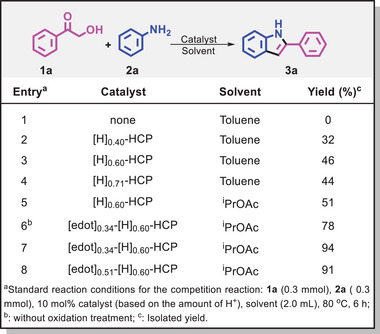			
Entry^a)^	Catalyst	Solvent	Yield [%]^c)^
1	None	Toluene	0%
2	[H]_0.40_‐HCP	Toluene	32%
3	[H]_0.60_‐HCP	Toluene	46%
4	[H]_0.71_‐HCP	Toluene	44%
5	[H]_0.60_‐HCP	^i^PrOAc	51%
6^b)^	[edot]_0.34_‐[H]_0.60_‐HCP	^i^PrOAc	78%
7	[edot]_0.34_‐[H]_0.60_‐HCP	^i^PrOAc	94%
8	[edot]_0.51_‐[H]_0.60_‐HCP	^i^PrOAc	91%

^a)^ Standard reaction conditions for the competition reaction: **1a** (0.3 mmol), **2a** (0.3 mmol), 10 mol.% catalyst (based on the amount of H^+^), solvent (2.0 mL), 80 °C, 6 h;

^b)^ Without oxidation treatment;

^c)^ Isolated yield.

Given the excellent performance of our catalysts, we sought to establish the broad applicability of dipolar catalysts by investigating their potential to catalyze new types of reactions. In this light, glyoxal was considered the optimal substrate, as the isomerization process was highly sensitive to changes in the polarity of the environment.^[^ ^55^, ^56^ ^]^ Gratifyingly, we found that the family of [edot]_0.51_‐[H]_0.40_‐HCP was capable of carrying out the Henys rearrangement reaction between aniline and glyoxal. A series of optimization experiments confirmed that the prepared dipolar catalyst was suitable for this reaction (Table S4, Supporting Information). Specifically, with the use of the catalyst [edot]_0.51_‐[H]_0.40_‐HCP, the optimal yield of the desired product reaches 89%. To the best of our knowledge, there have been no reports so far regarding the direct synthesis of ethyl phenylglycinate using glyoxal as a substrate. The results allow us to conclude that the dipolar catalysts provide an environment for regulating the reactivity of aldehydes and amines, thus offering opportunities for efficient effective control of highly reactive molecules.

**Figure 3:**
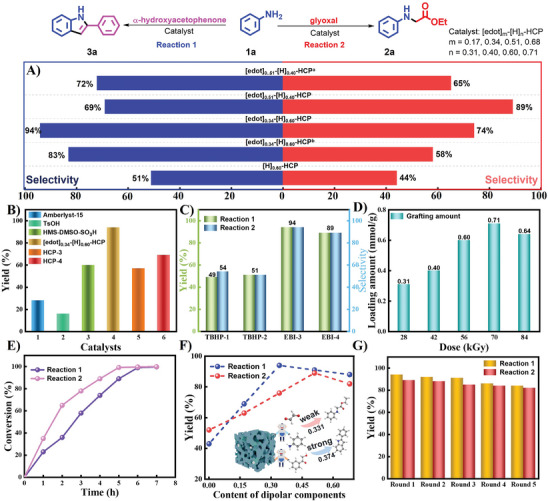
A) The selectivity of model reactions catalyzed by [edot]_m_‐[H]_n_‐HCP catalysts, and the marked a and b indicate samples without oxidation treatment. B) Effect of various catalysts on the yield of 2‐phenylindole from aniline and α‐hydroxyacetophenone. C) Compare the effects of electron beam irradiation and traditional chemical processes on the yield and selectivity of different reactions. D) The influence of different irradiation absorption doses on the loading of catalytically active species. E) The conversion of the two reactions uses [edot]_0.34_‐[H]_0.60_‐HCP and [edot]_0.51_‐[H]_0.40_‐HCP as catalysts, respectively. F) The effect of the content of dipolar components on chemical reactions and the inset showed the different forces and relative polarity values displayed by dipolar catalysts on different substrates. G) Recycling experiments with [edot]_0.34_‐[H]_0.60_‐HCP and [edot]_0.51_‐[H]_0.40_‐HCP catalysts under optimal conditions.

We subsequently set out to investigate how modifying the environment surrounding the catalytic sites can discriminate between the two reaction pathways and maximize the selectivity and yield of the corresponding target products (**Figure** **3**). Interestingly, it can be found that the selectivity values of all catalysts modified with dipolar groups are significantly higher than those of unmodified catalysts (Figure 3A). Although the HCP‐based families without oxidation treatment did not show the expected results, they are still far superior to the catalysts only equipped with active sites. This suggests that synergistic effects between reaction participants and modulators bearing dipolar species can enhance selectivity. Meanwhile, the [edot]_0.34_‐[H]_0.60_‐HCP family is capable of conducting a reaction between α‐hydroxyacetophenone and aniline, with a selectivity value of 94%, which is higher than the 74% provided by [edot]_0.51_‐[H]_0.40_‐HCP under standard conditions. However, the [edot]_0.51_‐[H]_0.40_‐HCP plays a dominant role in the reaction between aniline and glyoxal, affording a selectivity value of 89%. These results indicate that both reactivity and selectivity can be precisely tuned through interactions between modulators and reaction participants, which is consistent with the variations in polarity.

To further illustrate the advantages of our catalysts, we screened various reference catalysts for the synthesis of **3a** (Figure 3B), and another reaction was shown in Table S4 (Supporting Information). The results show that the efficiency of all heterogeneous catalysts was superior to that of homogeneous catalysts (TsOH, *p*‐toluenesulfonic acid). Moreover, the commercially available Amberlyst‐15 catalyst only provides products with 24% yields. The catalyst [H]_0.60_‐HCP, which was only equipped with active sites and lacked the dipolar component, could only provide the target product in moderate yield. In comparison, our previously developed reference catalyst, HMS‐DMSO‐SO_3_H, which incorporates dipolar moieties also exhibits acceptable catalytic performance, affording the desired product in 60% yield.^[^ ^10^ ^]^ These findings demonstrate that dipolar environments were indeed proven to be effective in enhancing catalytic performance. Besides, we have verified that catalysts prepared from multifunctional monomers outperform dipolar catalysts prepared from single‐functional monomers (HCP‐2 and HCP‐3, Figure S4, Supporting Information) in terms of catalytic performance. This implies that the introduction of a two‐site structured molecule was the key factor affecting the performance of catalysts. Despite the fact that the prepared functionalized dipolar catalysts have shown tremendous potential in catalytic applications, the effectiveness of catalysts prepared through traditional chemical processes fails to meet expectations. As shown in Figure 3C, the analogous dipolar catalysts prepared through the conventional chemical grafting with free radical initiators proceeded sluggishly, with yields of only 49% and 51%, respectively, which cannot compare to the catalysts prepared by EBI technology. In fact, when using AIBN (azodiisobutyronitrile) or TBHP (*^t^* butyl hydroperoxide) as initiators, the maximum grafting capacity of catalysts with acid functional groups was 0.23 mmol g^−1^, resulting in their poor catalytic ability. We observed that the absorbed dose has a pronounced impact on the grafting amount, which in turn affects its catalytic performance (Figure 3D). When the radiation dose was 70 kGy, the grafting amount reached a remarkable 0.71 mmol g^−1^, which was confirmed by elemental analysis (Table S1, Supporting Information). Apparently, the use of the EBI technique in the preparation of dipolar catalysts provides a favorable assurance for maximizing catalytic efficiency. Moreover, we recorded the conversion of aniline under standard conditions in both reactions (Figure 3E). In this process, we also verified the generation of aryl aldehyde intermediates, which was consistent with previous reports.^[^ ^54^ ^]^ We also explore the effect of the content of dipolar components on catalytic efficiency (Figure 3F). This implies that we can tailor dipolar catalysts with the desired polarity by considering the requirements of different reactions. Furthermore, the recyclability was a distinct advantage of dipolar catalysts as a class of heterogeneous catalysts over homogeneous ones. The results indicated that the highly active material was preserved throughout recycling in both reactions (Figure 3G).

**Scheme 2:**
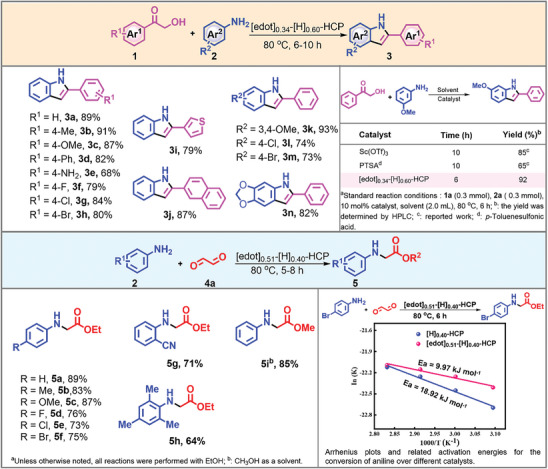
The substrate scope for the acid‐catalyzed synthesis of 2‐arylindoles and ethyl phenylglycinate derivatives. The inserted table compared the differences between this work and traditional methods, and the inset showed the Arrhenius plots of the reaction under standard conditions.

The performance of [edot]_0.34_‐[H]_0.60_‐HCP was then examined in the synthesis of a diverse indole derivatives shown in **Scheme** **2**. Various substituents patterns in the aryl ring of α‐hydroxyacetophenones (Figure S5, Supporting Information) efficiently offer the corresponding products (**3a–3h**) with good to excellent yields, regardless of the electronic effect or steric effect. The aromatic substrates (**3i–3j**) can also be delivered smoothly to the target product in 79% and 87% yields, respectively. Generally, electron‐rich aromatic amines play a positive role in mediating the reactivity of α‐hydroxy ketones, resulting in the desired products with a high yield (**3k**). It is worth noting that anilines with electron‐withdrawing groups in the *para* position are also well tolerated in this reaction (**3l–3m**). Moreover, the heteroaromatic substrates (**3n**) can also be successfully converted into the expected products with 82% yield. Compared with the reported studies, the developed dipolar catalytic system not only facilitates the efficient assembly of indole derivatives but also overcomes the drawbacks associated with the use of expensive metals and complex additives.^[^ ^54^ ^]^ We have also extended the application of our catalysts [edot]_0.51_‐[H]_0.40_‐HCP in the synthesis of ethyl phenylglycinate derivatives under standard conditions. A wide range of para‐substituted phenylamine derivatives (H, Me, OMe, F, Cl, Br) showed moderate to good yields (**5a–5f**). It can be observed that electron‐donating substituents have an advantage over electron‐withdrawing groups in terms of efficiency. *Ortho*‐substituted aniline bearing a cyano group was also well‐tolerated in this reaction, affording the desired product in 71% yield (**5g**). Interestingly, the tri‐substituted substrate can also be delivered into the target product with an acceptable yield (**5h**). When methanol was used as the solvent, the product could also be obtained with a yield of 85% (**5i**).

In addition, the apparent activation energies (*E_a_*) for this reaction were calculated from the Arrhenius equation. The calculated value of *E_a_* for [edot]_0.51_‐[H]_0.40_‐HCP was 9.97 kJ mol^−1^, which was lower than the value of 18.29 kJ mol^−1^ for [H]_0.40_‐HCP.

**Figure 4:**
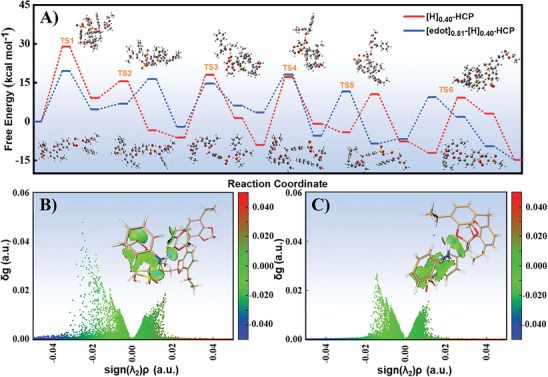
Theoretical calculations reveal the reaction pathways of Henys rearrangement over different dipolar catalysts. 4A) The free energy profiles and corresponding intermediate configurations in the [edot]_0.51_‐[H]_0.40_‐HCP and [H]_0.40_‐HCP system; Sign(λ_2_)ρ colored isosurfaces of δg^inter^ = 0.004 a.u. corresponding to IGMH analysis of (4B) [edot]_0.51_‐[H]_0.40_‐HCP and (4C) [H]_0.40_‐HCP catalysts.

To elucidate the underlying mechanism and assess the impact of dipolar catalysts with specific microenvironments on facilitating the reaction transition state (TS), density functional theory (DFT) calculations have been implemented. In conjunction with the reported work,^[^ ^54^ ^]^ a possible mechanism is depicted in Figure S20 (Supporting Information). In light of the abundance of repetitive dipole units present in the dipolar catalyst, a reasonable simplification has been applied to the structural models that exhibit negligible influence on the reaction environment (Figures S27, S30, and S31, Supporting Information). In the system of aniline and glyoxal catalyzed by [edot]_0.51_‐[H]_0.40_‐HCP, a noticeable decrease in the activation barrier for the nucleophilic attack (TS1) step was observed compared to the system catalyzed by [H]_0.40_‐HCP (**Figure** **4**A). This indicates that activated glyoxal molecules can react smoothly with aniline in a relatively strong dipolar environment. Notably, the specific spatial environment in [edot]_0.51_‐[H]_0.40_‐HCP can promote the catalytic species to function better by comparing the value of the free energy barrier in the T3‐state. Therefore, dipolar catalysts with diverse microenvironments show unique advantages in stabilizing intermediates, implying that precise control of the microenvironment around the dipolar active site can achieve precise control of catalytic reactions. To reveal non‐covalent interactions between the reactants and dipolar catalysts in chemical systems, the independent gradient model based on Hirshfeld partition (IGMH) analysis was established.^[^ ^57^ ^]^ Isosurfaces of the δ_g_ function with varying values were utilized to display interaction regions of different strengths and distinguish the interaction types through the colors corresponding to the values of the mapped function sign(λ_2_)ρ.^[^ ^57^ ^]^ The highlighted green isosurfaces in specific structures effectively emphasize the regions exhibiting significant mutual interaction between the catalyst and the reaction participants (Figure 4). The wider range of isosurfaces in Figure 4B suggests a broader region of interaction, which is consistent with the observed experimental trend. Notably, the upgraded catalyst [edot]_0.51_‐[H]_0.40_‐HCP exhibits the highest value (sign(λ_2_)ρ = 0.02298) compared to the primary catalyst [H]_0.40_‐HCP (sign(λ_2_)ρ = 0.01472) (Figure 4C), indicating the catalyst is able to provide multiple interactions with the reaction substrates.^[^ ^7^ ^]^ These non‐covalent interactions were also verified in the reaction between aniline and α‐hydroxyacetophenone (Figures S28 and S29, Supporting Information). Meanwhile, the intermolecular Charge Decomposition Density (CDD) analysis reveals that modulators enhance the interaction between the catalyst and the intermediate. These findings further confirm that the multipolar regions within dipolar catalysts are capable of modulating catalytic performance through a diverse range of favorable non‐covalent interactions.

## 3 Conclusion

In summary, we have developed a facile and effective method to construct a tunable dipolar microenvironment within the catalyst pores via EBI technology for modulating the non‐covalent interactions between the catalyst and reactant molecules. Experimental and DFT calculation results revealed that the combination of multiple favorable interactions act in tandem to stabilize the transition states, thereby improving the performance of the catalyst. Specifically, the creation of a unique dipolar environment surrounding the active site has greatly enhanced the efficiency and selectivity in the Henys rearrangement reaction in heterogeneous systems. The ability resembles that of enzymes, which utilize dynamic interactions with substrates to achieve specific transformations. This work not only provides a profound understanding of the influence of non‐covalent interactions between dipole‐localized environments and substrates on the efficiency and selectivity of chemical transformations, but also offers guidance for the rational design of catalysts that are more efficient and practical.

## Conflict of Interest

The authors declare no competing financial interest.

## Supporting information

Supporting Information

## Data Availability

The data that support the findings of this study are available from the corresponding author upon reasonable request.
