# Supplementary material for: Dipolar Microenvironment Engineering Enabled by Electron Beam Irradiation for Boosting Catalytic Performance
Source: Adv Sci (Weinh). 2024 Jun 11;11(30):2401562. doi: 10.1002/advs.202401562 (PMC11321705; doi:10.1002/advs.202401562)
Supplement: Supplementary file 1 — Supporting Information [file ADVS-11-2401562-s001.pdf]

ADVANCED SCIENCE
Open Access

Supporting Information

for *Adv. Sci.*, DOI 10.1002/advs.202401562

Dipolar Microenvironment Engineering Enabled by Electron Beam Irradiation for Boosting Catalytic Performance

Zhiyan Chen, Shuai Hao, Haozhe Li, Xiaohan Dong, Xihao Chen, Jushigang Yuan, Alexander Sidorenko, Jiang Huang and Yanlong Gu**

Supporting Information

Dipolar Microenvironment Engineering Enabled by Electron Beam Irradiation for Boosting Catalytic Performance

Zhiyan Chen, Shuai Hao, Haozhe Li, Xiaohan Dong, Xihao Chen, Jushigang Yuan, Alexander Sidorenko, Jiang Huang,* Yanlong Gu*

Table of contents

1. Experimental Procedures 3

1.1 General information 3

1.2 Preparation of HCP-based catalyst 3

1.3 Typical procedure for the synthesis of α-hydroxyacetophenone derivatives 4

1.4 Measurement of fluorescence spectra 5

2 Results and Discussion 5

2.1 Pore distribution of the catalysts 5

2.2 SEM and corresponding EDS images of different types of catalysts 6

2.3 The FT-IR and Raman spectra of representative catalysts 6

2.4 XPS spectra of the prepared catalysts 7

2.5 Elemental analysis of prepared catalysts 8

2.6 ^{13}C MAS NMR spectrum of representative catalysts 9

2.7 XRD patterns of the catalysts 9

2.8 TGA test of the catalysts 9

2.9 Contact angle test of the catalysts 10

2.10 Fluorescence spectroscopy of the catalysts 10

2.11 TPD test of the catalysts 11

2.12 Condition optimization 11

2.13 Proposed mechanism for the formation of 3a and 5a 12

2.14 Simulation data 12

2.15 DFT calculations for the two reaction mechanisms 15

2.16 Characterization data of all new compounds 16

2.17 ^{1}H NMR and ^{13}C NMR spectra (3a) 21

3 References 44

1. Experimental Procedures

1.1 General information

Unless specially indicated, all chemical reagents and solvents were purchased from available commercial suppliers and used without further purification. Fourier transform infrared (FT-IR) was carried out on Bruker Compass VERTEX 70 with the wavenumber range of 4000–400 cm^{-1}. The X-ray photoelectron spectra (XPS) data were obtained on AXIS-ULTRA DLD-600W using C 1s (284.8 eV) as the reference line. Thermogravimetric analysis was performed on TGA 2950 by heating from room temperature to 800 °C in N_2 atmosphere. Solid ^{13}C NMR spectra of HCP, $[H]_n$-HCP and $[edot]_m$-$[H]_n$-HCP materials were measured on Bruker 400M. Elemental analysis (EA) was conducted in Vario el cube by using CHNS mode. The Brunauer–Emmett–Teller (BET) surface areas of representative samples were assessed employing Micromeritics ASAP 2020 M. The data of high-resolution mass spectra (HRMS) were acquired on Bruker Compass Data Analysis 4.0. The power X-ray diffraction (XRD) patterns were collected on a Rigaku SmartLab-SE X-ray diffractometer. Field-emission scanning electron microscope (FSEM) and transmission electron microscopy (TEM) were completed on FEI Sirion 200 and FEI Tecnai G2 F30 TMP TEM, respectively. The high-angle annular dark-field scanning transmission electron microscopy (HAADF-STEM) image and corresponding elemental mappings were performed on a JEM-2100F (Japan) scanning transmission electron microscope. The Chemical adsorption-temperature programmed desorption (TPD) curves were secured from BSD-Chem C200 with a heating rate of 10°C / min. The fluorescence spectra were measured using an RF-6000 PC spectrophotometer. The Electron Paramagnetic Resonance (EPR) experiments were carried out on a Bruker EMXmicro-6/1. The Bruker Advance III 400 MHz spectrometer was used to record the ^{1}H and ^{13}C NMR spectra of organic compounds. Chemical shifts are expressed in ppm with respect to the signal of tetramethylsilane in $CDCl_3$ or DMSO-d_6 as the reference. The AB10.0 accelerator (Wuxi El Pont, China) and EBA-200 (COMET, Switzerland)) were utilized to implement irradiation experiments. In this study, FLUKA version 4–2.1, installed on ubuntu 18.04, was utilized for the simulation of various parameters for front-end electron collection. All quantum chemical calculations, including geometry optimizations and frequency calculations, were performed at the B3LYP/6-31G* level with the D3 version of Grimme's dispersion with Becke-Johnson damping in the quantum chemical package Gaussian 09.

1.2 Preparation of HCP-based catalyst

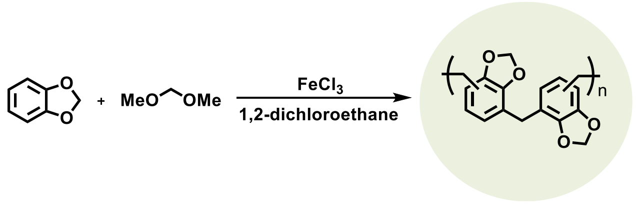

Figure S1. Schematic preparation procedure of HCP.

The preparation method of hypercross-linked polymer (HCP) was based on reported literature.[1] The aromatic monomer (1,3-benzodioxole, 0.01 mol, 1.22 g), cross-linking agent (dimethoxymethane, 0.03 mol, 2.28 g) and the catalyst ($FeCl_3$, 0.03 mol, 4.88 g) were dissolved in 1,2-dichloroethane (DCE, 30 mL) solvent. Subsequently, the mixed solution was heated at 45 °C for 6 h to ensure thorough mixing and form a network structure. Then, it was heated at 80 °C for 24 h under reflux conditions to complete the condensation reaction and produce hypercross-linked polymer. The obtained solid product was washed several times with methanol until the filtrate was nearly colorless. Finally, the product was Soxhlet extracted with methanol for 48 h, followed by drying at 80 °C for 12 h in a vacuum oven.

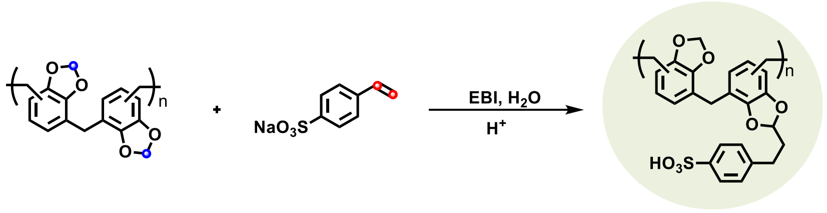

Figure S2. Schematic preparation procedure of $[H]_n$-HCP.

1) The process for preparation of solid acid catalysts by electron beam irradiation: in a custom-made 108 mL square quartz dish, HCP (0.20 g) was treated with 4-vinylbenzenesulfonic acid sodium salt (1 mmol, 0.21 g) in 1.5 mL H_2O at room temperature. The mixture was evenly spread on a quartz dish, maintaining its thickness of 1-2 mm. The irradiation operation was carried out by an EBLAB-200 benchtop self-shielded electron accelerator. Initially, irradiation parameters were set on the operable screen, comprising the air gap (45 mm), irradiation dose (300 kGy), voltage (200 kV), and velocity (9 m/min). Place the prepared sample precursor into a specific mold, position it at the specified place of the accelerator, and close the chamber door. To enhance efficiency and minimize the impact of

ozone generation on the reaction during irradiation, we control a suitable vacuum environment for the reaction by introducing N_2 during the irradiation process. Subsequently, a high voltage (200 kV) is applied and maintained to provide continuous energy. The electron beam automatically emits electrons to trigger reactive ions or free radicals from the precursor sample, with the whole operation completed within 5 s. By repeating this operation process, the catalyst reaches the required accumulated absorption dose. The irradiated sample was washed with H_2O during the process of filtration and the resulting product was dried in a vacuum oven at 80 ^oC overnight. A rang of solid acid catalysts with varying contents can be achieved under standard conditions. In addition, the specifications of the corresponding products can be expanded by more than 10 times under the conditions of a 10MeV electron accelerator, mainly due to the influence of penetration depth.

2) Chemical method experimental steps: In a 100 mL round bottom flask, 4-vinylbenzenesulfonic acid sodium salt (5 mmol, 1.03 g) was mixed with the HCP (0.50 g) in 20 mL H_2O, then the *tert*-butyl hydroperoxide (TBHP, 0.09 g, 1 mmol) was added. The reactor equipped with magnetic stirrer was then sealed to react at 60 ^oC for 6 h. After the filtration operation, wash the product with water and ethanol respectively (30 mL x 3 each) and place it in a vacuum oven at 80°C overnight. Meanwhile, we use AIBN as an initiator to prepare the product, the process of which is similar to the above but the difference lies in the solvent being methanol.

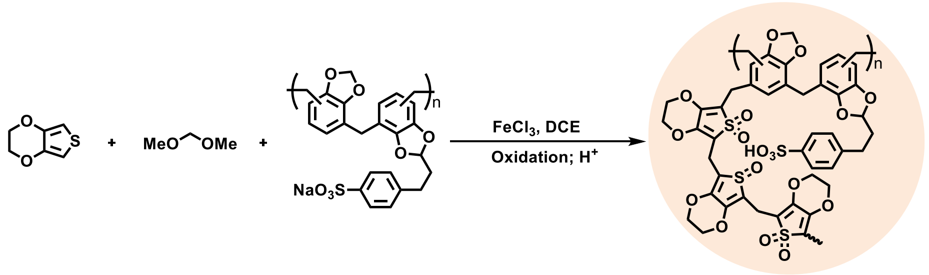

Figure S3. Schematic preparation procedure of $[edot]_m$-$[H]_n$-HCP.

In a 250 mL round bottom flask, 3,4-ethylenedioxythiophene (0.01 mol, 1.43 g) was reacted with $[H]_{0.60}$-HCP(1.63 g) in the presence of crosslinker (dimethoxymethane, 0.03 mol, 2.28 g) and catalyst ($FeCl_3$, 0.03 mol, 4.88 g) in 1,2-dichloroethane (30 mL). The mixture was heated at 45 °C for 6 h and then heated at 80 °C for 20 h under reflux conditions, resulting in a black hypercrosslinked polymer. The obtained product was washed several times with methanol until the filtrate was almost colorless. It was then Soxhlet extracted with methanol for 48 hours, followed by drying at 80 °C for 12 h in a vacuum oven. The obtained products were mixed with commercially available oxidizing agent 3-chloroperoxybenzoic acid (10 mmol, 1.72 g), and stirred in chloroform (100 mL) at room temperature for 6 h. During the filtration operation, the product was washed with chloroform (20 mL × 3). The product was placed in an acidic solution (pH=1), stirred at room temperature for 6 h, and then washed with pure water (30 mL × 3). Simultaneously, electron beam irradiation technology was used to eliminate any potential residual impurities. It was then dried in a vacuum oven at 80°C for 12 h to obtain the final catalyst.

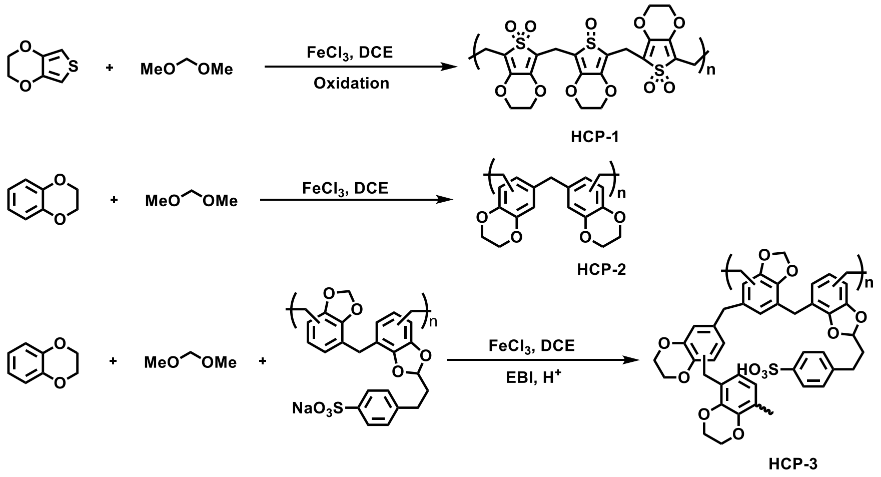

Figure S4. Schematic preparation procedure of other reference materials.

The preparation processes of other materials are based on the methods described above.

1.3 Typical procedure for the synthesis of α-hydroxyacetophenone derivatives

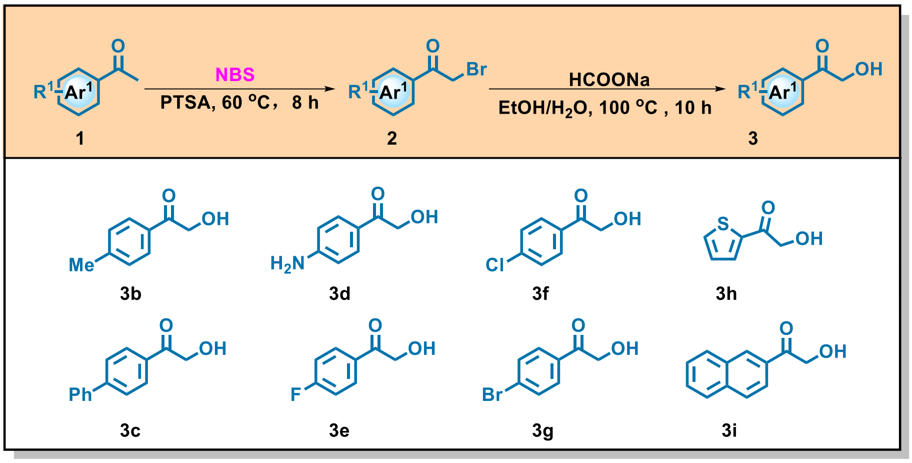

Figure S5. Synthesis of α-hydroxyacetophenone derivatives.[2]

1) In a 250 mL round bottom flask fitted with a reflux condenser, the acetophenone derivatives **1** (10 mmol) were mixed *N*-bromosuccinimide (11 mmol) and *p*-toluenesulfonic acid (1 mmol) in ethyl acetate (EtOAc, 100 mL). The mixture is stirred at 60°C for 8 h, with the whole process monitored by TLC. Once the reaction has finished, it is allowed to cool to ambient temperature, and the bulk of the solvent is then eliminated via rotary evaporation. The product was further purified by column chromatography to yield the 2-bromoacetophenone derivative (eluting solution: petroleum ether/ethyl acetate = 20/1 (v/v)). Corresponding compounds are prepared following the standard procedure.

2) In a 250 mL round bottom flask equipped with a reflux condenser, 2-bromoacetophenone compounds **2** (10 mmol) along with sodium formate (16 mmol) were dissolved in aqueous ethanol ($EtOH/H_2O$ = 2/1 (v/v)), followed by a 10 h reflux in an oil bath. After completion, the mixture was diluted with water (50 mL) and an extraction process was conducted with ethyl acetate (30 mL × 3). The combined organic layers were subjected to drying over Na_2SO_4, then filtered and concentrated by rotary evaporation. The residue was purified by column chromatography to produce target product ((eluting solution: petroleum ether/ethyl acetate = 30/1 (v/v))). The fabrication of analogous compounds continued as per the standard methodology.

1.4 Measurement of fluorescence spectra

According to the reported method[3], the Prodan (0.004 mol/L) solution was prepared. Each HCP-based powder (0.10 g), including HCP, $[H]_{0.60}$-HCP and $[edot]_{0.34}$-$[H]_{0.60}$-HCP, was stirred with 20 mL Prodan solution for 12 h. The emission spectra of the supernatant were recorded using an RF-6000 PC spectrometer with an excitation wavelength of 365 nm.

2 Results and Discussion

2.1 Pore distribution of the catalysts

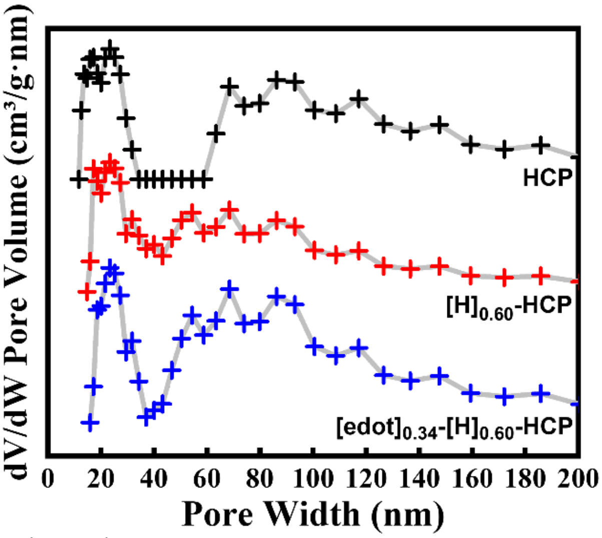

Figure S6. Pore size distributions of representative samples.

2.2 SEM and corresponding EDS images of different types of catalysts

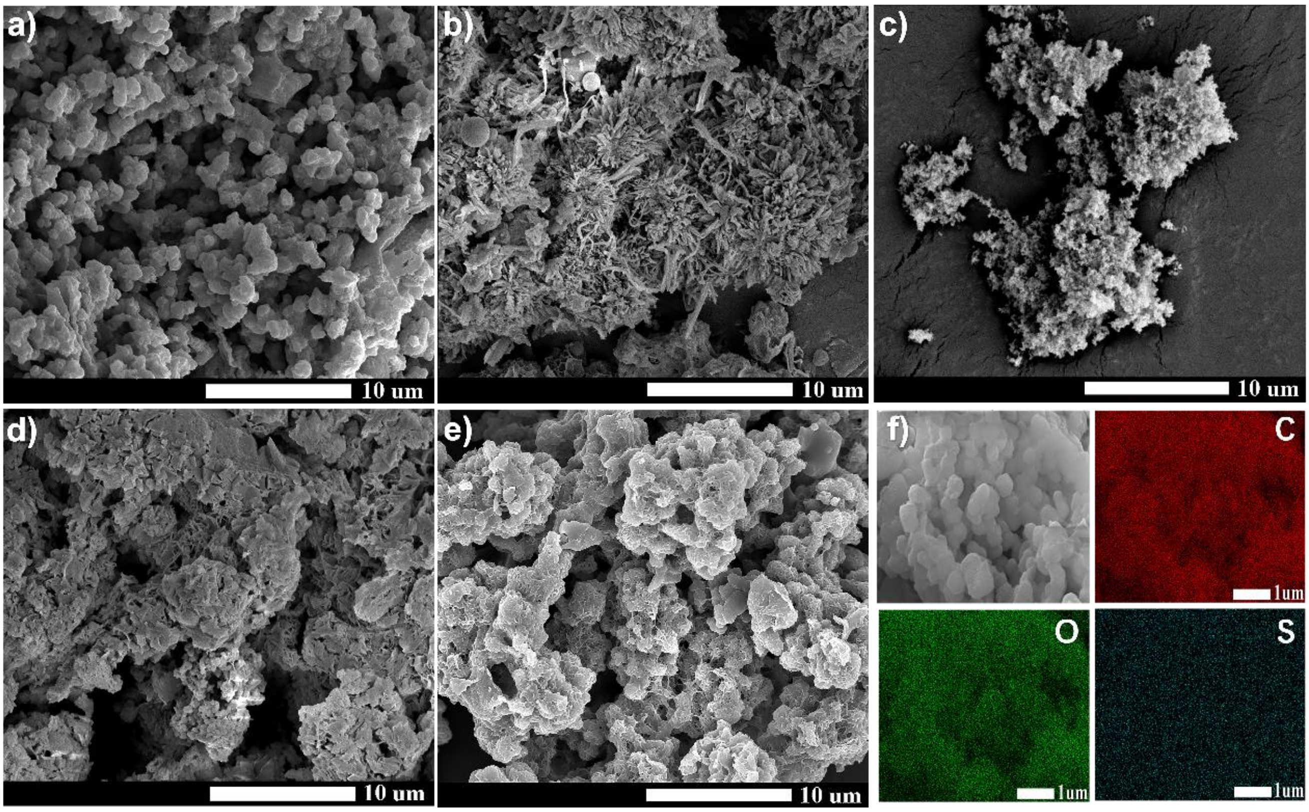

Figure S7. Representative SEM images of different types of catalysts. a) original catalyst of HCP; b) reference catalyst of HCP-1; c) reference catalyst of HCP-3; d) reference catalyst of HCP-2; e) $[edot]_{0.51}$-$[H]_{0.4}$-HCP; f) $[H]_{0.6}$-HCP image and corresponding elemental distribution mappings, including C, O, S elements.

2.3 The FT-IR and Raman spectra of representative catalysts

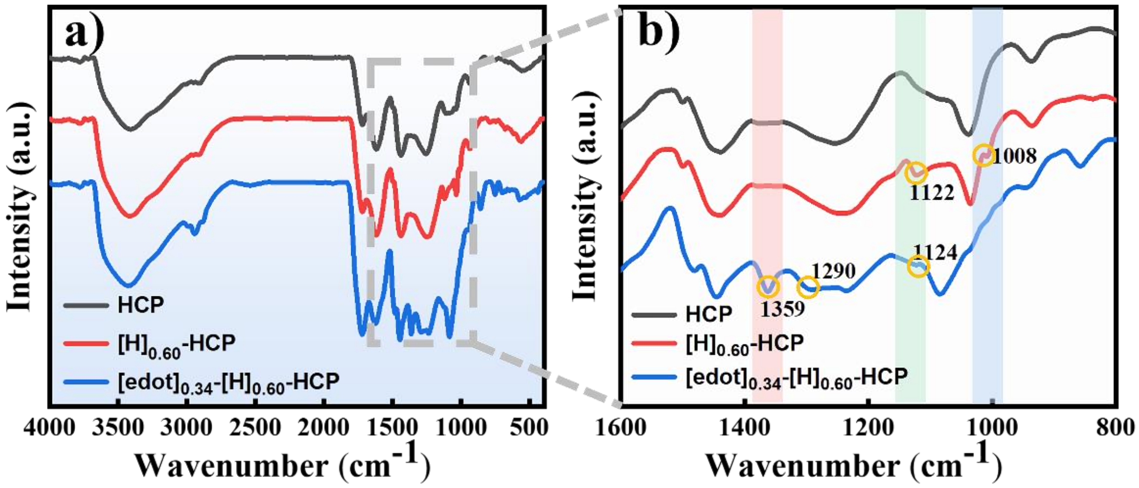

Figure S8. a) The FT-IR spectra of three representative samples; b) magnification image of FT-IR spectra.

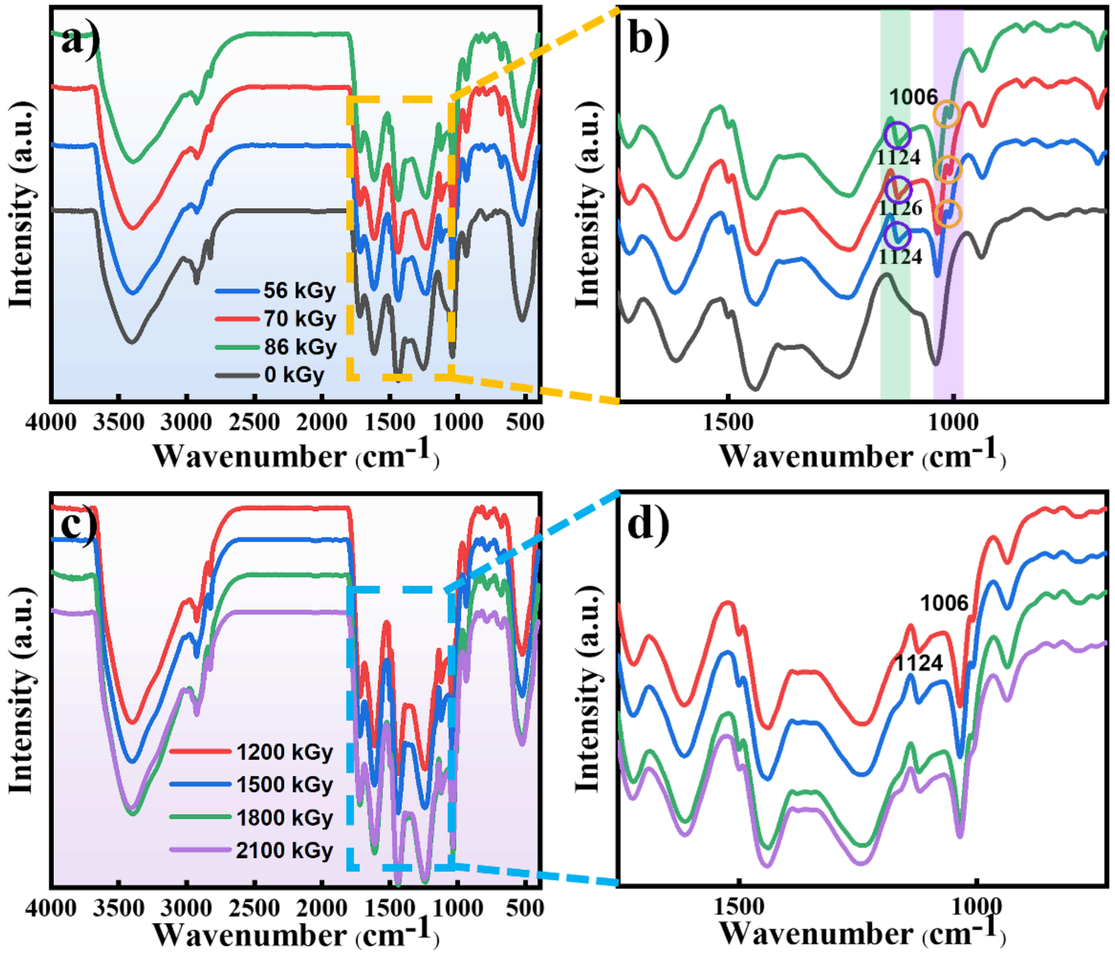

Figure S9. The FT-IR spectra of the original HCP material under different radiation absorption doses. a-b) The variation of FT-IR spectra and magnification image at 10 MeV; c-d) show the changes at 0.2 MeV.

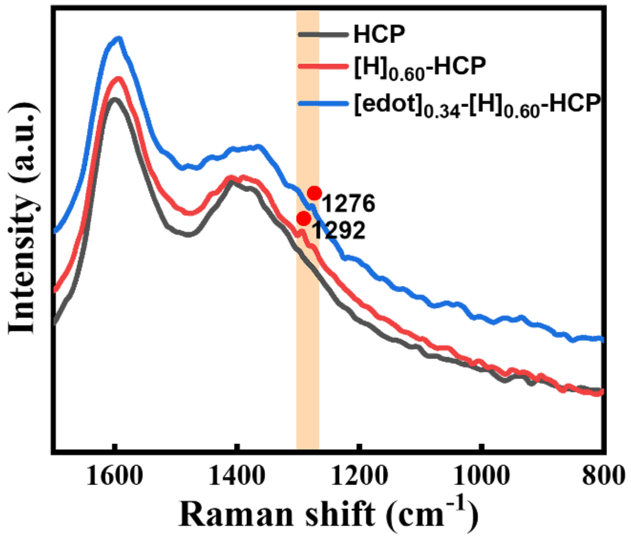

Figure S10. The Raman spectra of three representative samples.

2.4 XPS spectra of the prepared catalysts

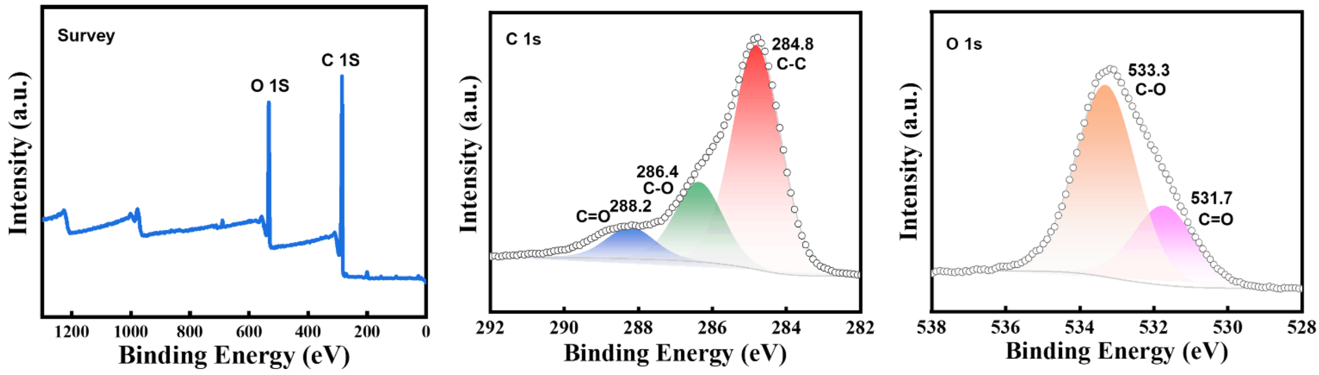

Figure S11. XPS spectra of HCP, including survey spectra and magnifications of the C 1s, O 1s region.

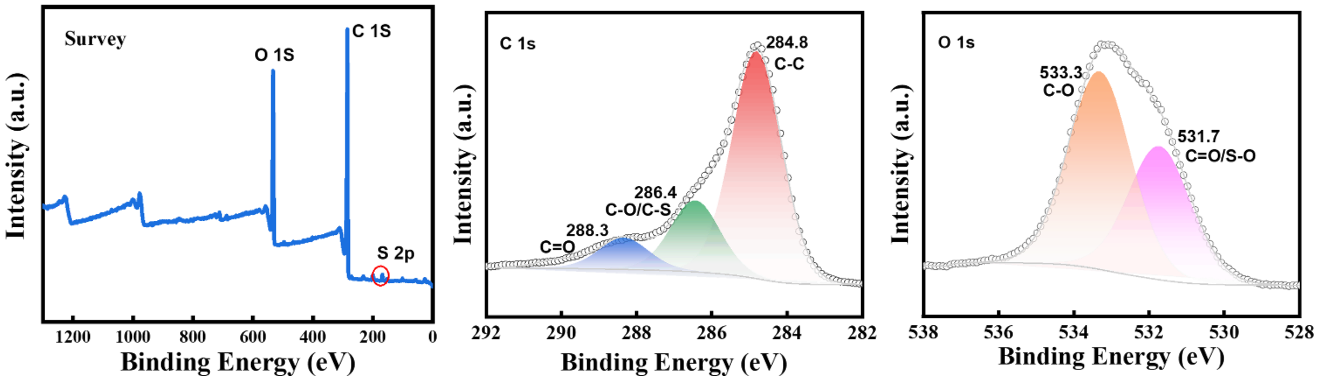

Figure S12. XPS spectra of $[H]_{0.60}$-HCP, including survey spectra and magnifications of the C 1s, O 1s region.

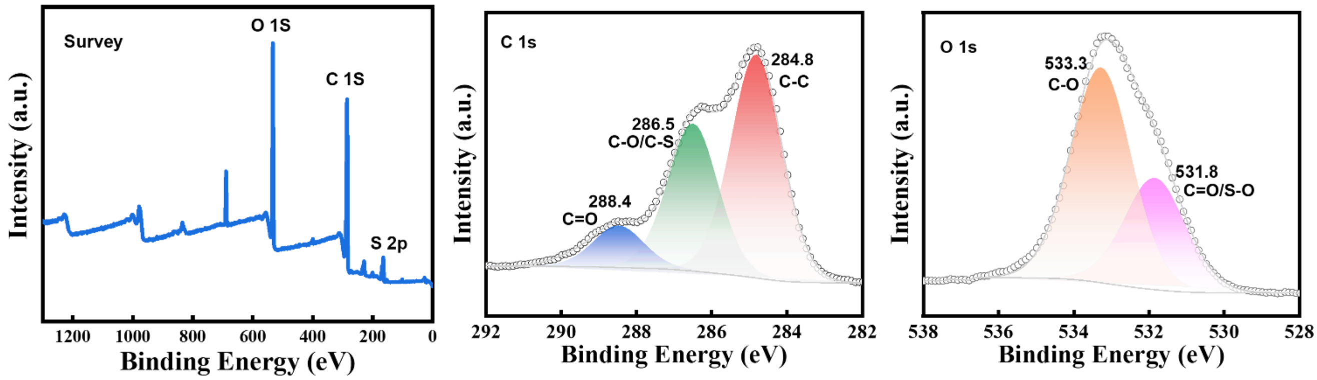

Figure S13. XPS spectra of $[edot]_{0.34}$-$[H]_{0.60}$-HCP, including survey spectra and magnifications of the C 1s, O 1s region.

2.5 Elemental analysis of prepared catalysts

Table S1. Properties of the different dipolar catalysts.

Catalyst	Method	Radiation dose (kGy)	C (%)	H (%)	S (%)[a]
HCP	2mg80s	0	43.06	3.016	0.003
$[H]_n$-HCP	2mg80s	28	55.58	3.967	0.992
$[H]_n$-HCP	2mg80s	42	55.15	3.927	1.281
$[H]_n$-HCP	2mg80s	56	57.02	4.157	1.914

$[H]_n$-HCP	2mg80s	70	61.75	4.116	2.286[d]
$[H]_n$-HCP	2mg80s	84	59.61	4.119	2.048
$[H]_n$-HCP[b]	2mg80s	---	45.92	2.903	0.442
$[H]_n$-HCP[c]	2mg80s	---	52.21	3.615	0.748
$[edot]_m$-$[H]_n$-HCP	2mg80s	70	76.32	5.456	12.086

[a]: All calculations are based on the content of S; [b]: AIBN [c]: TBHP; [d]: maximum value.

2.6 ^{13}C MAS NMR spectrum of representative catalysts

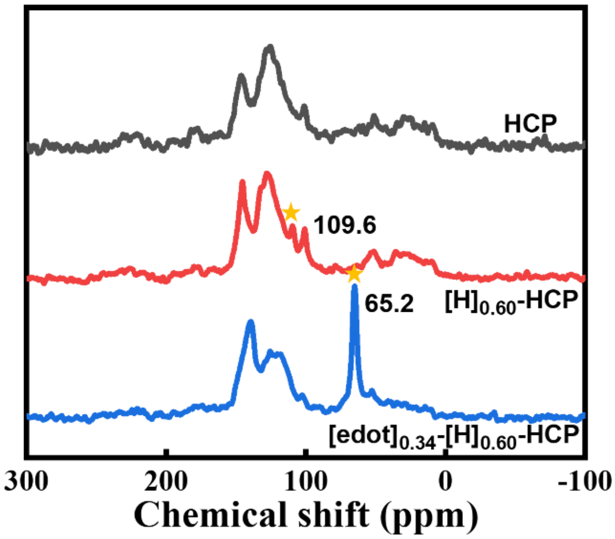

Figure S14. Solid-state ^{13}C NMR spectra of three representative samples.

2.7 XRD patterns of the catalysts

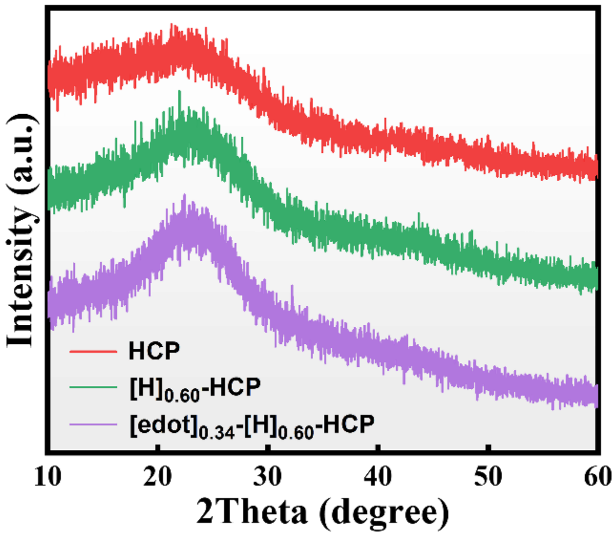

Figure S15. a) Powder XRD patterns of representative samples.

2.8 TGA test of the catalysts

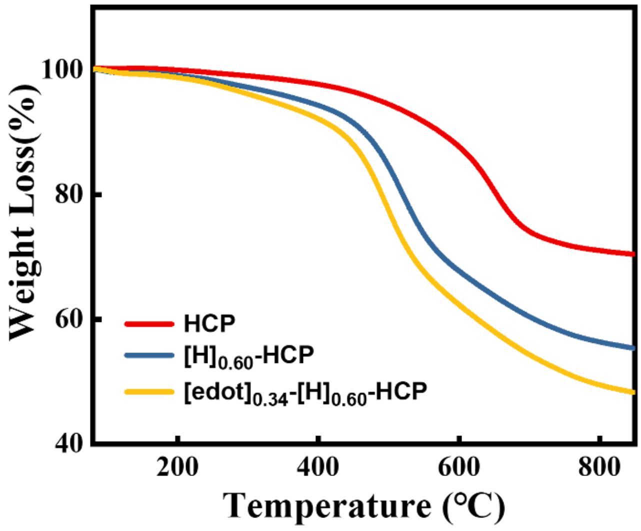

Figure S16. Thermogravimetric weight loss of representative samples.

2.9 Contact angle test of the catalysts

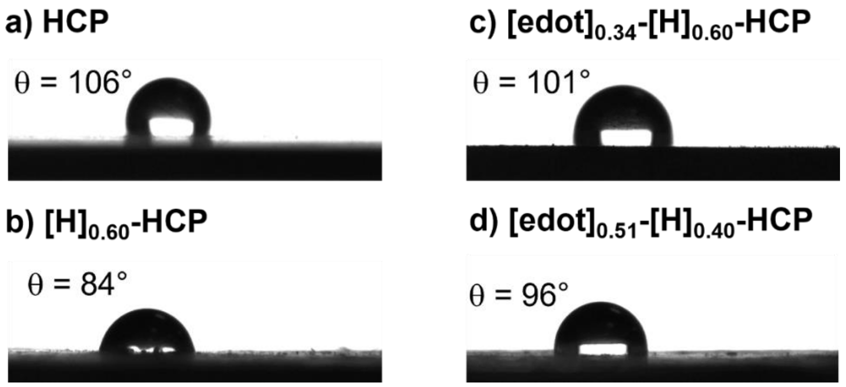

Figure S17. Contact angle testing of representative samples.

2.10 Fluorescence spectroscopy of the catalysts

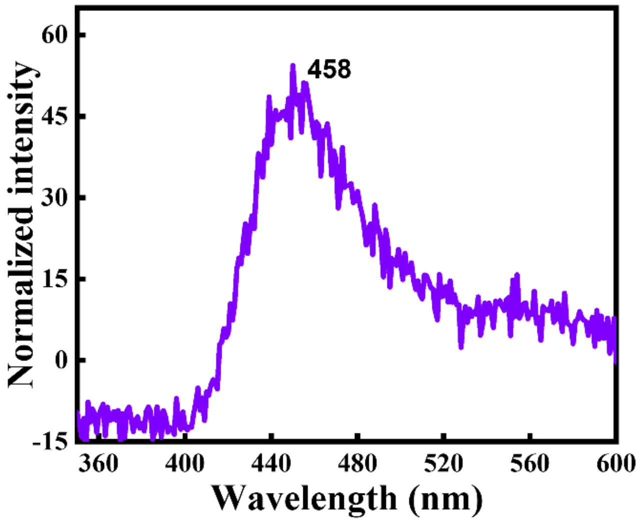

Figure S18. Normalized emission spectra of $[edot]_{0.51}$-$[H]_{0.40}$-HCP.

2.11 TPD test of the catalysts

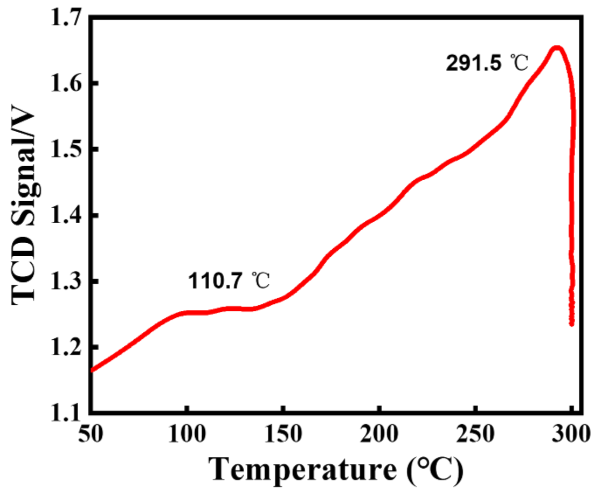

Figure S19. Temperature programmed desorption (TPD) of $[edot]_{0.34}$-$[H]_{0.6}$-HCP.

Table S2. Relative polarities of various solvents, and their Prodan emission maxima (nm).

Entry	Substances	Relative polarity[a]	λ_{max}[b]
1	methanol	0.762[a]	505[b]
2	ethanol	0.654[a]	495[b]
3	1-butanol	0.586[a]	485[b]
4	DMSO	0.444[a]	465[b]
5	dichloromethane	0.309[a]	446
6	HCP	0.298	447
7	$[H]_{0.60}$-HCP	0.298	447
8	$[edot]_{0.34}$-$[H]_{0.60}$-HCP	0.332	453
9	$[edot]_{0.51}$-$[H]_{0.40}$-HCP	0.374	458

[a]: The values of relative polarity were obtained by measuring the shift in the absorption spectrum of Reichardt dye.[4] [b]: the values of λ_{max} were based reported work.[5]

2.12 Condition optimization

Table S3. The optimization of 2-phenylindole from aniline and a-hydroxyacetophenone.

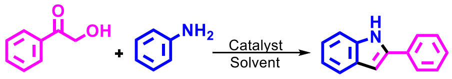

1a 2a 3a

Entry[a]	Catalyst	Solvent	Yield (%)[d]
1	---	Toluene	0
2	$[H]_{0.60}$-HCP	Toluene	28
3	$[H]_{0.60}$-HCP	Dioxane	18
4	$[H]_{0.6}$-HCP	$CHCl_3$	<10
5	$[H]_{0.6}$-HCP	^{i}PrOAc	33

6	$[H]_{0.6}$-HCP	CH_3CN	31
7[b]	$[H]_{0.6}$-HCP	^{i}PrOAc	51
8[c]	$[H]_{0.6}$-HCP	^{i}PrOAc	47

[a]Reaction conditions: **2a** (0.3 mmol), **4a (**0.3 mmol), catalyst (15 mg), solvent (2.0 mL), 60 °C, 5 h; [b]: 80 °C; [c]: 100 °C; [d]: Isolated yield.

Table S4. The optimization of ethyl phenylglycinate from aniline and glyoxal.

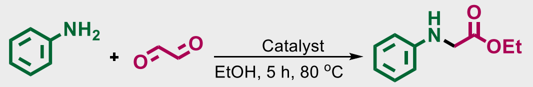

2a **4a** **5a**

Entry[a]	Catalyst	Conv. (%)	Select.(%)	Yield (%)[b]
1	$[edot]_{0.34}$-$[H]_{0.60}$-HCP	>99	58	58
2	$[edot]_{0.34}$-$[H]_{0.40}$-HCP	>99	74	74
3	$[edot]_{0.34}$-$[H]_{0.31}$-HCP	92	53	49
4	$[edot]_{0.51}$-$[H]_{0.40}$-HCP	>99	89	89
5	Amberlyst-15	86	<10	<10
6	HMS-DMSO-SO_3H	97	24	23
7	$[edot]_{0.51}$-HCP	32	0	0
8	$[H]_{0.40}$-HCP	93	33	31

[a]Standard reaction conditions for the competition reaction: **2a** (0.3 mmol), **4a** (0.6 mmol), 10 mol% catalyst (based on the amount of H^+), solvent (2.0 mL); [b]: Isolated yield.

2.13 Proposed mechanism for the formation of 3a and 5a

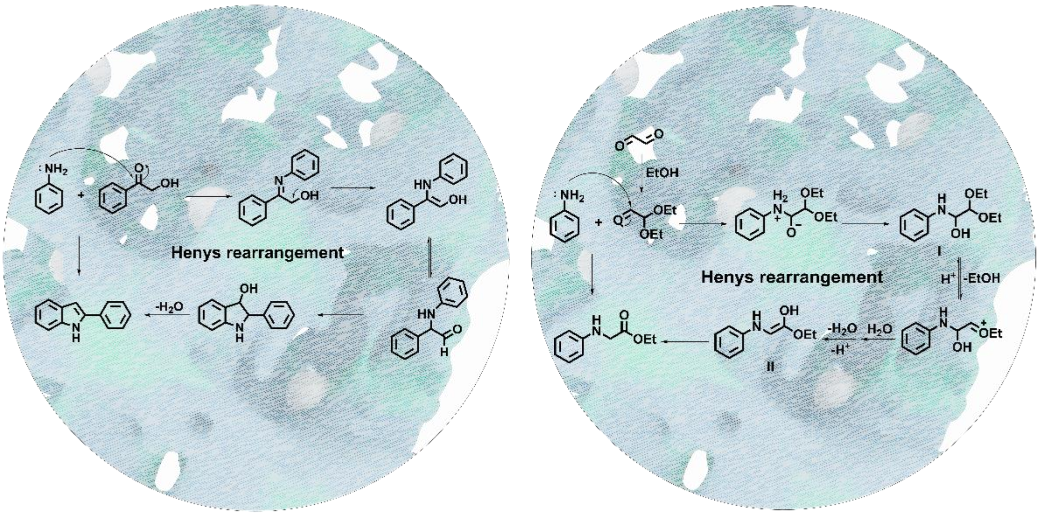

Figure S20. Plausible mechanism for the formation of **3a** and **5a**.[6]

2.14 Simulation data

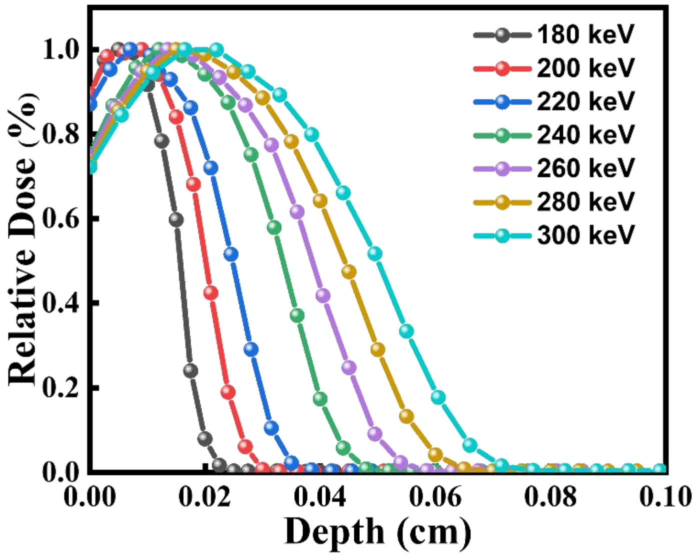

Figure S21. The penetration depth of electron beam radiation in the material (the density of the material is set to 1 g/cm^3).

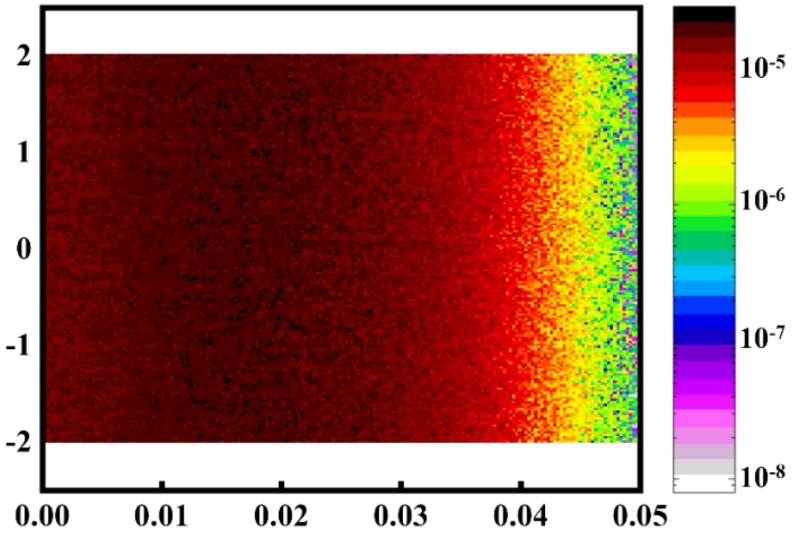

Figure S22. Longitudinal distribution of absorbed dose at the container for the electron beam with average energy of 0.2 MeV.

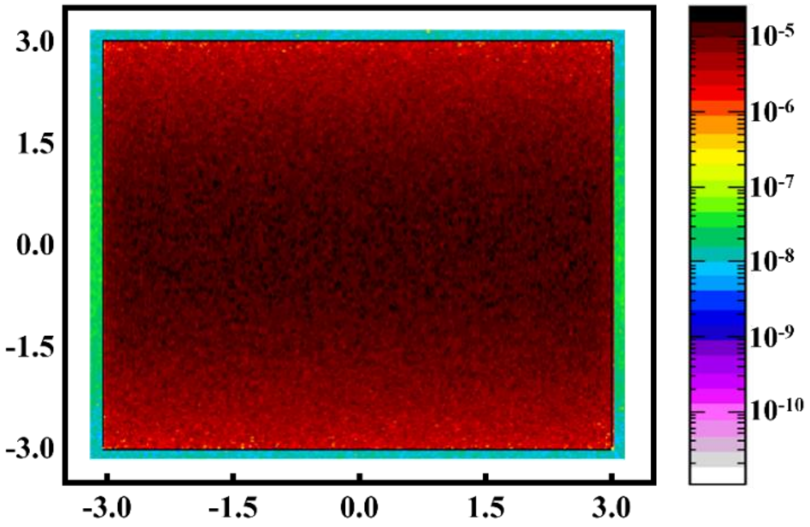

Figure S23. Transverse distribution of absorbed dose at the container for the electron beam with average energy of 0.2 MeV.

The dimensions of the irradiated area are 6 cm in length, 6 cm in width, and 0.3 cm in height. The electron beam with an average energy of 0.2 MeV shows its absorbed dose distribution in the electron absorber at the container, as depicted in **Figures S22-23**. It can be clearly observed that the electron beam is able to penetrate through and distributes evenly, with a maximum penetration depth of 0.04 cm.

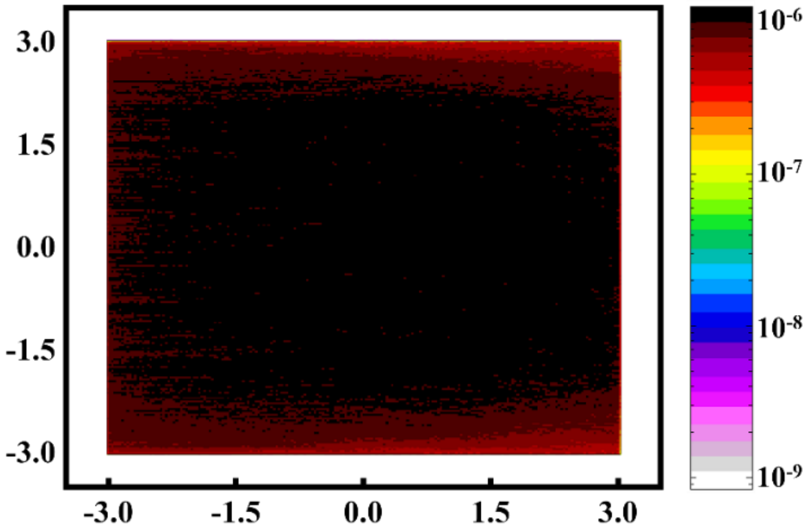

Figure S24. Longitudinal distribution of absorbed dose at the container for the electron beam with average energy of 10 MeV.

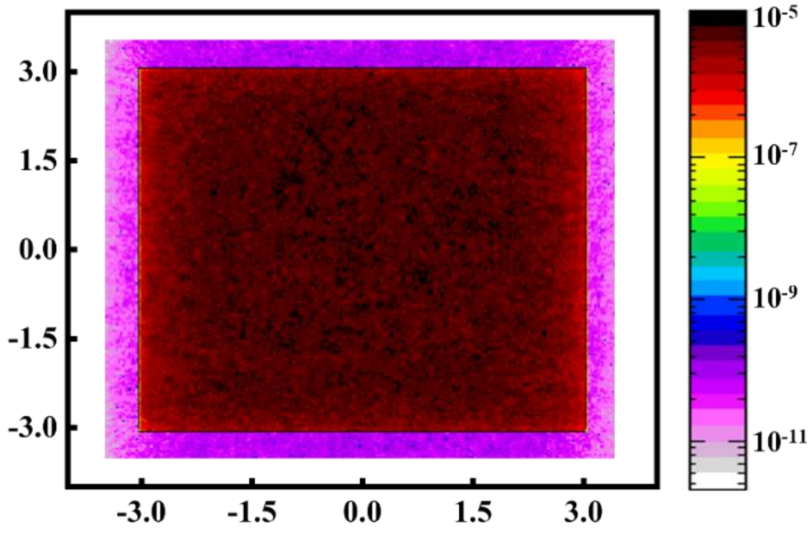

Figure S25. Transverse distribution of absorbed dose at the container for the electron beam with average energy of 10 MeV.

The length, width and height of the square quartz plate are 6 cm, 6 cm and 3 cm, respectively. The distribution of electron absorbed dose at the container for electron beam with average energy of 10 MeV is shown in **Figures S24-25**. It can be clearly seen that the electron beam is able to pass through and is evenly distributed.

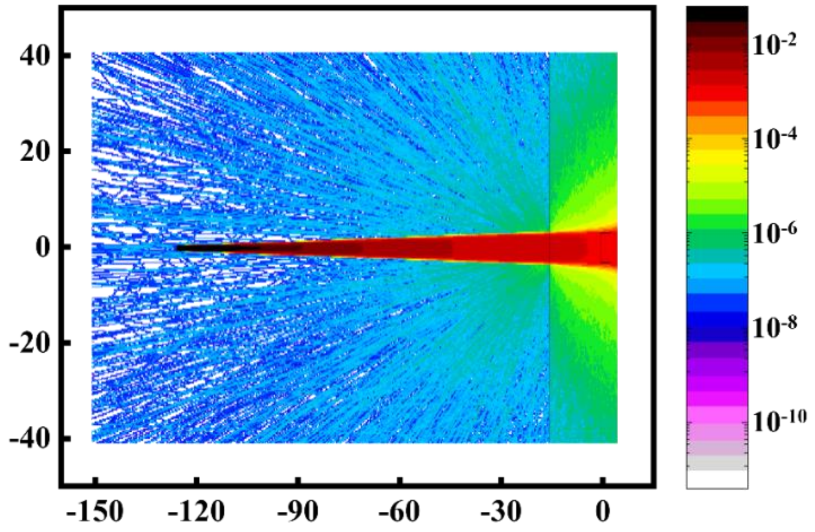

Figure S26. The trajectory of the electron beam during the irradiation process.

2.15 DFT calculations for the two reaction mechanisms

Reaction 1:

Based on the possible reaction 1 path of the above reaction and taking into account the influence of repeating units, we optimized the structure of the polymer, as shown in the figure below:

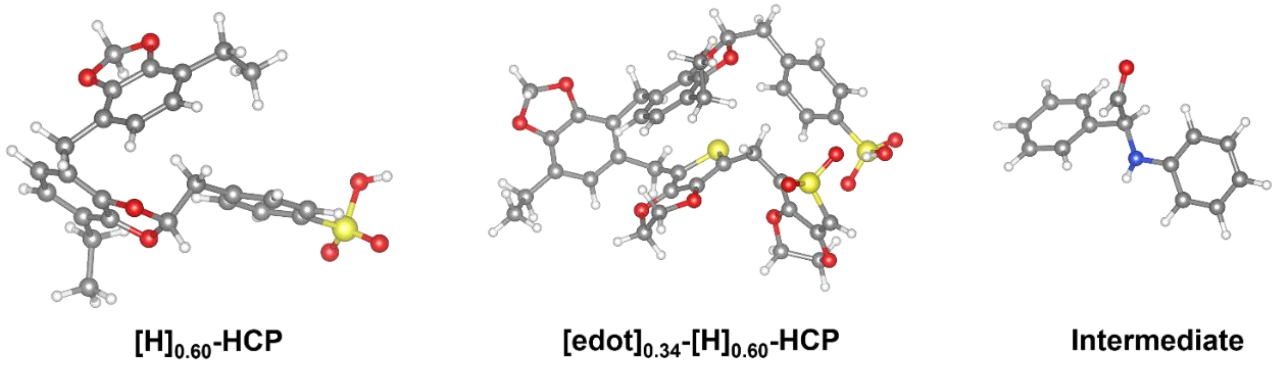

Figure S27. Structural optimization pictures.

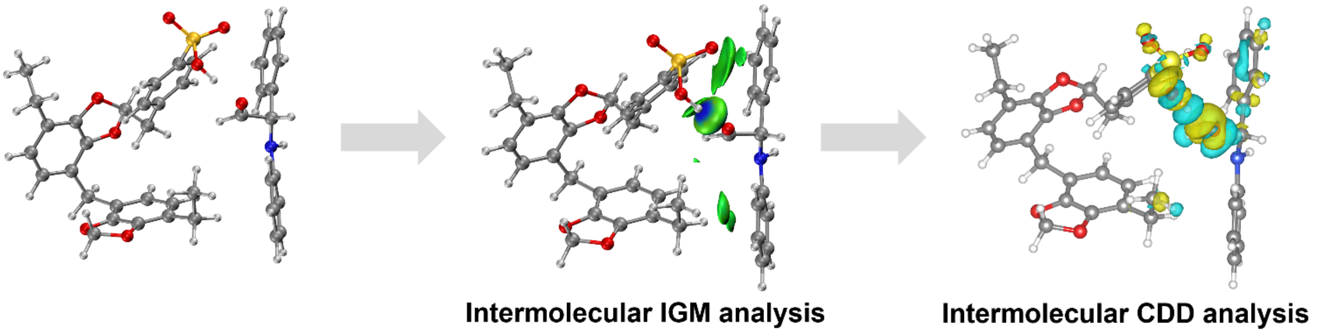

Figure S28. IGM and CDD (Charge Decomposition Density) analysis of intermediate molecules by representative sample $[H]_n$-HCP. The positive and negative phases of the wave function are shown as teal and yellow regions, respectively.

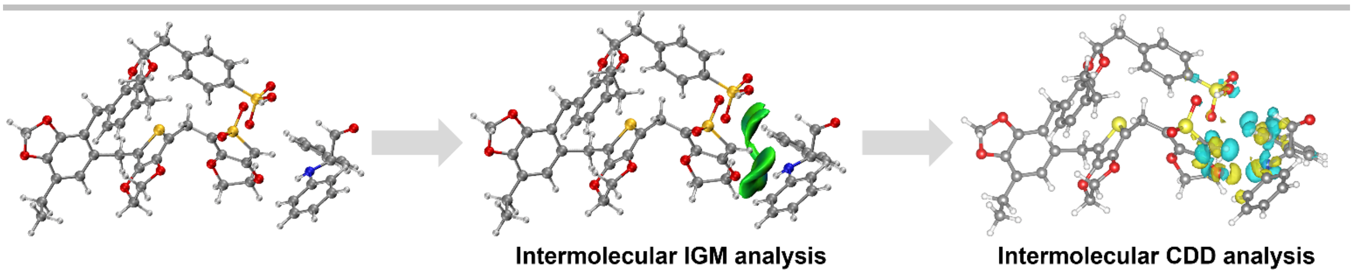

Figure S29. IGM and CDD analysis of intermediate molecules by representative sample [edot]$_m$-[H]$_n$-HCP. The positive and negative phases of the wave function are shown as teal and yellow regions, respectively.

Reaction 2:

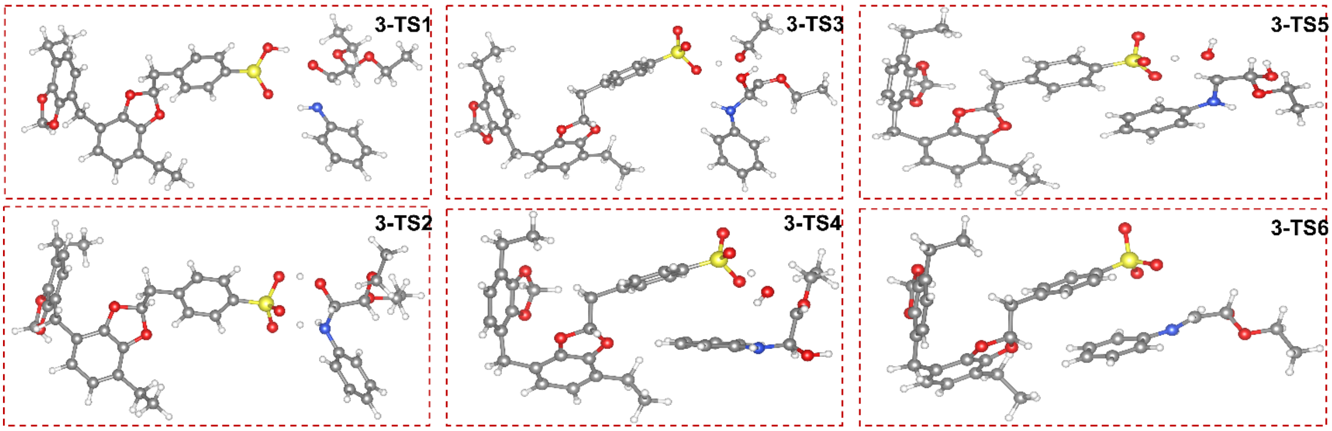

Figure S30. The influence of the representative catalyst [H]$_n$-HCP on the transition state and the configuration diagram.

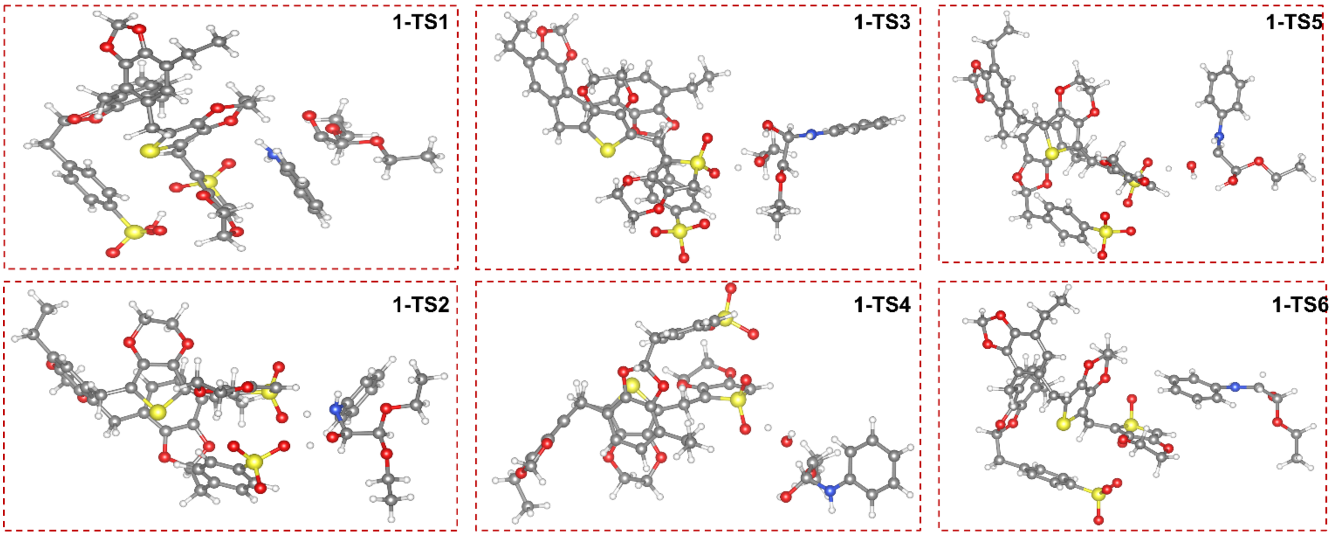

Figure S31. The influence of the representative catalyst [edot]$_m$-[H]$_n$-HCP on the transition state and the configuration diagram..

2.16 Characterization data of all new compounds

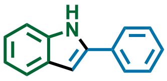

2-Phenyl-*1H*-indole (**3a**): beige solid, yield 94%, 54.4 mg, m.p.: 188–190 °C; **^{1}H NMR** (400 MHz, DMSO-*d*$_6$, 25 °C) δ = 11.58 (s, 1H), 7.88 (d, *J* = 7.5 Hz, 2H), 7.55 (d, *J* = 7.9 Hz, 1H), 7.45 (dt, *J* = 7.9, 3.7 Hz, 3H), 7.31 (t, *J* = 7.4 Hz, 1H), 7.13 (t, *J* = 7.5 Hz, 1H), 7.03 (t, *J* = 7.4 Hz, 1H), 6.90 (s, 1H) ppm; **^{13}C NMR** (101 MHz, DMSO-*d*$_6$, 25 °C) δ = 138.12, 137.65, 132.73, 129.38,

129.16, 127.86, 125.47, 122.07, 120.57, 119.88, 111.81, 99.18 ppm. **R_f Value**: 0.41 (hexanes/dichloromethane (4:1, v/v)). **HRMS**: calcd for $C_{14}H_{12}N^+$, $[M + H]^+$ 194.0964, found 194.0964. Spectral data are in agreement with the literature.[7]

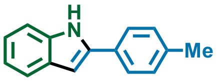

2-(*p*-Tolyl)-*1H*-indole (**3b**): white solid, yield 91%, 56.6 mg, m.p.: 222–224 °C; **^{1}H NMR** (400 MHz, DMSO-d_6, 25 °C) δ = 11.48 (s, 1H), 7.76 (d, J = 8.2 Hz, 2H), 7.51 (d, J = 7.8 Hz, 1H), 7.40 (d, J = 7.9 Hz, 1H), 7.26 (d, J = 8.2 Hz, 2H), 7.09 (t, J = 7.4 Hz, 1H), 6.99 (t, J = 7.4 Hz, 1H), 6.83 (s, 1H), 2.33 (s, 3H) ppm; **^{13}C NMR** (101 MHz, DMSO-d_6, 25 °C) δ = 138.25, 137.48, 137.21, 129.93, 129.17, 125.39, 121.79, 120.34, 119.76, 111.67, 98.51, 21.27 ppm. **R_f Value**: 0.43 (hexanes/dichloromethane (4:1, v/v)). **HRMS**: calcd for $C_{15}H_{14}N^+$, $[M + H]^+$ 208.1121, found 208.1120. Spectral data are in agreement with the literature.[6]

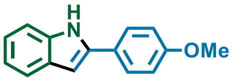

2-(4-Methoxyphenyl)-*1H*-indole (**3c**): white solid, yield 87%, 58.3 mg, m.p.: 226–228 °C; **^{1}H NMR** (400 MHz, DMSO-d_6, 25 °C) δ = 11.43 (s, 1H), 7.80 (d, J = 8.8 Hz, 2H), 7.50 (d, J = 7.6 Hz, 1H), 7.39 (d, J = 7.9 Hz, 1H), 7.12 – 6.95 (m, 4H), 6.75 (s, 1H), 3.80 (s, 3H) ppm; **^{13}C NMR** (101 MHz, DMSO-d_6, 25 °C) δ = 159.26, 138.25, 137.41, 129.33, 126.83, 125.38, 121.51, 120.15, 119.70, 114.81, 111.55, 97.81, 55.64 ppm. **R_f Value**: 0.46 (hexanes/dichloromethane (4:1, v/v)). **HRMS**: calcd for $C_{15}H_{14}NO^+$, $[M + H]^+$ 224.1070, found 224.1070. Spectral data are in agreement with the literature.[6]

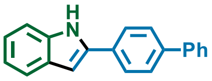

2-([1,1'-Biphenyl]-4-yl)-*1H*-indole (**3d**): white solid, yield 82%, 66.3 mg, m.p.: 296–298 °C; **^{1}H NMR** (400 MHz, DMSO-d_6, 25 °C) δ = 11.62 (s, 1H), 7.97 (d, J = 8.4 Hz, 2H), 7.75 (dd, J = 15.9, 8.0 Hz, 4H), 7.55 (d, J = 7.8 Hz, 1H), 7.47 (q, J = 8.9, 8.2 Hz, 3H), 7.38 (t, J = 7.4 Hz, 1H), 7.13 (t, J = 7.6 Hz, 1H), 7.02 (t, J = 7.5 Hz, 1H), 6.95 (s, 1H) ppm; **^{13}C NMR** (101 MHz, DMSO-d_6, 25 °C) δ = 139.98, 139.32, 137.73, 137.70, 131.76, 129.45, 129.17, 127.98, 127.55, 126.92, 125.95, 122.15, 120.56, 119.91, 111.79, 99.40 ppm. **R_f Value**: 0.51 (hexanes/dichloromethane (5:1, v/v)). **HRMS**: calcd for $C_{20}H_{16}N^+$, $[M + H]^+$ 270.1277, found 270.1276. Spectral data are in agreement with the literature.[6]

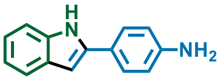

4-(*1H*-Indol-2-yl) aniline (**3e**): yellow solid, yield 68%, 42.5 mg, m.p.: 196–198 °C; **^{1}H NMR** (400 MHz, DMSO-d_6, 25 °C) δ = 11.23 (s, 1H), 7.56 (d, J = 8.5 Hz, 2H), 7.46 (d, J = 7.6 Hz, 1H), 7.36 (d, J = 7.9 Hz, 1H), 6.99 (dt, J = 28.0, 7.4 Hz, 2H), 6.67 (d, J = 8.7 Hz, 2H), 6.60 (s, 1H), 5.31 (s, 2H) ppm; **^{13}C NMR** (101 MHz, DMSO-d_6, 25 °C) δ = 148.89, 139.64, 137.15, 129.58, 126.59, 120.81, 120.46, 119.67, 119.49, 114.51, 111.26, 96.00 ppm. **R_f Value**: 0.38 (hexanes/dichloromethane (1:1, v/v)). **HRMS**: calcd for $C_{14}H_{13}N_2^+$, $[M + H]^+$ 209.1073, found 209.1071. Spectral data are in agreement with the literature.[8]

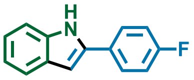

2-(4-Fluorophenyl)-*1H*-indole (**3f**): white solid, yield 79%, 50.1 mg, m.p.: 188–190 °C; **^{1}H NMR** (400 MHz, DMSO-d_6, 25 °C) δ = 11.58 (s, 1H), 7.92 (dd, J = 8.8, 5.4 Hz, 2H), 7.54 (d, J = 7.8 Hz, 1H), 7.44 (d, J = 8.1 Hz, 1H), 7.31 (t, J = 8.8 Hz, 2H), 7.13 (t, J = 7.5 Hz, 1H), 7.02 (t, J = 7.5 Hz, 1H), 6.87 (s, 1H) ppm; **^{13}C NMR** (101 MHz, DMSO-d_6, 25 °C) δ = 163.25, 160.82, 137.62, 137.21, 129.36, 129.14, 127.49, 127.41, 122.06, 120.52, 119.90, 116.38, 116.17, 111.77, 99.14 ppm. **R_f Value**: 0.44 (hexanes/dichloromethane (4:1, v/v)). **HRMS**: calcd for $C_{14}H_{11}FN^+$, $[M + H]^+$ 212.0870, found 212.0869. Spectral data are in agreement with the literature.[9]

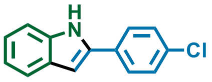

2-(4-Chlorophenyl)-*1H*-indole (**3g**): yellow solid, yield 84%, 57.4 mg, m.p.: 200–202 °C; **^{1}H NMR** (400 MHz, DMSO-d_6, 25 °C) δ = 11.62 (s, 1H), 7.89 (d, J = 8.5 Hz, 2H), 7.53 (t, J = 9.5 Hz, 3H), 7.43 (d, J = 8.1 Hz, 1H), 7.13 (t, J = 7.6 Hz, 1H), 7.02 (t, J = 7.4 Hz, 1H), 6.93 (s, 1H) ppm; **^{13}C NMR** (101 MHz, DMSO-d_6, 25 °C) δ = 137.72, 136.84, 132.26, 131.61, 129.37, 129.03, 127.08, 122.36, 120.67, 120.00, 111.84, 99.81 ppm. **R_f Value**: 0.45 (hexanes/dichloromethane (4:1, v/v)). **HRMS**: calcd for $C_{14}H_{11}ClN^+$, $[M + H]^+$ 228.0575, found 228.0575. Spectral data are in agreement with the literature.[7]

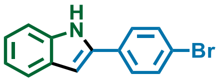

2-(4-Bromophenyl)-*1H*-indole (**3h**): light yellow solid, yield 80%, 65.3 mg, m.p.: 226–228 °C; **^{1}H NMR** (400 MHz, DMSO-d_6, 25 °C) δ = 11.61 (s, 1H), 7.81 (d, J = 8.7 Hz, 2H), 7.64 (d, J = 8.5 Hz, 2H), 7.54 (d, J = 7.9 Hz, 1H), 7.42 (d, J = 8.1 Hz, 1H), 7.13 (t, J = 7.6 Hz, 1H), 7.01 (t, J = 7.4 Hz, 1H), 6.93 (s, 1H) ppm; **^{13}C NMR** (101 MHz, DMSO-d_6, 25 °C) δ = 137.70, 136.84,

132.27, 131.93, 129.00, 127.36, 122.41, 120.78, 120.68, 120.02, 111.86, 99.84 ppm. **R_f Value**: 0.42 (hexanes/dichloromethane (10:1, v/v)). **HRMS**: calcd for $C_{14}H_{11}BrN^+$, $[M + H]^+$ 272.0069, found 272.0068. Spectral data are in agreement with the literature.[7]

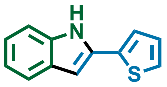

2-(Thiophen-2-yl)-*1H*-indole (3i): white solid, yield 79%, 47.3 mg, m.p.: 160–162 °C; **^{1}H NMR** (400 MHz, DMSO-*d6,* 25 °C) δ = 11.59 (s, 1H), 7.52 (d, *J* = 4.8 Hz, 3H), 7.40 (d, *J* = 8.1 Hz, 1H), 7.19 – 7.08 (m, 2H), 7.01 (t, *J* = 7.3 Hz, 1H), 6.68 (s, 1H) ppm; **^{13}C NMR** (101 MHz, DMSO-*d6,* 25 °C) δ = 137.27, 135.98, 132.86, 128.96, 128.59, 125.57, 123.97, 122.22, 120.38, 120.02, 111.61, 99.22 ppm. **R_f Value**: 0.51 (hexanes/dichloromethane (4:1, v/v)). **HRMS**: calcd for $C_{12}H_{10}NS$, $[M + H]^+$ 200.0528, found 200.0526. Spectral data are in agreement with the literature.[10]

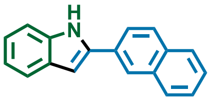

2-(Naphthalen-2-yl)-*1H*-indole (3j): light yellow solid, yield 87%, 63.5 mg, m.p.: 204–206 °C; **^{1}H NMR** (400 MHz, DMSO-*d6,* 25 °C) δ = 11.74 (s, 1H), 8.41 (s, 1H), 8.05 (d, *J* = 8.7 Hz, 1H), 8.01 – 7.90 (m, 3H), 7.61 – 7.45 (m, 4H), 7.15 (t, *J* = 7.5 Hz, 1H), 7.05 (d, *J* = 8.2 Hz, 2H) ppm; **^{13}C NMR** (101 MHz, DMSO-*d6,* 25 °C) δ = 138.04, 137.87, 133.74, 132.76, 130.17, 129.18, 128.89, 128.34, 128.16, 127.18, 126.49, 124.30, 123.28, 122.29, 120.63, 119.93, 111.81, 100.04 ppm. **R_f Value**: 0.46 (hexanes/dichloromethane (4:1, v/v)). **HRMS**: calcd for $C_{18}H_{14}N^+$, $[M + H]^+$ 244.1121, found 244.1121. Spectral data are in agreement with the literature.[6]

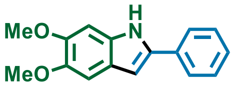

5,6-Dimethoxy-2-phenyl-*1H*-indole (3k): gray solid; yield 93%, 70.7 mg, m.p.: 180–182 °C; **^{1}H NMR** (400 MHz, DMSO-*d6,* 25 °C) δ = 11.24 (s, 1H), 7.77 (d, *J* = 7.6 Hz, 2H), 7.42 (t, *J* = 7.6 Hz, 2H), 7.24 (t, *J* = 7.3 Hz, 1H), 7.03 (s, 1H), 6.91 (s, 1H), 6.75 (d, *J* = 2.4 Hz, 1H), 3.80 (s, 3H), 3.76 (s, 3H) ppm; **^{13}C NMR** (101 MHz, DMSO-*d6,* 25 °C) δ = 147.27, 145.27, 136.40, 133.09, 132.16, 129.30, 127.06, 124.77, 121.97, 102.87, 99.14, 95.36, 56.32, 56.12 ppm. **R_f Value**: 0.39 (hexanes/dichloromethane (4:1, v/v)). **HRMS**: calcd for $C_{16}H_{16}NO_2^+$, $[M + H]^+$ 254.1176; found: 254.1174. Spectral data are in agreement with the literature.[4]

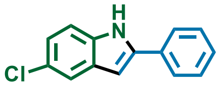

5-Chloro-2-phenyl-*1H*-indole (3l): light yellow solid; yield 74%, 50.5 mg, m.p.: 190–192 °C; **^{1}H NMR** (400 MHz, DMSO-*d6,* 25 °C) δ = 11.78 (s, 1H), 7.86 (d, *J* = 7.3 Hz, 2H), 7.57 (s, 1H), 7.51 – 7.41 (m, 3H), 7.34 (t, *J* = 7.3 Hz, 1H), 7.11 (dd, *J* = 8.6, 2.1 Hz, 1H), 6.88 (s, 1H) ppm; **^{13}C NMR** (101 MHz, DMSO-*d6,* 25 °C) δ = 139.81, 136.04, 132.16, 130.26, 129.44, 128.32, 125.63, 124.40, 121.94, 119.58, 113.24, 98.85 ppm. **R_f Value**: 0.51 (hexanes/dichloromethane (4:1, v/v)). **HRMS**: calcd for $C_{14}H_{11}ClN$, $[M + H]^+$ 228.0575; found: 228.0574. Spectral data are in agreement with the literature.[6]

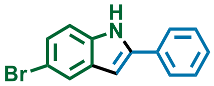

5-Bromo-2-phenyl-*1H*-indole (3m): light yellow solid, yield 73%, 59.6 mg, m.p.: 176–178 °C; **^{1}H NMR** (400 MHz, DMSO-*d6,* 25 °C) δ = 11.78 (s, 1H), 7.86 (d, *J* = 7.5 Hz, 2H), 7.71 (s, 1H), 7.47 (t, *J* = 7.8 Hz, 2H), 7.37 (dd, *J* = 12.0, 7.9 Hz, 2H), 7.22 (dd, *J* = 8.6, 2.0 Hz, 1H), 6.88 (s, 1H) ppm; **^{13}C NMR** (101 MHz, DMSO-*d6,* 25 °C) δ = 139.62, 136.26, 132.10, 130.98, 129.45, 128.35, 125.65, 124.46, 122.61, 113.71, 112.36, 98.73 ppm. **R_f Value**: 0.49 (hexanes/dichloromethane (4:1, v/v). **HRMS**: calcd for $C_{14}H_{11}BrN$, $[M + H]^+$ 272.0069; found: 272.0068. Spectral data are in agreement with the literature.[11]

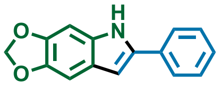

6-Phenyl-*5H*-[1,3] dioxolo[4,5-f] indole (3n): white solid; yield 82%, 58.4 mg, m.p.: 236–238 °C; **^{1}H NMR** (400 MHz, DMSO-*d6,* 25 °C) δ =11.39 (s, 1H), 7.77 (d, *J* = 7.9 Hz, 2H), 7.41 (t, *J* = 7.7 Hz, 2H), 7.25 (t, *J* = 7.3 Hz, 1H), 7.00 (s, 1H), 6.93 (s, 1H), 6.76 (s, 1H), 5.96 (s, 2H) ppm; **^{13}C NMR** (101 MHz, DMSO-*d6,* 25 °C) δ = 144.75, 142.88, 136.67, 132.92, 132.60, 129.30, 127.13, 124.72, 122.98, 100.63, 99.60, 98.98, 92.50 ppm; **R_f Value**: 0.42 (hexanes/dichloromethane (2:1, v/v). **HRMS**: calcd for $C_{15}H_{12}NO_2^+$, $[M + H]^+$ 238.0863; found: 238.0862. Spectral data are in agreement with the literature.[12]

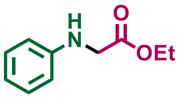

Ethyl phenylglycinate (5a): white solid, yield 89%, 47.9 mg, m.p.: 53–55 °C; **^{1}H NMR** (400 MHz, Chloroform-*d*, 25 °C) δ = 7.19 (t, *J* = 7.9 Hz, 2H), 6.75 (t, *J* = 7.3 Hz, 1H), 6.60 (d, *J* = 8.1 Hz, 2H), 4.29 (s, 1H), 4.23 (q, *J* = 7.1 Hz, 2H), 3.88 (d, *J* = 4.8

Hz, 2H), 1.29 (t, *J* = 7.1 Hz, 3H) ppm; **^{13}C NMR** (101 MHz, Chloroform-*d*, 25 °C) *δ* = 171.18, 147.08, 129.34, 118.20, 113.03, 61.34, 45.88, 14.23 ppm; **R_f Value**: 0.62 (heptane /ethyl acetate (5:1, v/v) + 0.5 vol.-% NEt_3). **HRMS**: calcd for $C_{10}H_{14}NO_2^+$, $[M + H]^+$ 180.1019; found: 180.1019. Spectral data are in agreement with the literature.[13]

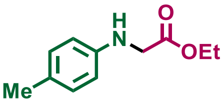

Ethyl *p*-tolylglycinate (**5b**): colorless solid, yield 83%, 48.1 mg, m.p.: 42–44 °C; **^{1}H NMR** (400 MHz, Chloroform-*d*, 25 °C) *δ* = 7.27 (d, *J* = 8.4 Hz, 1H), 6.98 (d, *J* = 8.2 Hz, 1H), 6.43 (d, *J* = 8.2 Hz, 1H), 4.80 (s, 1H), 4.25 (q, *J* = 7.1 Hz, 2H), 3.92 (d, *J* = 5.6 Hz, 2H), 2.22 (s, 3H), 1.30 (t, *J* = 7.1 Hz, 3H) ppm; **^{13}C NMR** (101 MHz, Chloroform-*d*, 25 °C) *δ* = 170.63, 141.77, 132.99, 129.00, 128.25, 111.36, 109.93, 61.42, 46.03, 20.04, 14.20 ppm. **R_f Value**: 0.68 (heptane /ethyl acetate (5:1, v/v) + 0.5 vol.-% NEt_3). **HRMS** (ESI, TOF): *m/z*: calcd for $C_{11}H_{16}NO_2^+$, $[M + H]^+$ 194.1176; found: 194.1176. An explanation for the reduced integration of one hydrogen atom in the phenyl region is that the hydrogen atom is replaced by deuterium (D). Spectral data are in agreement with the literature.[12]

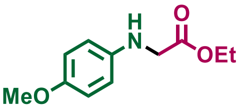

Ethyl (4-methoxyphenyl)glycinate (**5c**): yellow solid, yield 87%, 54.6 mg, m.p.: 46–48 °C; **^{1}H NMR** (400 MHz, Chloroform-*d*, 25 °C) *δ* = 6.79 (d, *J* = 8.9 Hz, 2H), 6.59 (d, *J* = 8.9 Hz, 2H), 4.23 (q, *J* = 7.2 Hz, 2H), 3.86 (s, 2H), 3.74 (s, 3H), 1.29 (t, *J* = 7.1 Hz, 3H) ppm; **^{13}C NMR** (101 MHz, Chloroform-*d*, 25 °C) *δ* = 171.42, 152.65, 141.30, 114.92, 114.41, 61.24, 55.75, 46.85, 14.21 ppm. **R_f Value**: 0.58 (heptane /ethyl acetate (1:1, v/v) + 0.5 vol.-% NEt_3). **HRMS**: calcd for $C_{11}H_{16}NO_3^+$, $[M + H]^+$ 210.1125; found: 210.1123. Spectral data are in agreement with the literature.[12]

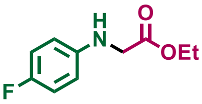

Ethyl (4-fluorophenyl)glycinate (**5d**): light yellow solid, yield 76%, 45.0 mg, m.p.: 48–50 °C; **^{1}H NMR** (400 MHz, Chloroform-*d*, 25 °C) *δ* = 6.90 (t, *J* = 8.7 Hz, 2H), 6.54 (dd, *J* = 9.0, 4.3 Hz, 2H), 4.24 (q, *J* = 7.1 Hz, 2H), 4.19 (s, 1H), 3.86 (d, *J* = 5.6 Hz, 2H), 1.29 (t, *J* = 7.2 Hz, 3H) ppm; **^{13}C NMR** (101 MHz, Chloroform-*d*, 25 °C) *δ* = 171.11, 157.42, 155.07, 143.43, 115.89, 115.66, 113.94, 61.37, 46.45, 14.20 ppm; **R_f Value**: 0.38 (heptane /ethyl acetate (5:1, v/v) + 0.5 vol.-% NEt_3). **HRMS**: calcd for $C_{10}H_{13}FNO_2^+$, $[M + H]^+$ 198.0925; found: 198.0925. Spectral data are in agreement with the literature.[12]

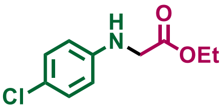

Ethyl (4-chlorophenyl)glycinate (**5e**): colorless solid, yield 73%, 46.8 mg, m.p.: 90–92 °C; **^{1}H NMR** (400 MHz, Chloroform-*d*, 25 °C) *δ* = 7.13 (d, *J* = 8.8 Hz, 2H), 6.52 (d, *J* = 8.8 Hz, 2H), 4.31 (s, 1H), 4.24 (q, *J* = 7.1 Hz, 2H), 3.86 (d, *J* = 5.4 Hz, 2H), 1.30 (t, *J* = 7.1 Hz, 3H) ppm; **^{13}C NMR** (101 MHz, Chloroform-*d*, 25 °C) *δ* = 170.85, 145.61, 129.17, 122.83, 114.07, 61.47, 45.86, 14.20 ppm; **R_f Value**: 0.42 (heptane /ethyl acetate (5:1, v/v) + 0.5 vol.-% NEt_3). **HRMS**: calcd for $C_{10}H_{13}ClNO_2^+$, $[M + H]^+$ 214.0629; found: 214.0628. Spectral data are in agreement with the literature.[12]

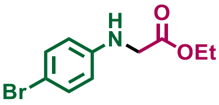

Ethyl (4-bromophenyl)glycinate (**5f**): colorless solid, yield 75%, 58.1 mg, m.p.: 98–100 °C; **^{1}H NMR** (400 MHz, Chloroform-*d*, 25 °C) *δ* = 7.21 (d, *J* = 8.8 Hz, 2H), 6.51 (d, *J* = 8.8 Hz, 2H), 6.24 (s, 1H), 4.11 (q, *J* = 7.2 Hz, 2H), 3.88 (d, *J* = 6.2 Hz, 2H), 1.19 (t, *J* = 7.1 Hz, 3H) ppm; **^{13}C NMR** (101 MHz, Chloroform-*d*, 25 °C) *δ* = 171.46, 147.92, 131.80, 114.57, 107.44, 60.81, 45.01, 14.59 ppm. **R_f Value**: 0.51 (heptane /ethyl acetate (5:1, v/v) + 0.5 vol.-% NEt_3). **HRMS**: calcd for $C_{10}H_{13}BrNO_2^+$, $[M + H]^+$ 258.0124; found: 258.0124.[12]

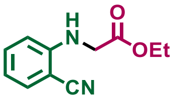

Ethyl (2-cyanophenyl)glycinate (**5g**): white solid, yield 71%,43.5 mg, m.p.: 95–97 °C; **^{1}H NMR** (400 MHz, Chloroform-*d*, 25 °C) *δ* = 7.41 (q, *J* = 7.6 Hz, 2H), 6.75 (t, *J* = 7.5 Hz, 1H), 6.55 (d, *J* = 8.4 Hz, 1H), 5.16 (s, 1H), 4.27 (q, *J* = 7.1 Hz,

2H), 3.99 (d, J = 5.6 Hz, 2H), 1.31 (t, J = 7.1 Hz, 3H) ppm; **^{13}C NMR** (101 MHz, Chloroform-d, 25 °C) δ = 169.72, 149.12, 134.28, 132.99, 117.55, 110.83, 96.72, 61.71, 45.04, 14.18 ppm; **R_f Value**: 0.32 (heptane /ethyl acetate (2:1, v/v) + 0.5 vol.-% NEt_3). **HRMS**: calcd for $C_{11}H_{13}N_2O_2^+$, [M + H]$^+$ 205.0972; found: 205.0972.[14]

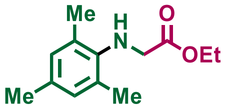

Ethyl (3,5-dimethylphenyl) glycinate (5h): yellow solid, yield 64%,42.5 mg, m.p.: 205–207 °C; **^{1}H NMR** (400 MHz, DMSO-d_6, 25 °C) δ = 6.70 (s, 2H), 4.20 (s, 1H), 4.06 (q, J = 7.0 Hz, 2H), 3.73 (s, 2H), 2.20 (s, 6H), 2.13 (s, 3H) , 1.15 (t, J = 7.1 Hz, 3H) ppm; **^{13}C NMR** (101 MHz, DMSO-d_6, 25 °C) δ = 172.45, 143.47, 130.01, 129.56, 128.81, 60.76, 49.76, 20.56, 18.75, 14.41 ppm; **R_f Value**: 0.56 (heptane /ethyl acetate (4:1, v/v) + 0.5 vol.-% NEt_3). **HRMS**: calcd for $C_{13}H_{20}NO_2^+$, [M + H]$^+$ 222.1416; found: 222.1489.[15]

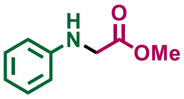

Methyl phenylglycinate (5i): yellow solid, yield 85%, 42.1 mg, m.p.: 40–42 °C; **^{1}H NMR** (400 MHz, DMSO-d_6, 25 °C) δ = 7.07 (t, J = 7.9 Hz, 2H), 6.57 (d, J = 7.2 Hz, 1H), 6.54 (d, J = 7.5 Hz, 2H), 5.99 (s, 1H), 3.89 (d, J = 6.2 Hz, 2H), 3.64 (s, 3H) ppm; **^{13}C NMR** (101 MHz, DMSO-d_6, 25 °C) δ = 172.33, 148.52, 129.33, 116.79, 112.54, 52.03, 44.98 ppm; **R_f Value**: 0.42 (heptane /ethyl acetate (4:1, v/v) + 0.5 vol.-% NEt_3). **HRMS**: calcd for $C_9H_{12}NO_2^+$, [M + H]$^+$ 166.0863; found: 166.0863.

2.17 ^{1}H NMR and ^{13}C NMR spectra (3a)

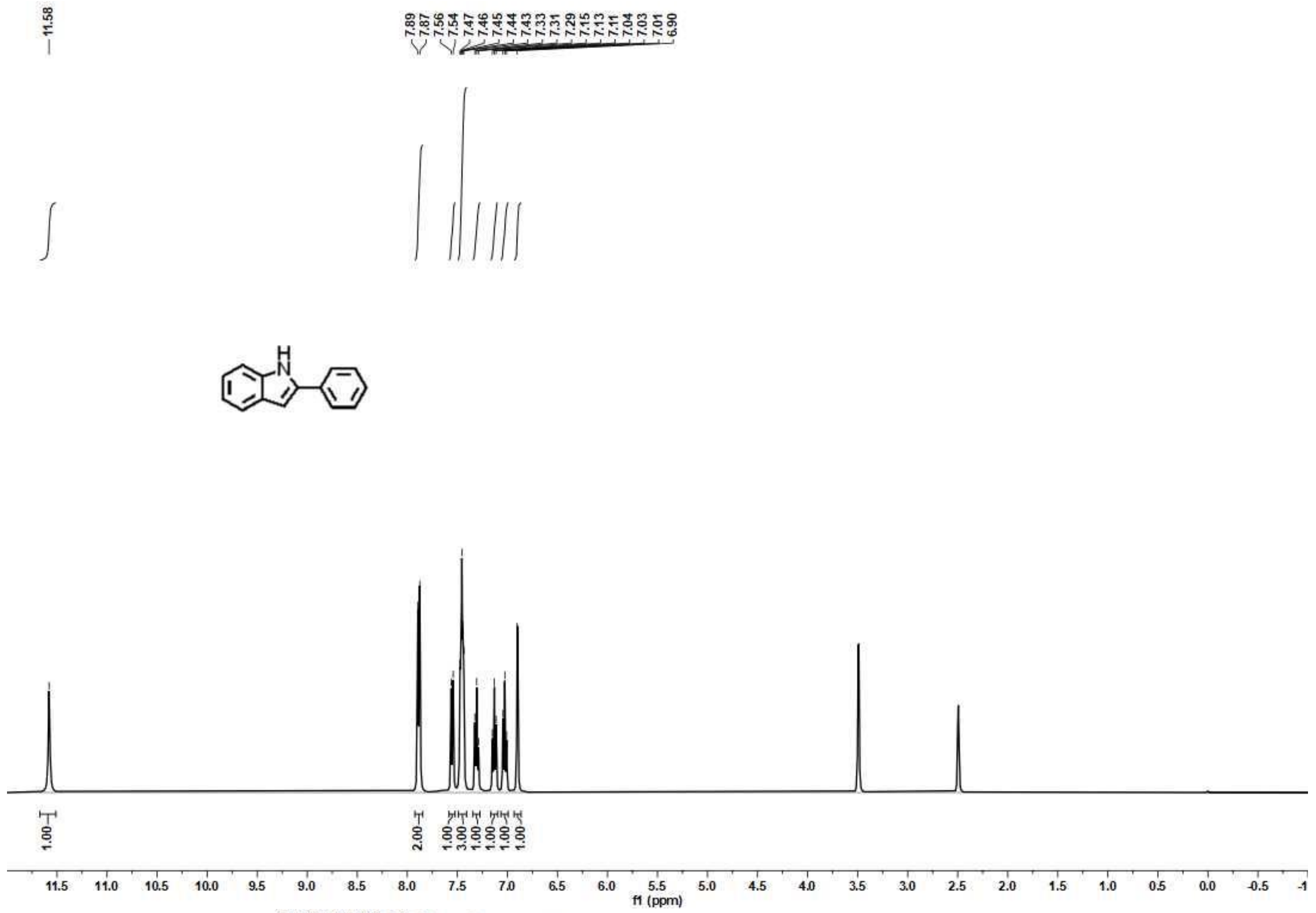

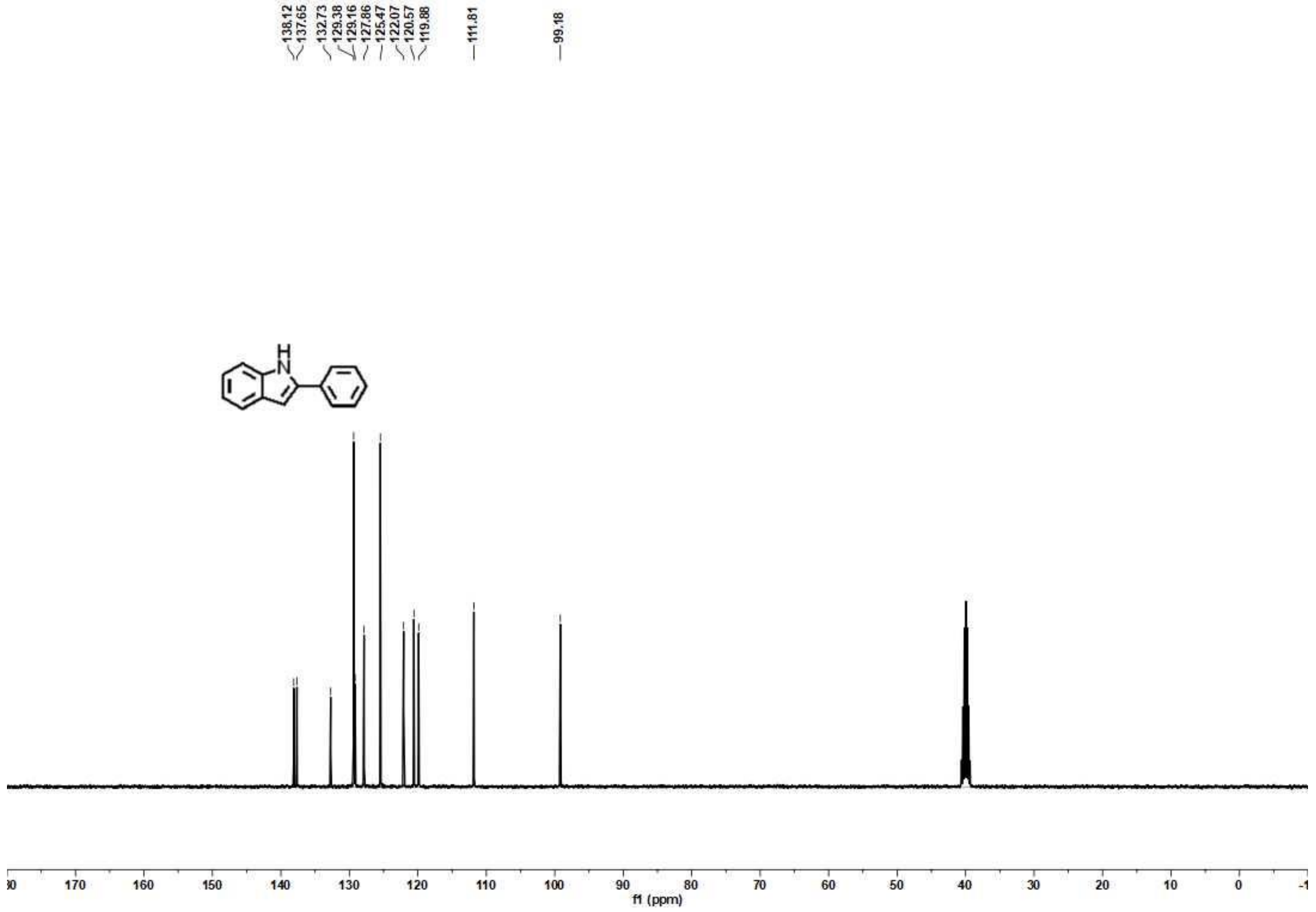

3b

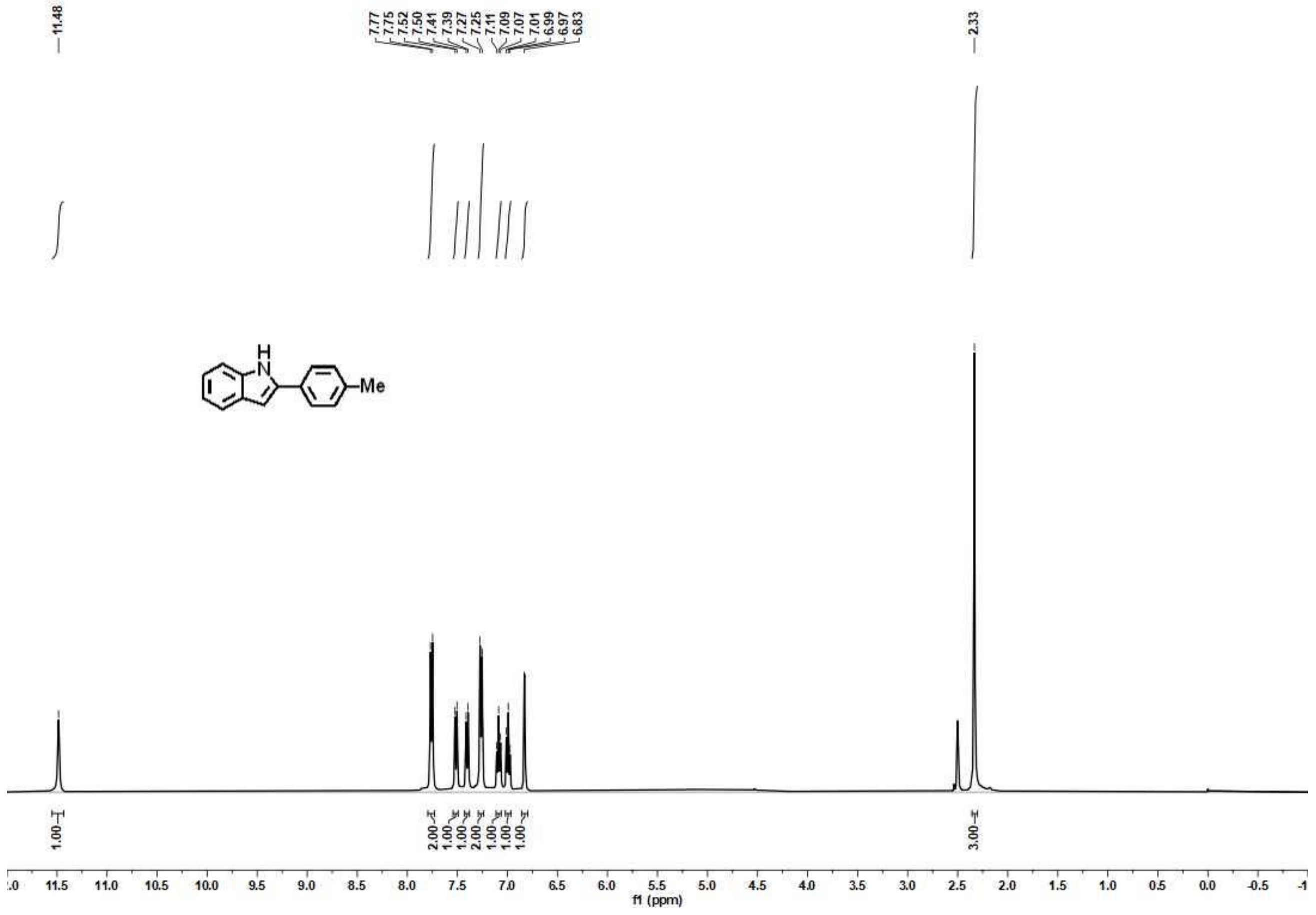

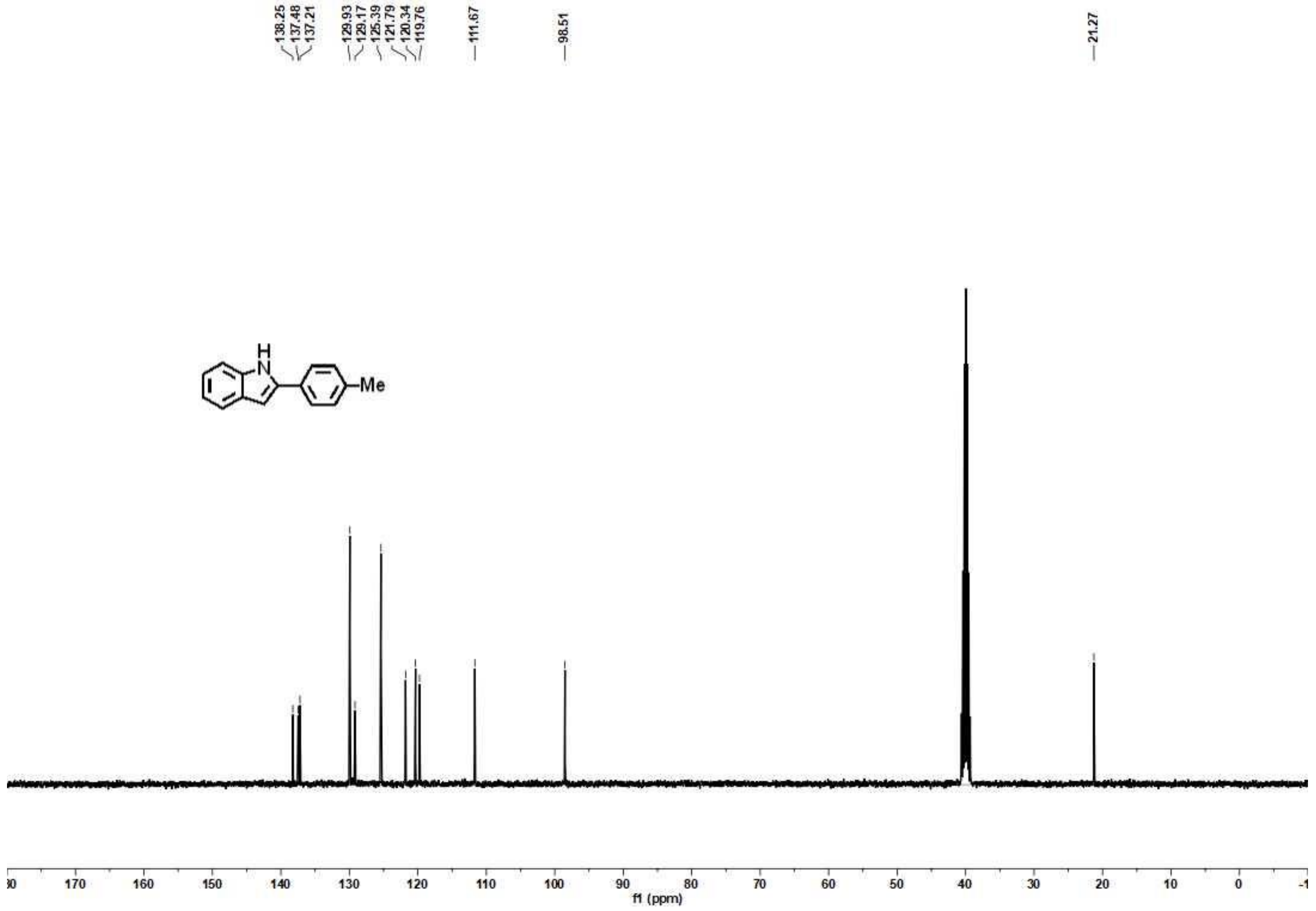

3c

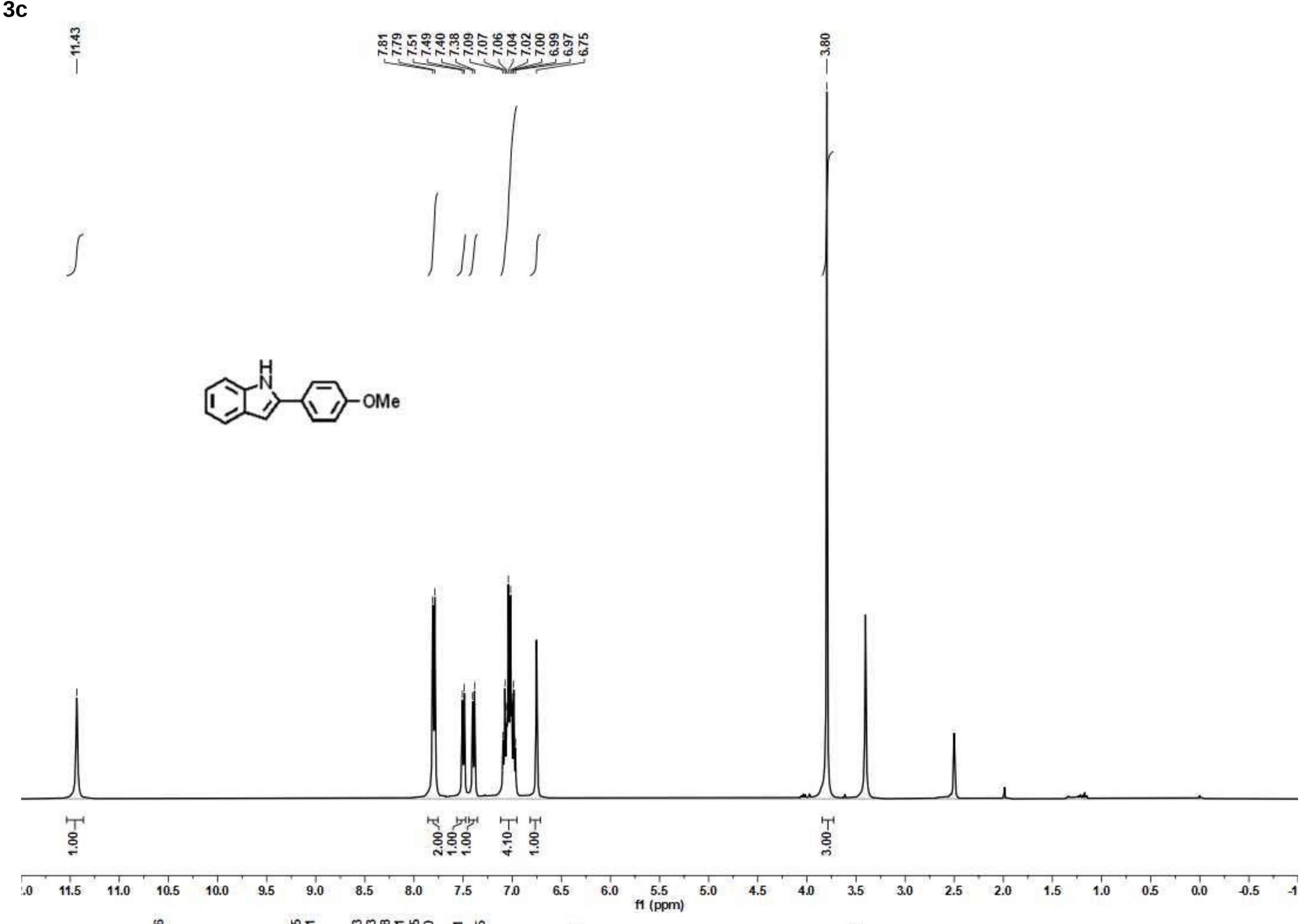

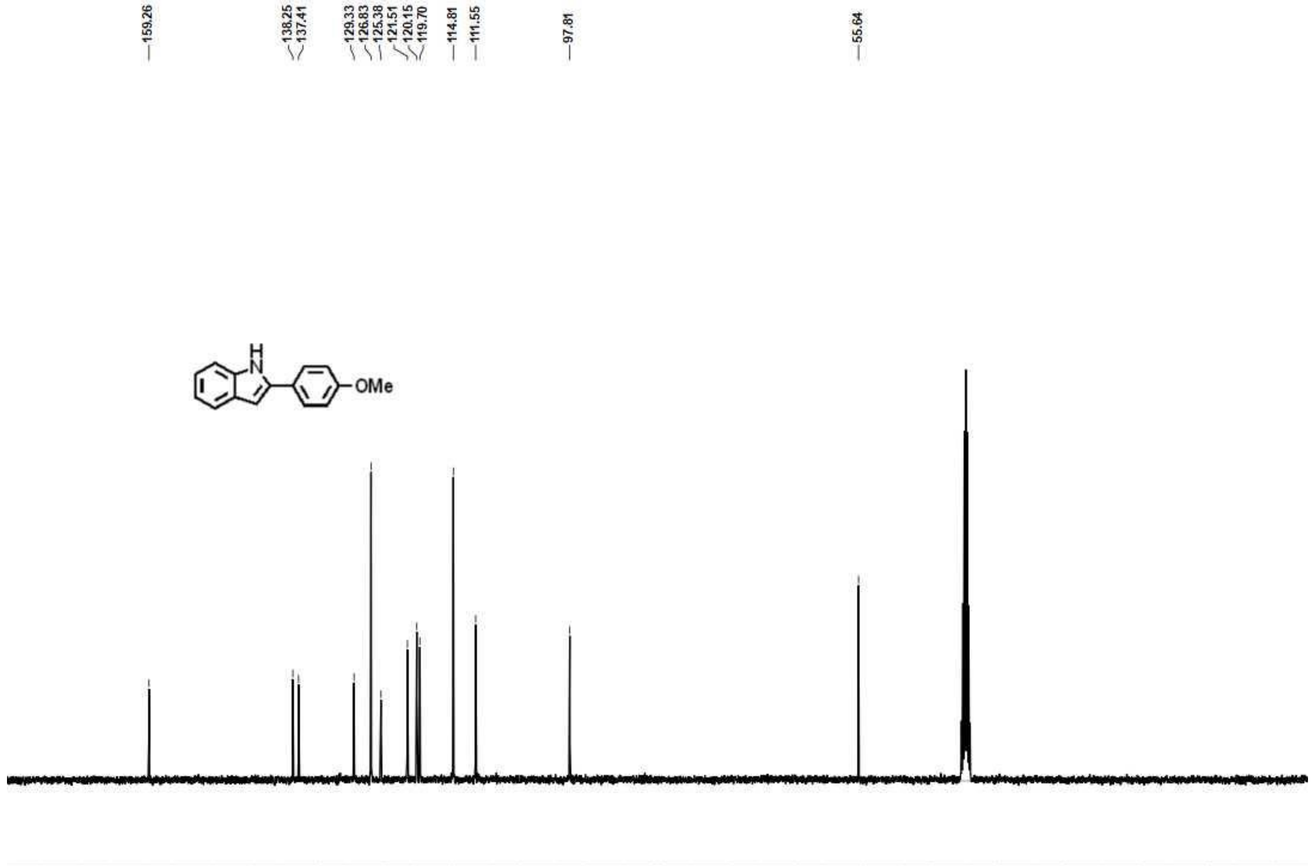

3d

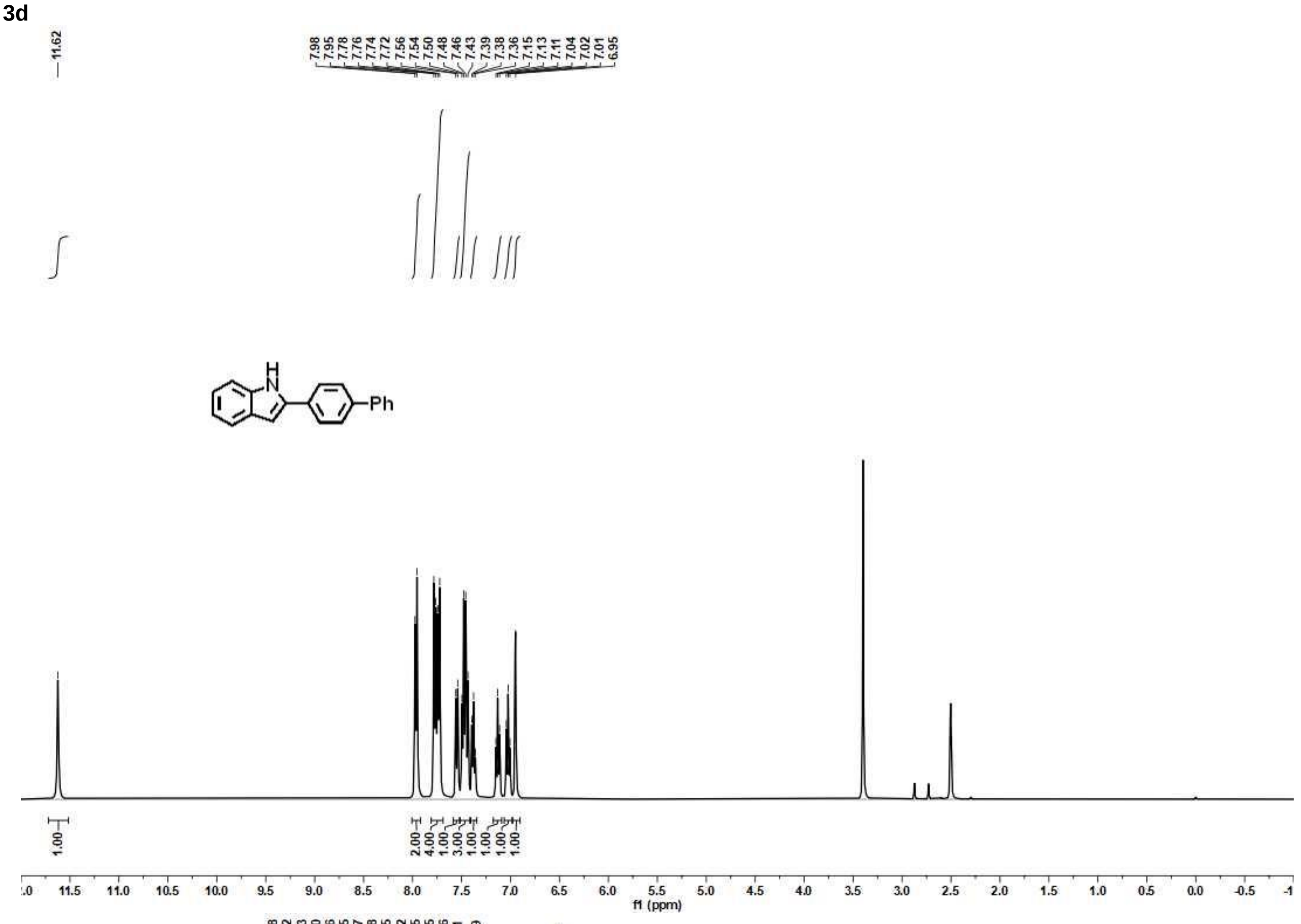
11.62
7.98
7.95
7.78
7.76
7.74
7.72
7.56
7.54
7.50
7.48
7.46
7.43
7.39
7.38
7.36
7.15
7.13
7.11
7.04
7.02
7.01
6.95
Ph
1.00
2.00
4.00
1.00
3.00
1.00
1.00
1.00
1.00
f1 (ppm)

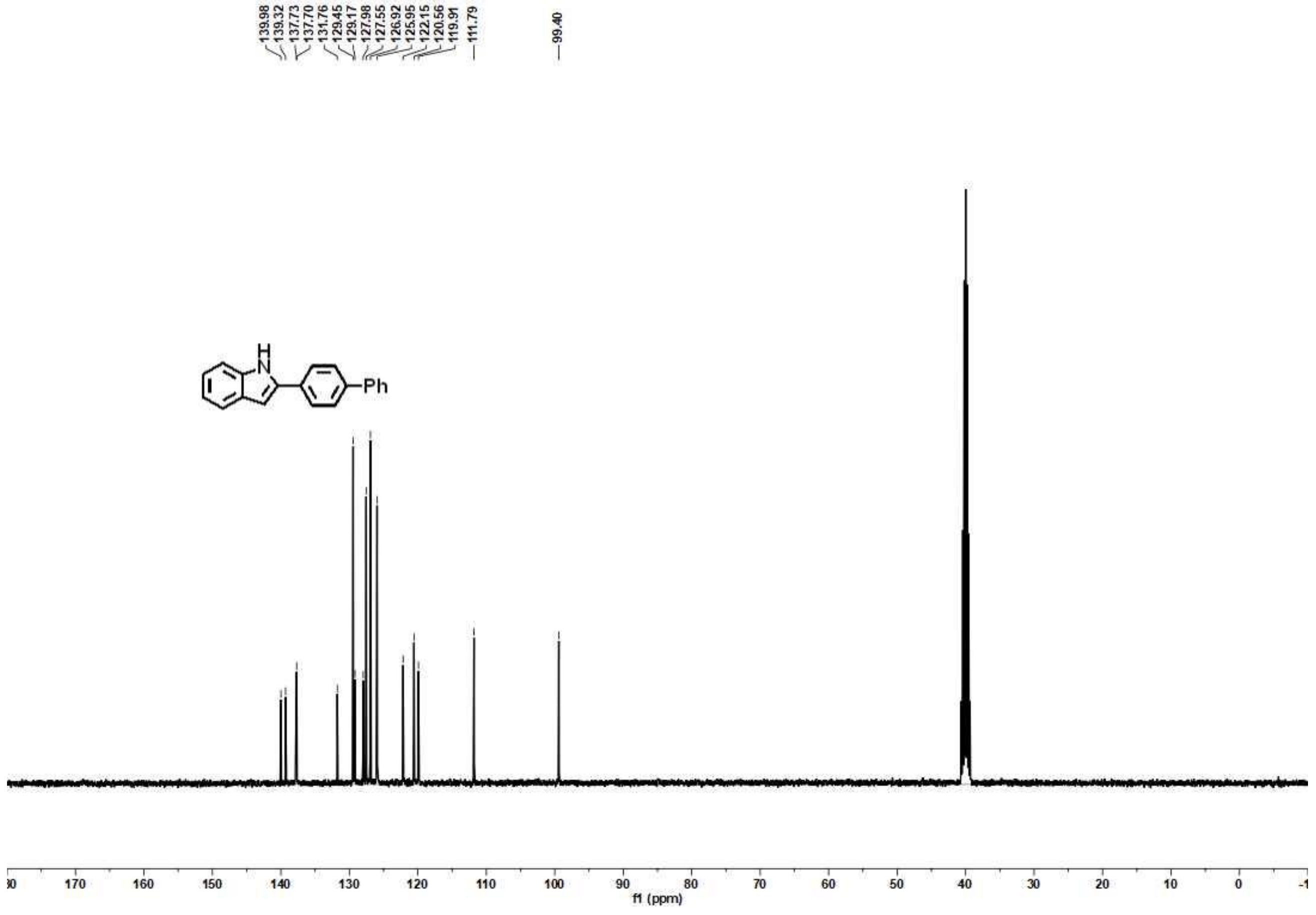
139.98
139.32
137.73
137.70
131.76
129.45
129.17
127.98
127.55
126.92
125.95
122.15
120.56
119.91
111.79
99.40
Ph
f1 (ppm)

3e

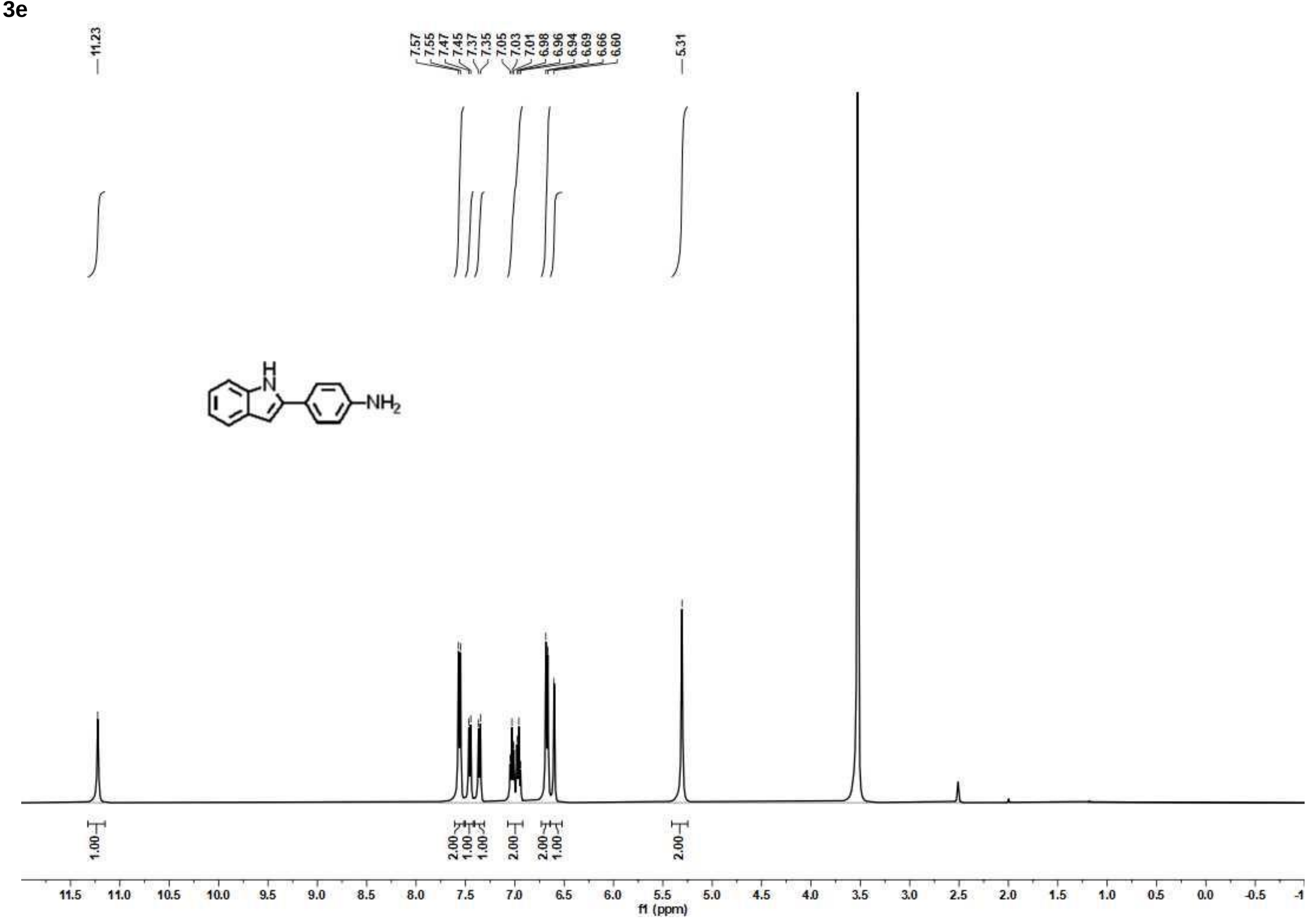
11.23
7.57
7.55
7.47
7.45
7.37
7.35
7.05
7.03
7.01
6.98
6.96
6.94
6.69
6.66
6.60
5.31
NH2
1.00
2.00
1.00
1.00
2.00
2.00
1.00
2.00
f1 (ppm)

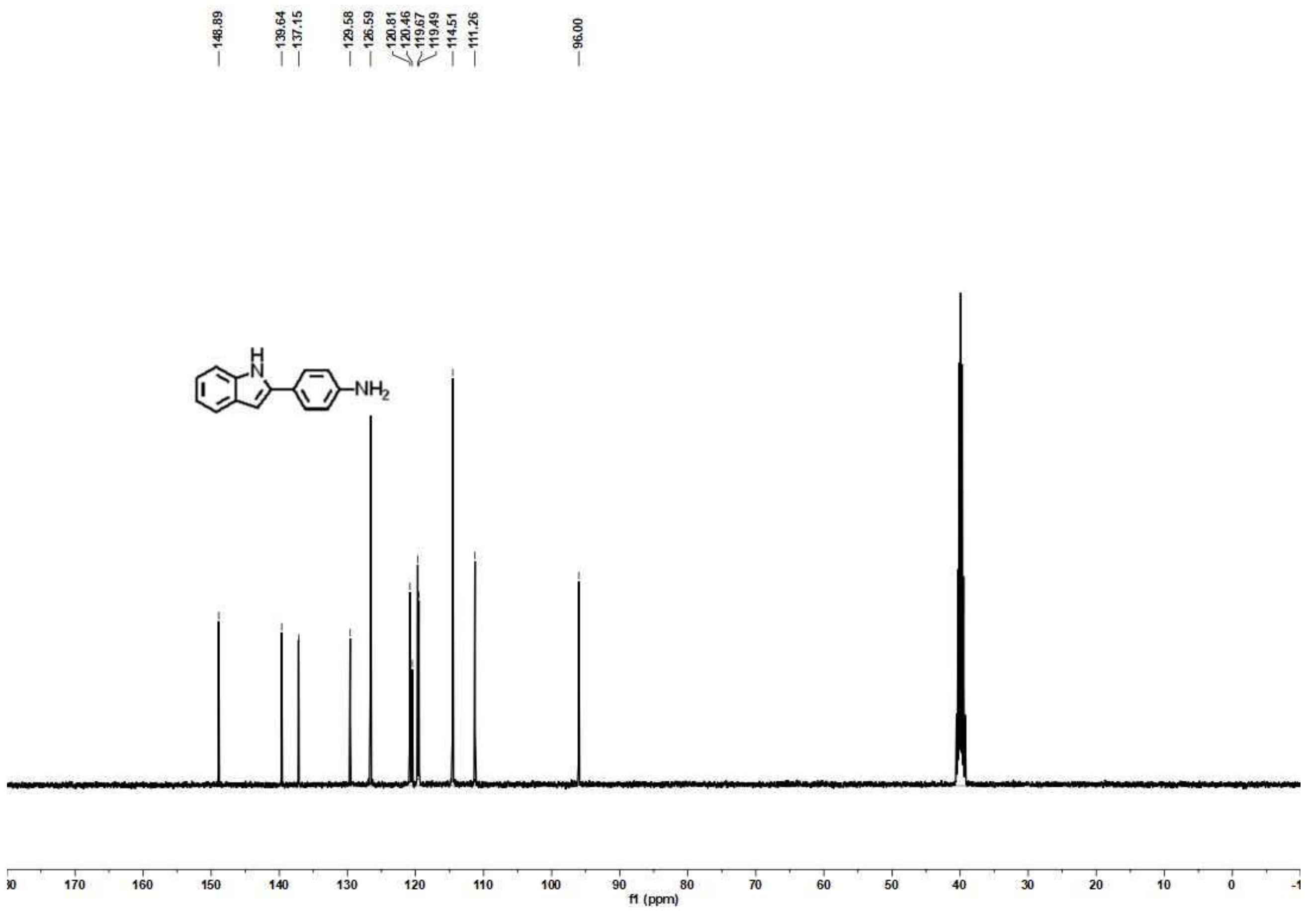
148.89
139.64
137.15
129.58
126.59
120.81
120.46
119.67
119.49
114.51
111.26
96.00
NH2
f1 (ppm)

3f

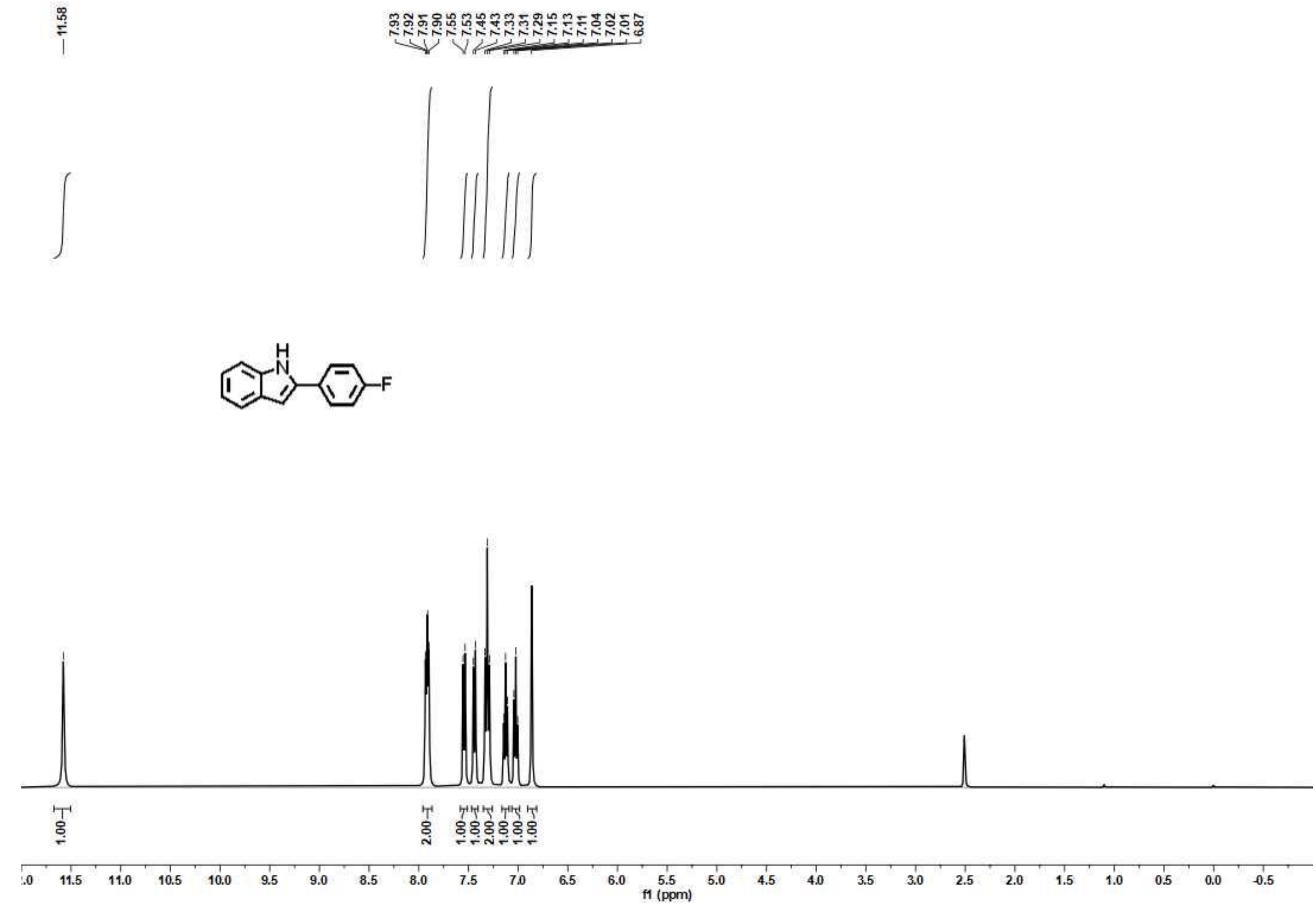

11.58
7.93
7.92
7.91
7.90
7.55
7.53
7.45
7.43
7.33
7.31
7.29
7.15
7.13
7.11
7.04
7.02
7.01
6.87
1.00
2.00
1.00
1.00
2.00
1.00
1.00
1.00
f1 (ppm)

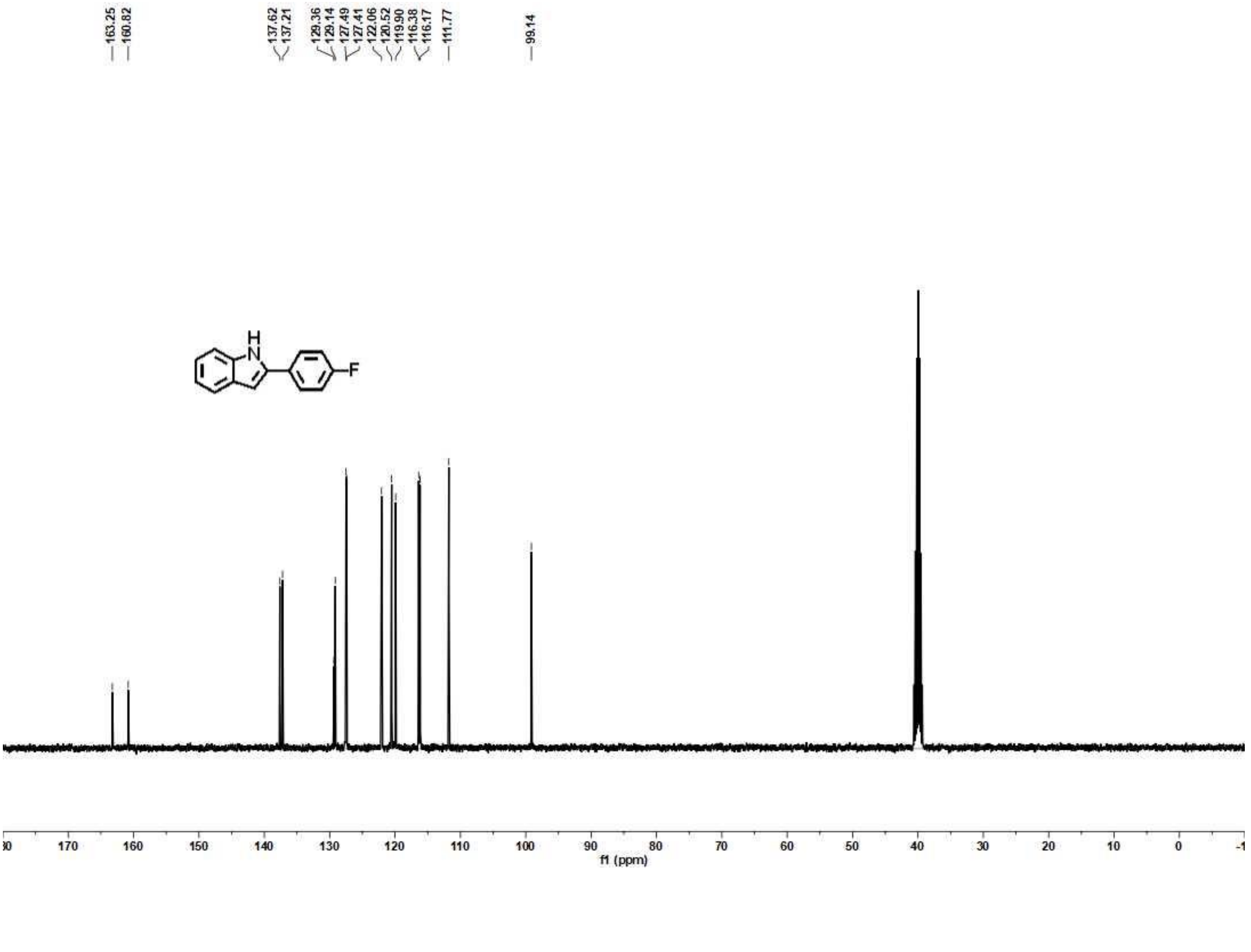

163.25
160.82
137.62
137.21
129.36
129.14
127.49
127.41
122.06
120.52
119.90
116.38
116.17
111.77
99.14
f1 (ppm)

3g

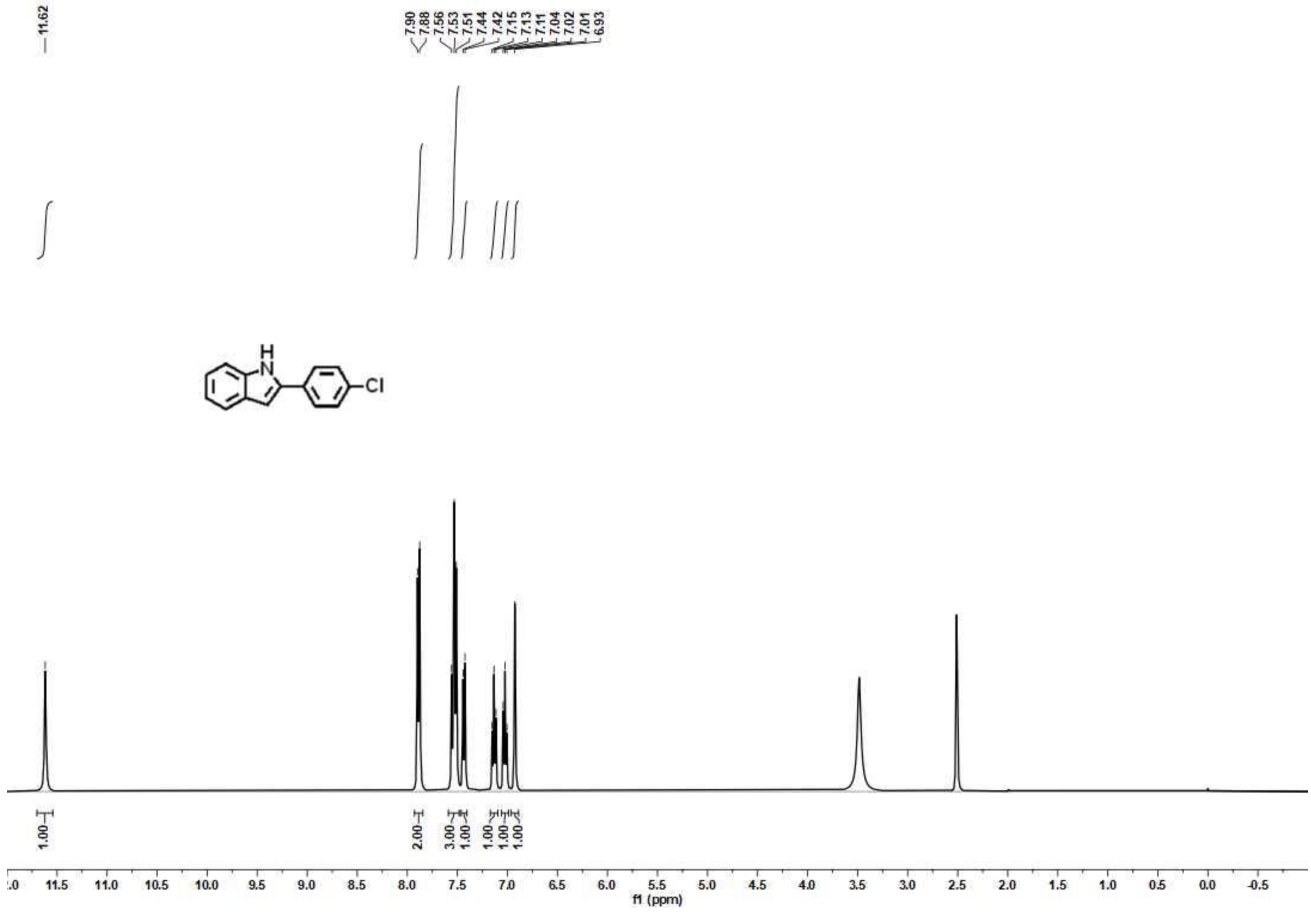

11.62
7.90
7.88
7.56
7.53
7.51
7.44
7.42
7.15
7.13
7.11
7.04
7.02
7.01
6.93
1.00
2.00
3.00
1.00
1.00
1.00
1.00
f1 (ppm)

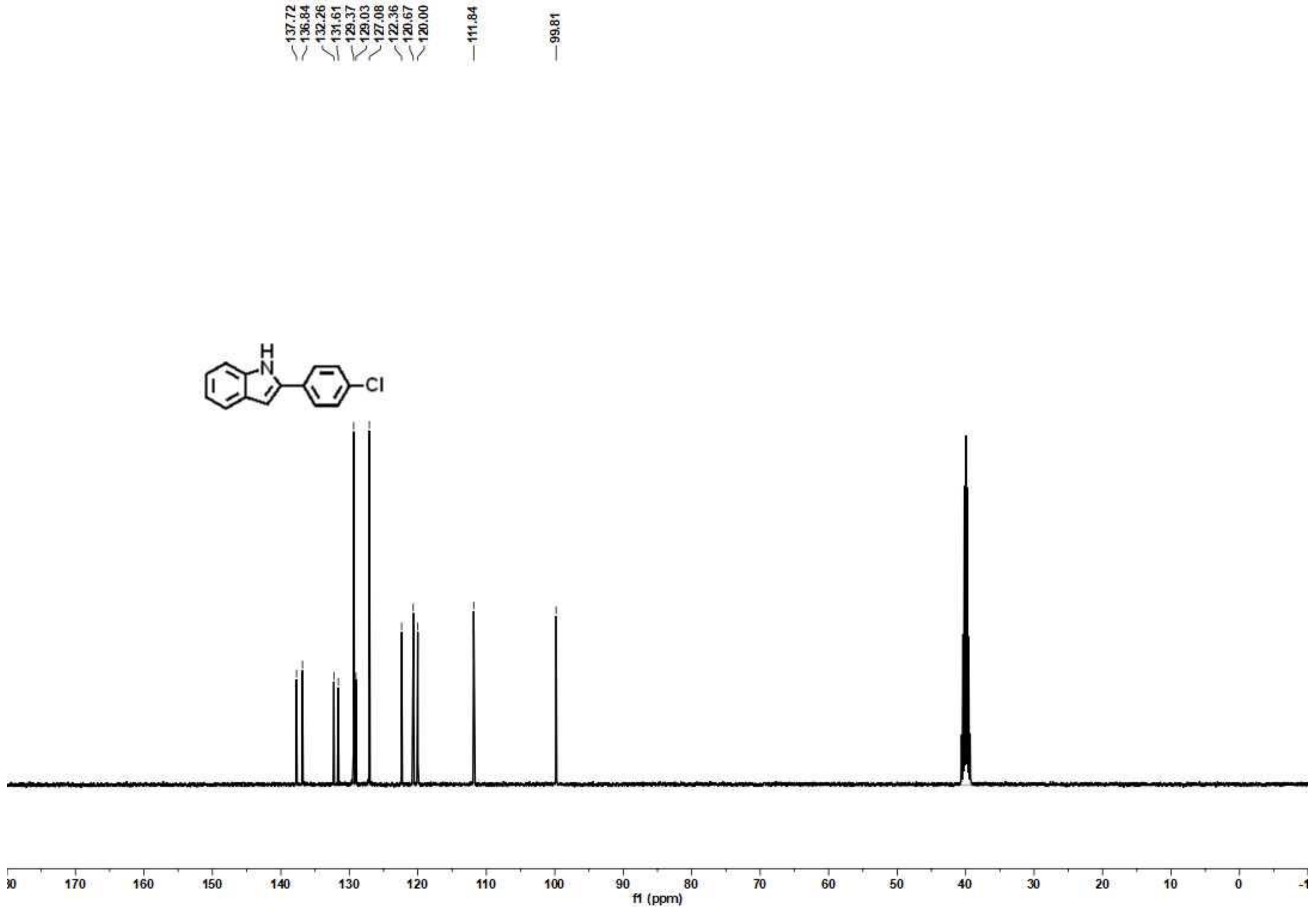

137.72
136.84
132.26
131.61
129.37
129.03
127.08
122.36
120.67
120.00
111.84
99.81
f1 (ppm)

3h

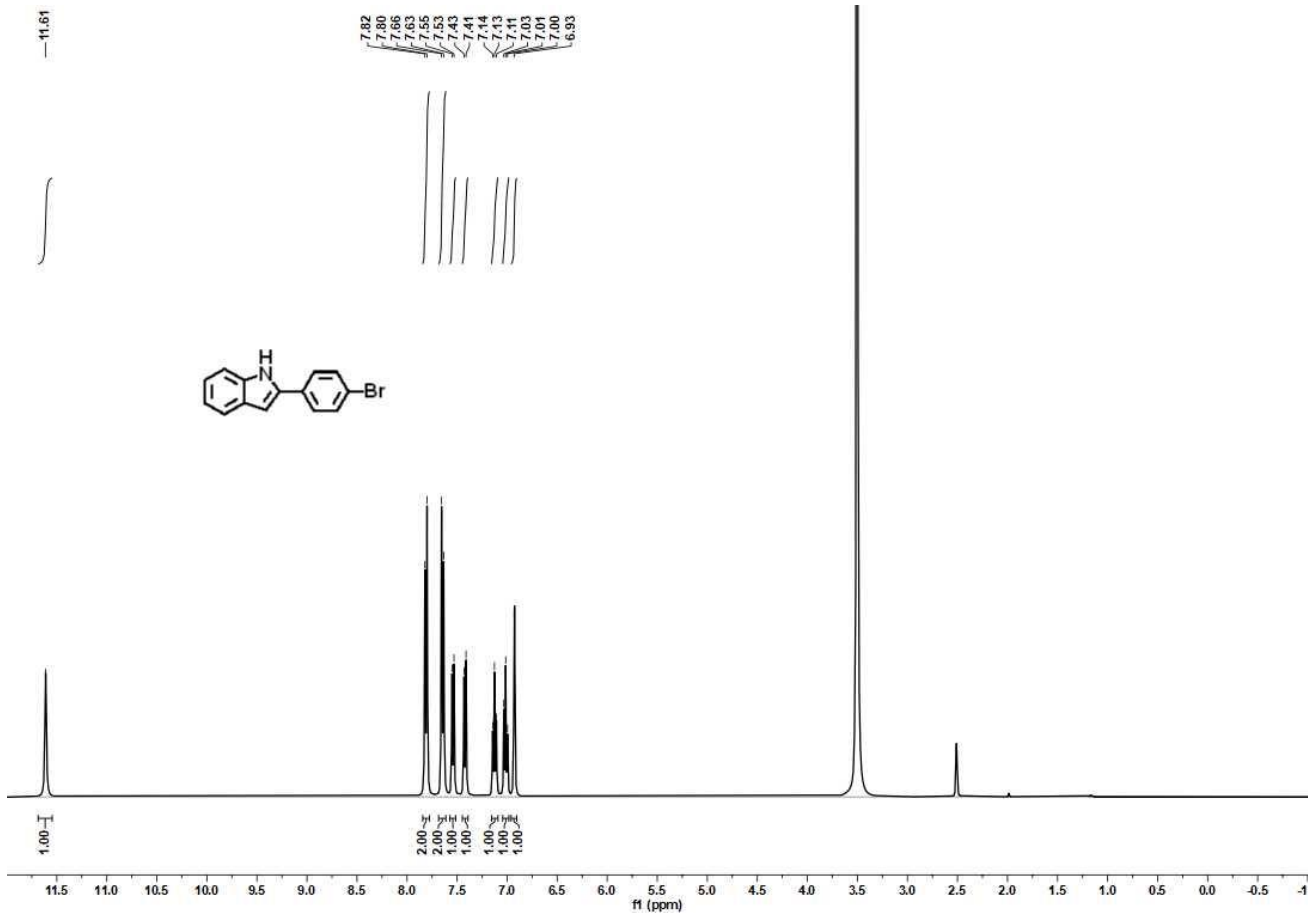
11.61
7.82
7.80
7.66
7.63
7.55
7.53
7.43
7.41
7.14
7.13
7.11
7.03
7.01
7.00
6.93
Br
H
N
1.00
2.00
2.00
1.00
1.00
1.00
1.00
1.00
f1 (ppm)

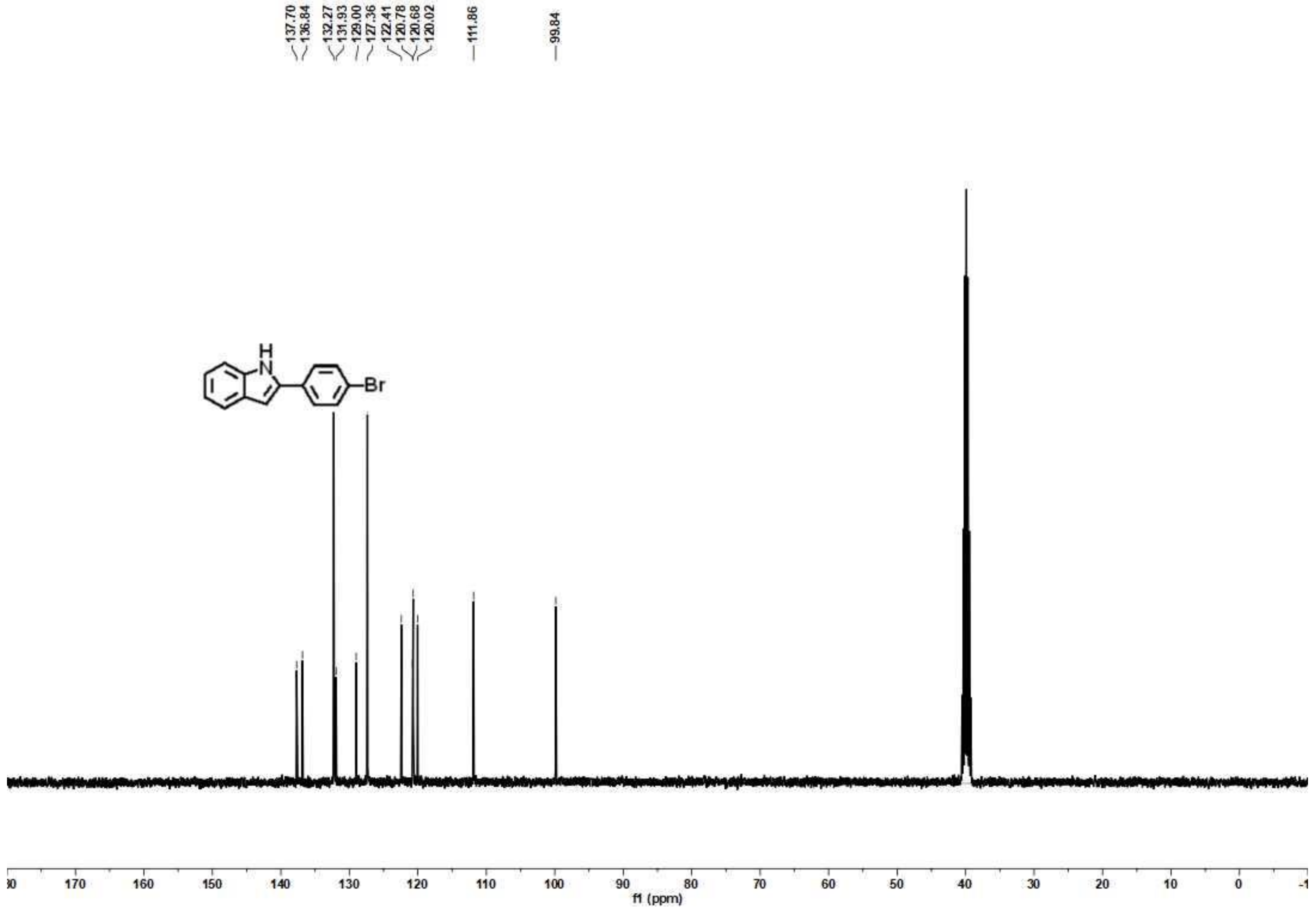
137.70
136.84
132.27
131.93
129.00
127.36
122.41
120.78
120.68
120.02
111.86
99.84
Br
H
N
f1 (ppm)

3i

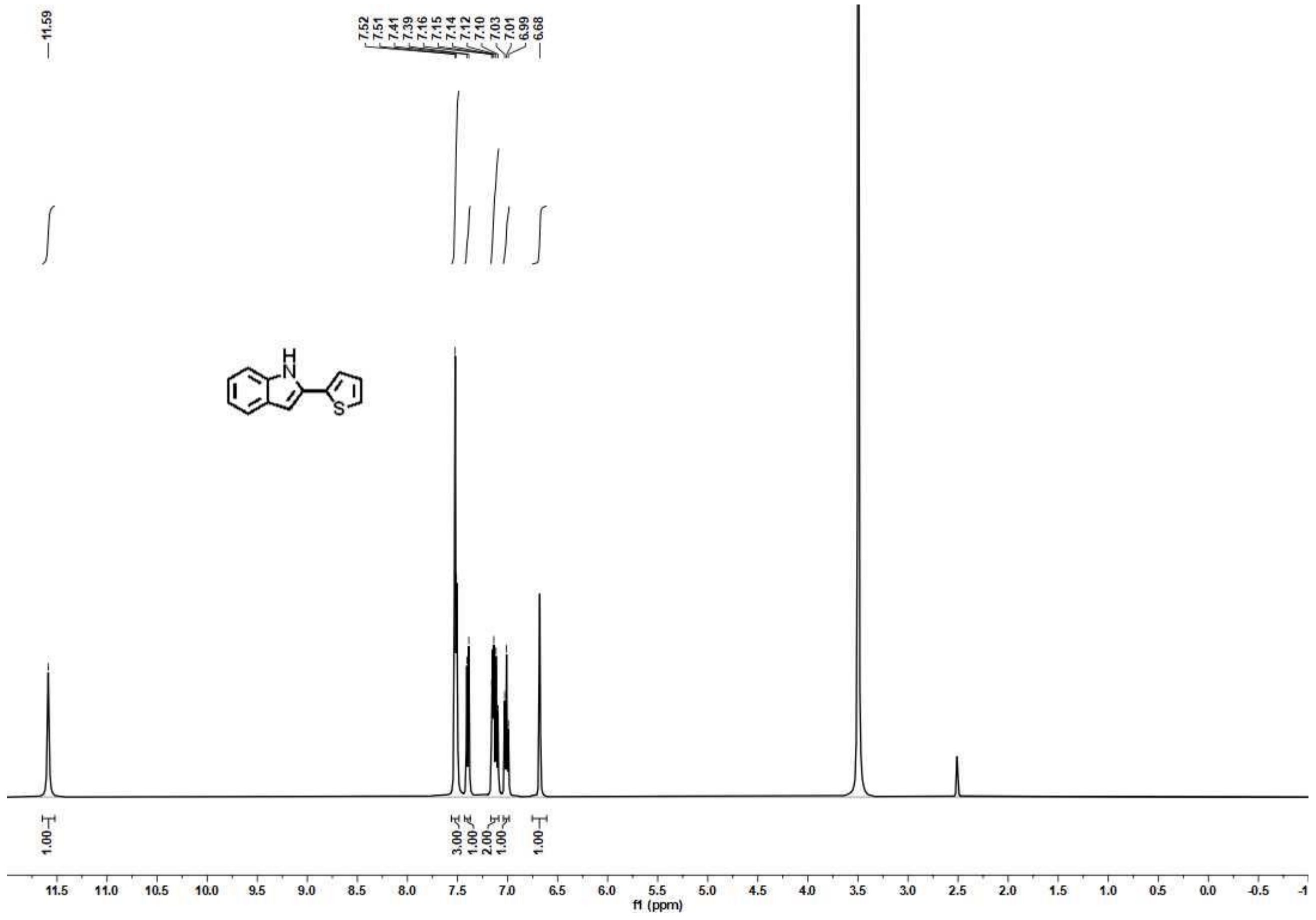

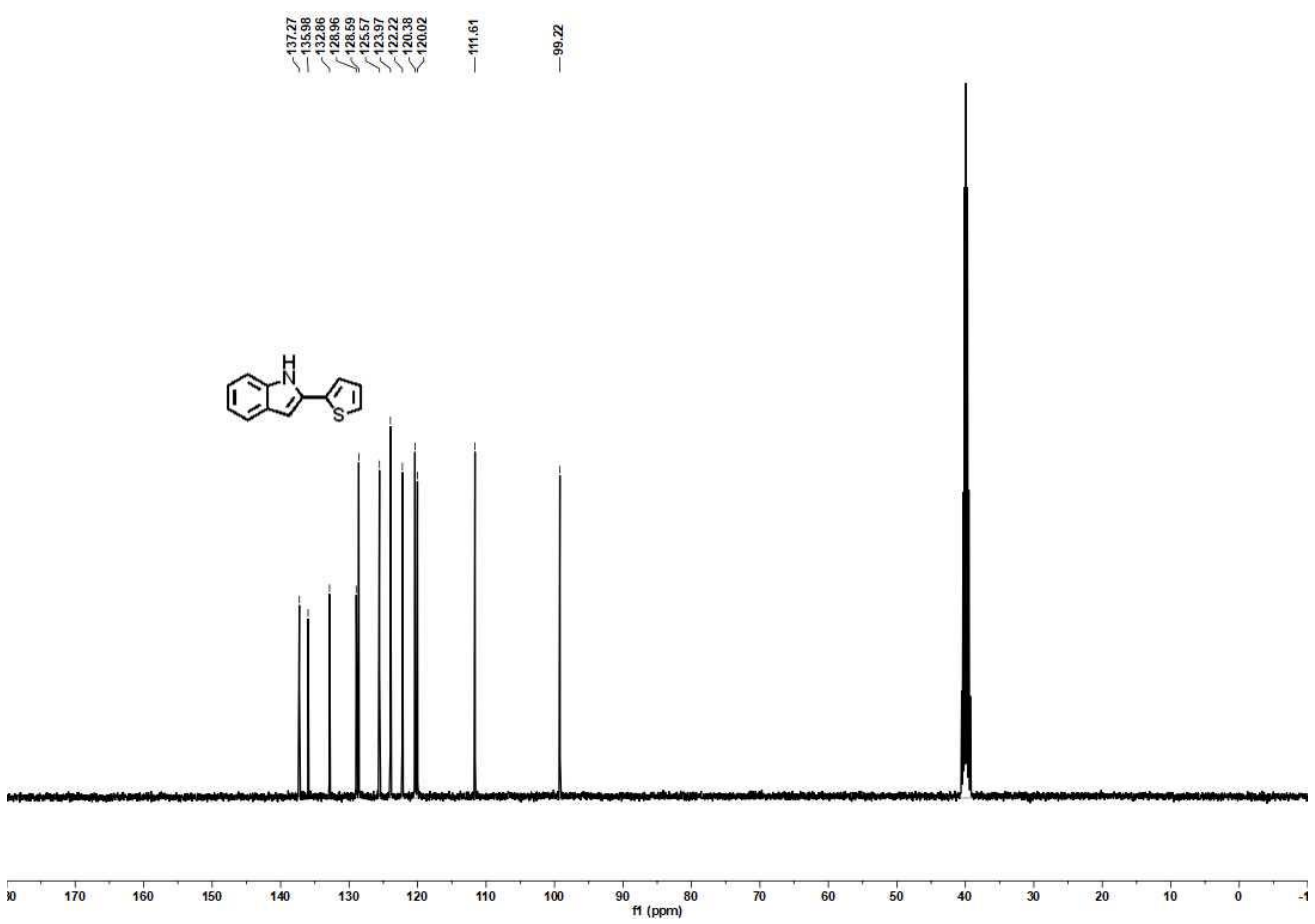

3j

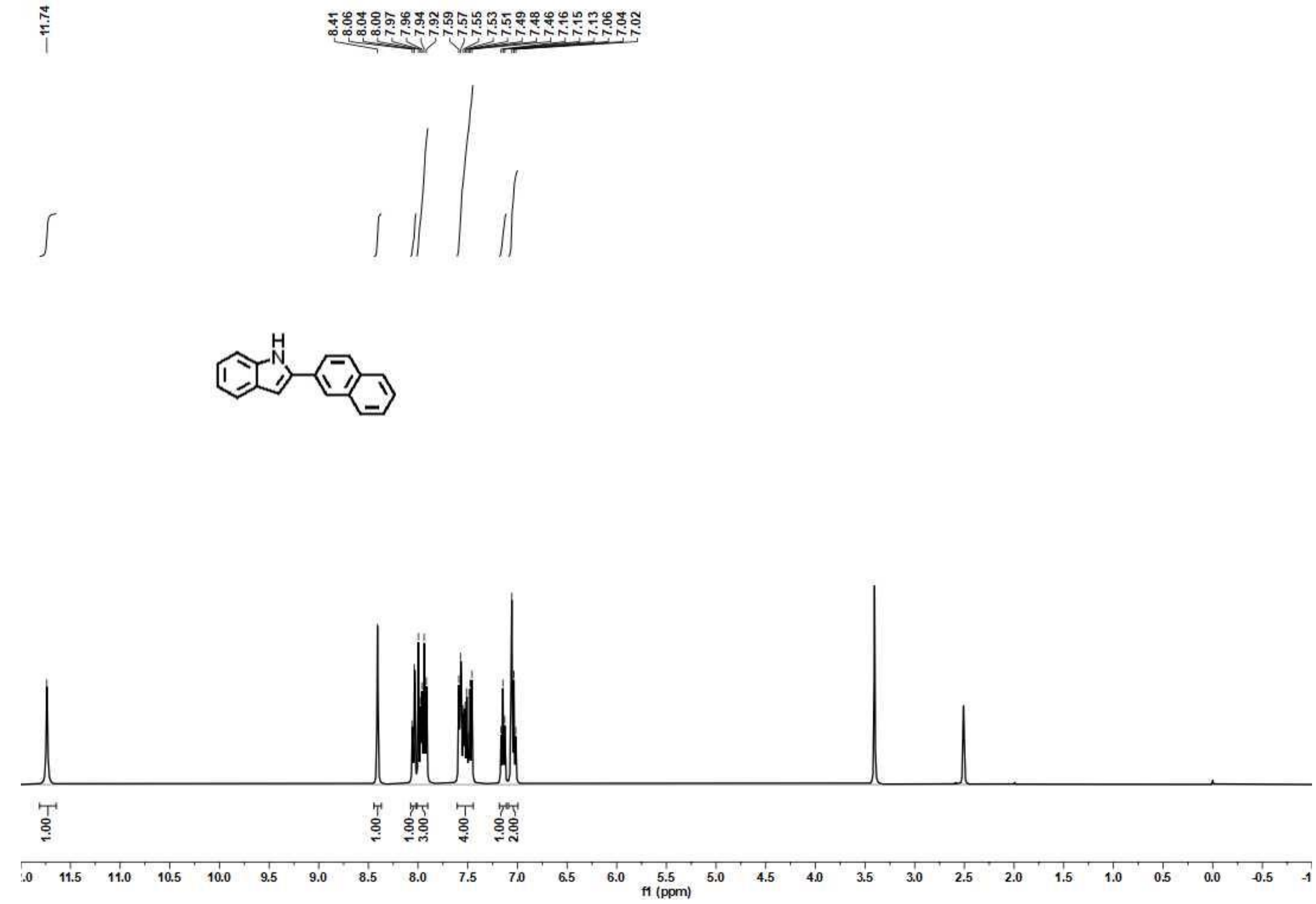
11.74
8.41
8.06
8.04
8.00
7.97
7.96
7.94
7.92
7.59
7.57
7.55
7.53
7.51
7.49
7.48
7.46
7.16
7.15
7.13
7.06
7.04
7.02
H
N
1.00
1.00
1.00
3.00
4.00
1.00
2.00
f1 (ppm)

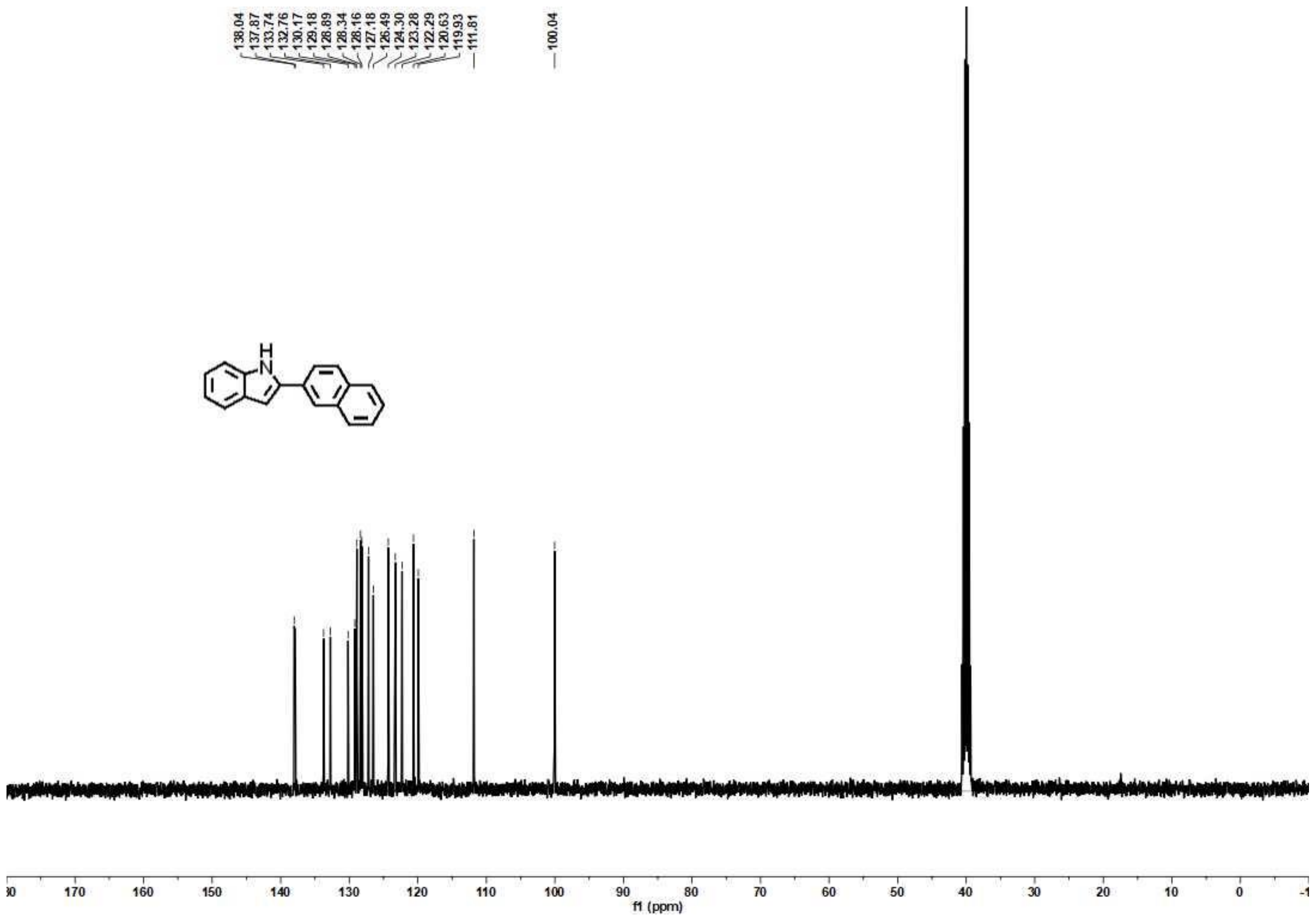
138.04
137.87
133.74
132.76
130.17
129.18
128.89
128.34
128.16
127.18
126.49
124.30
123.28
122.29
120.63
119.93
111.81
100.04
H
N
f1 (ppm)

3k

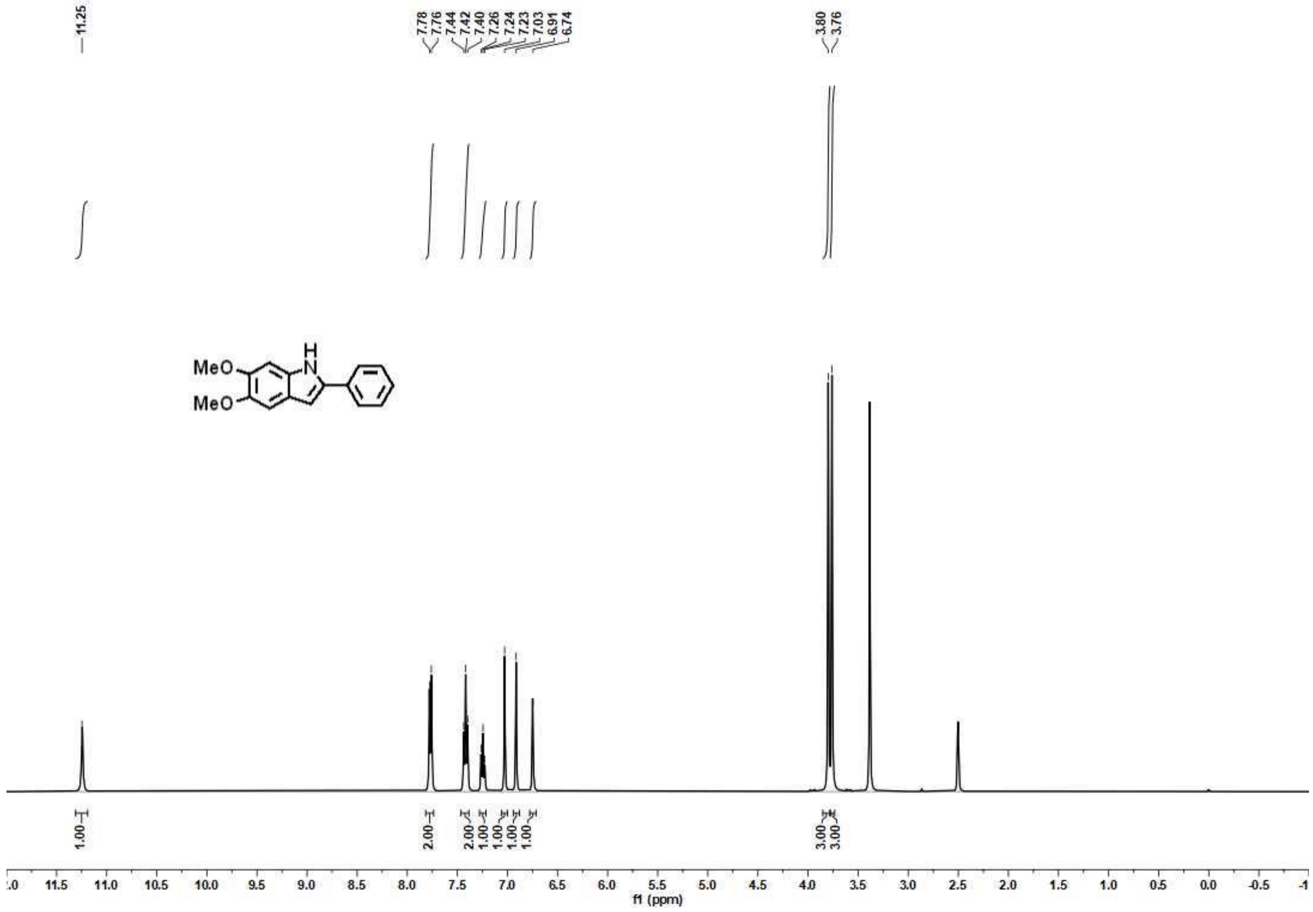
11.25
7.78 7.76 7.44 7.42 7.40 7.26 7.24 7.23 7.03 6.91 6.74
3.80 3.76
MeO
MeO
H
N
1.00
2.00 2.00 1.00 1.00 1.00 1.00
3.00 3.00
f1 (ppm)

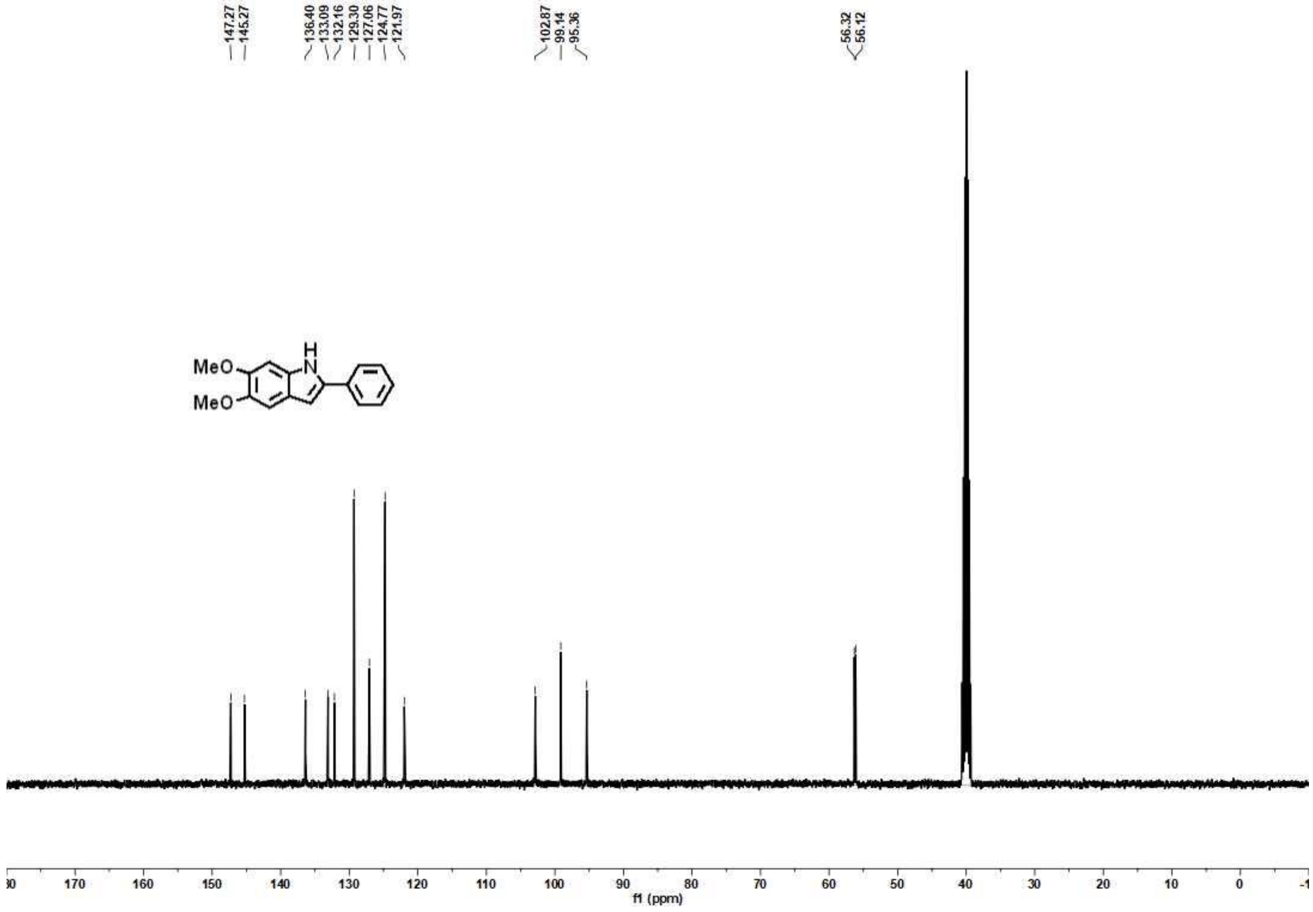
147.27 145.27
136.40 133.09 132.16 129.30 127.06 124.77 121.97
102.87 99.14 95.36
56.32 56.12
MeO
MeO
H
N
f1 (ppm)

3l

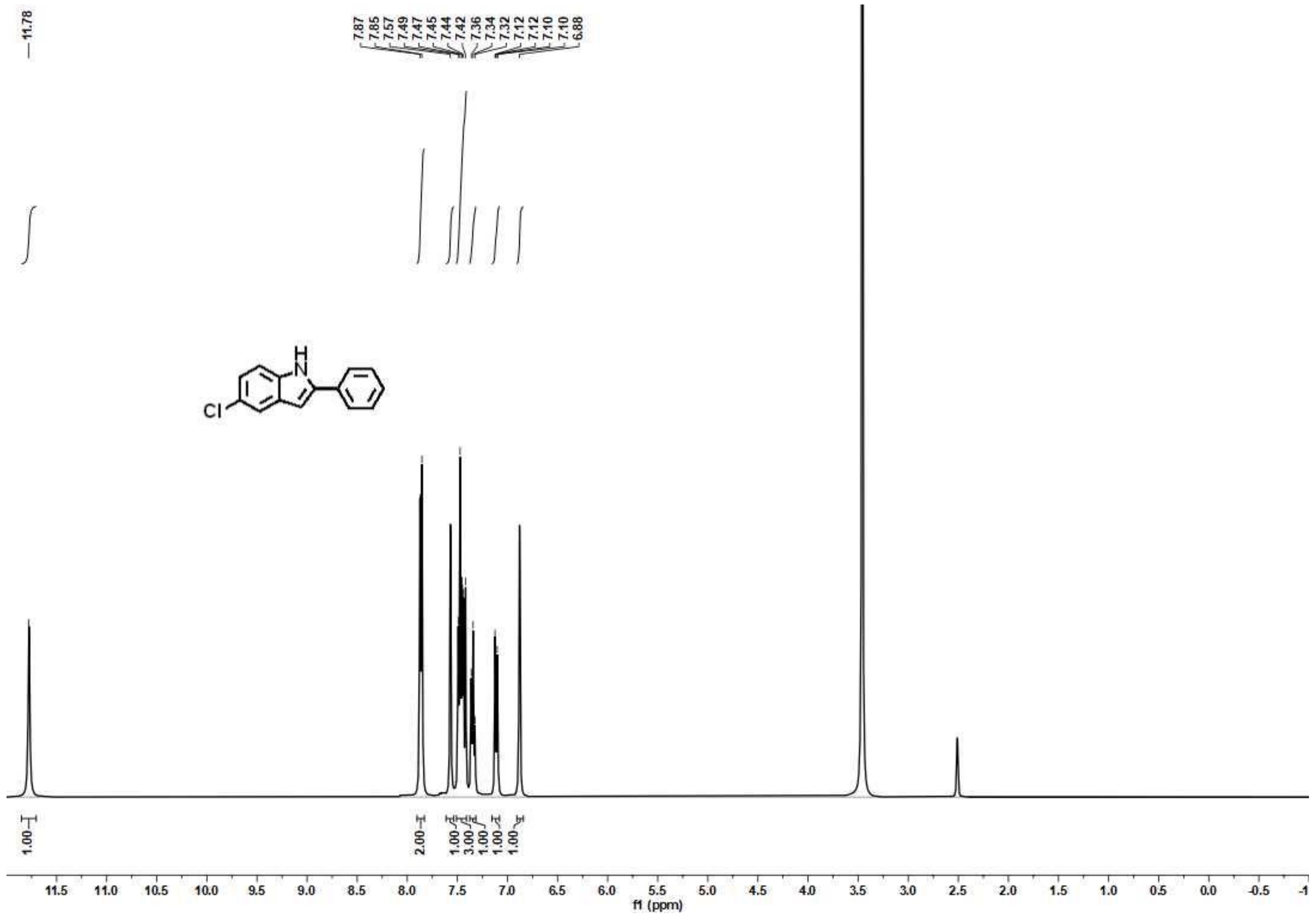
11.78
7.87
7.85
7.57
7.49
7.47
7.45
7.44
7.42
7.36
7.34
7.32
7.12
7.12
7.10
6.88
Cl
H
N
1.00
2.00
1.00
1.00
3.00
1.00
1.00
1.00
f1 (ppm)

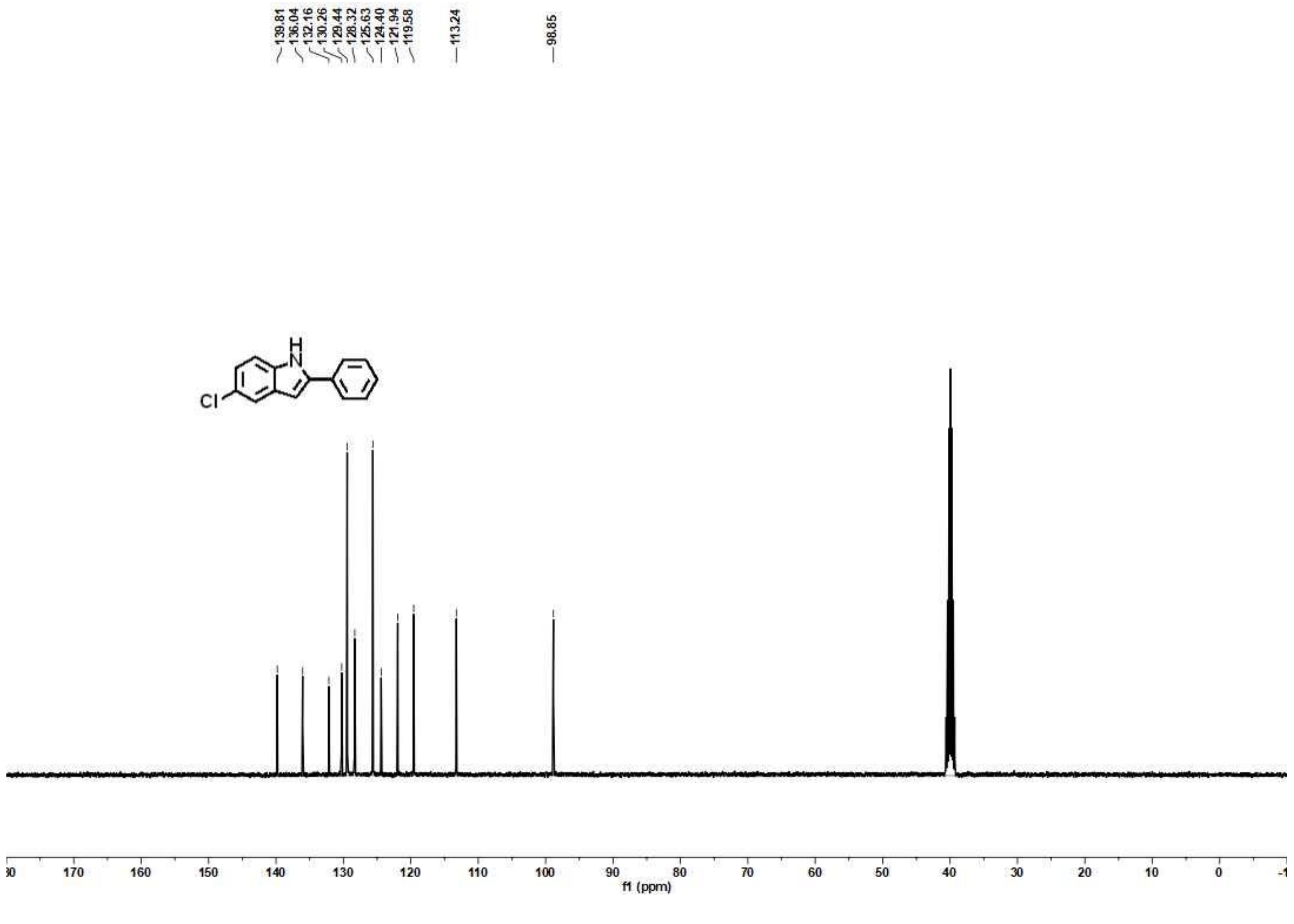
139.81
136.04
132.16
130.26
129.44
128.32
125.63
124.40
121.94
119.58
113.24
98.85
Cl
H
N
f1 (ppm)

3m

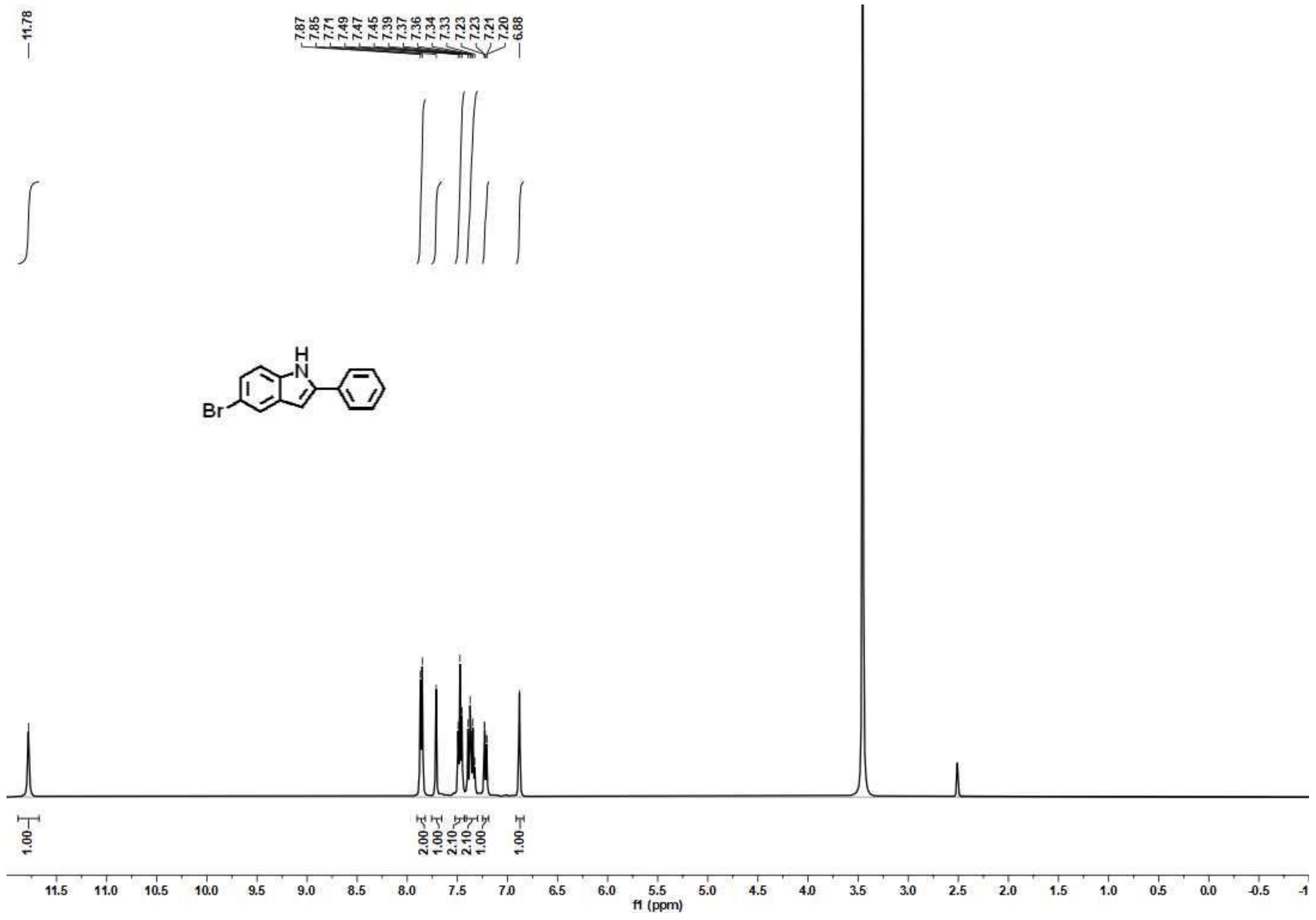

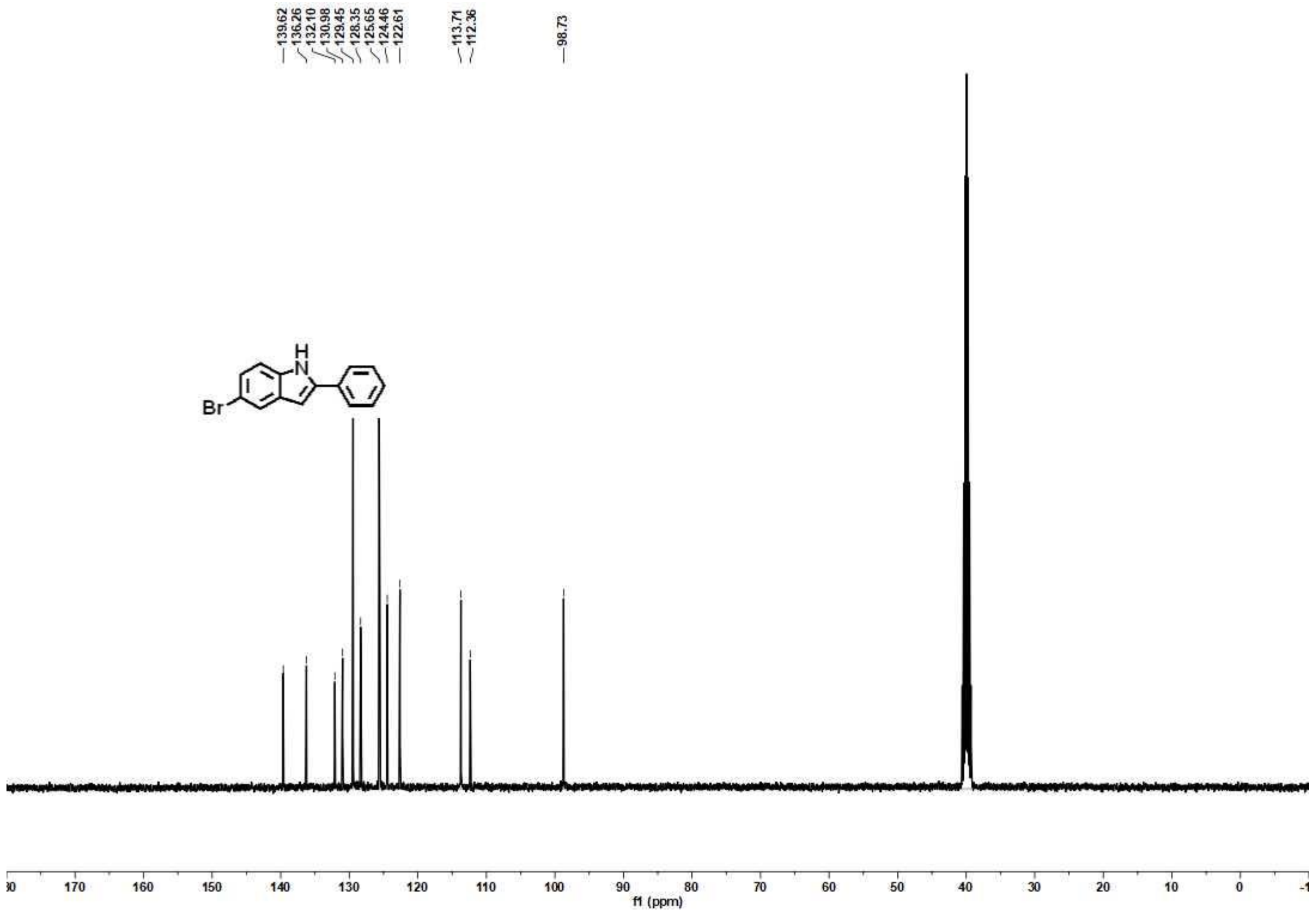

3n

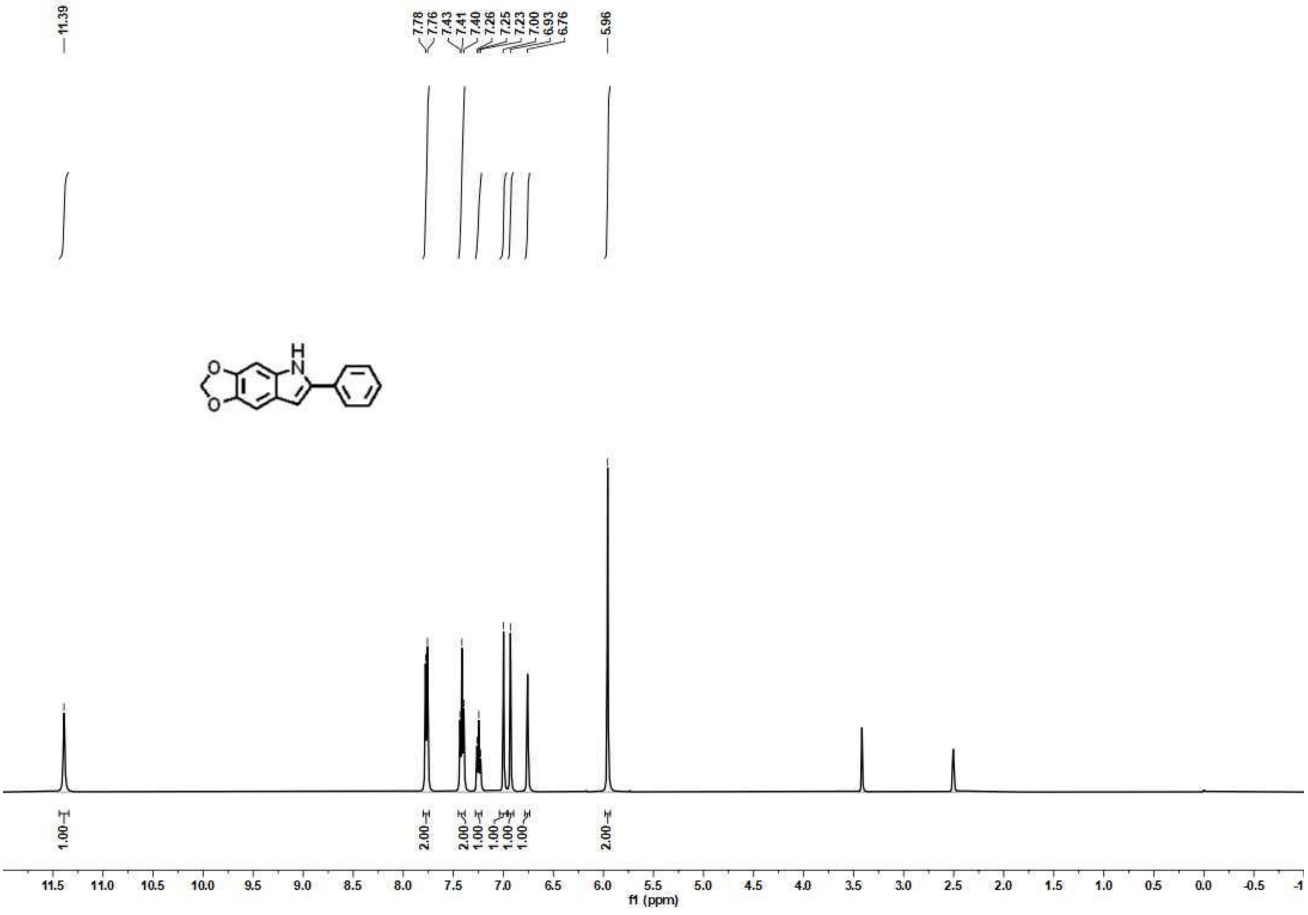
11.39
7.78
7.76
7.43
7.41
7.40
7.26
7.25
7.23
7.00
6.93
6.76
5.96
1.00
2.00
2.00
1.00
1.00
1.00
1.00
2.00
f1 (ppm)

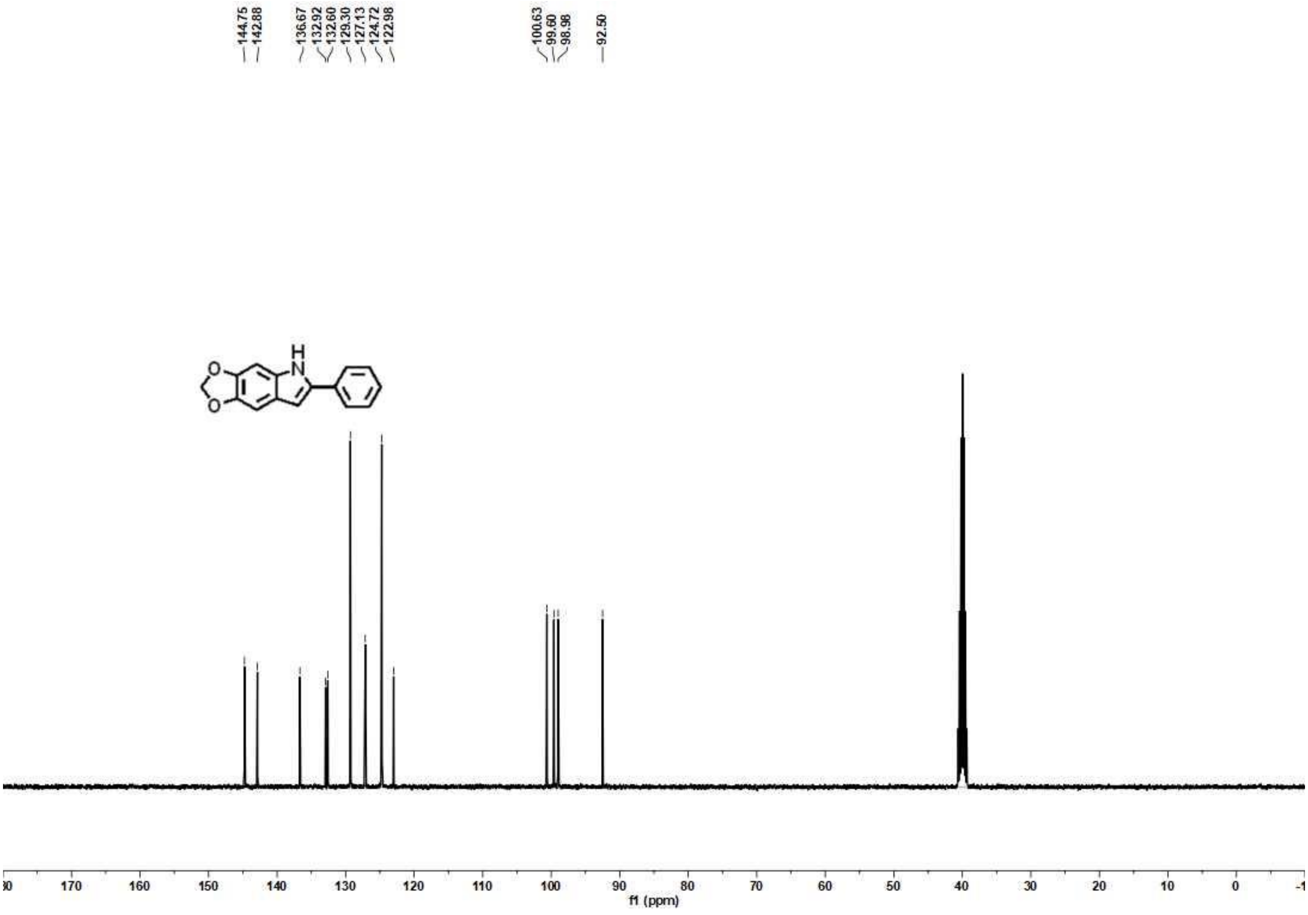
144.75
142.88
136.67
132.92
132.60
129.30
127.13
124.72
122.98
100.63
99.60
98.98
92.50
f1 (ppm)

5a

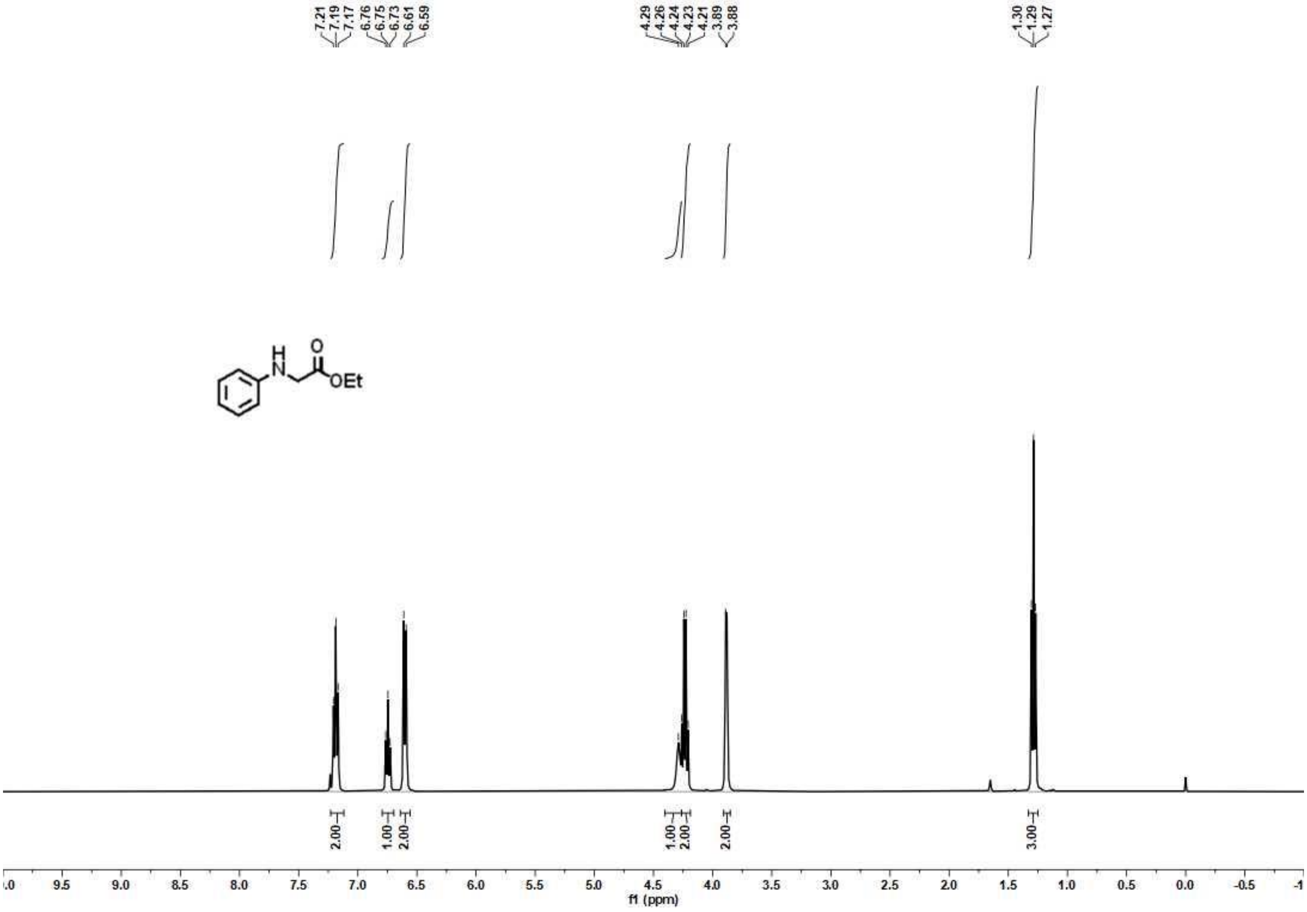

7.21
7.19
7.17
6.76
6.75
6.73
6.61
6.59
4.29
4.26
4.24
4.23
4.21
3.89
3.88
1.30
1.29
1.27
OEt
2.00
1.00
2.00
1.00
2.00
2.00
3.00
f1 (ppm)

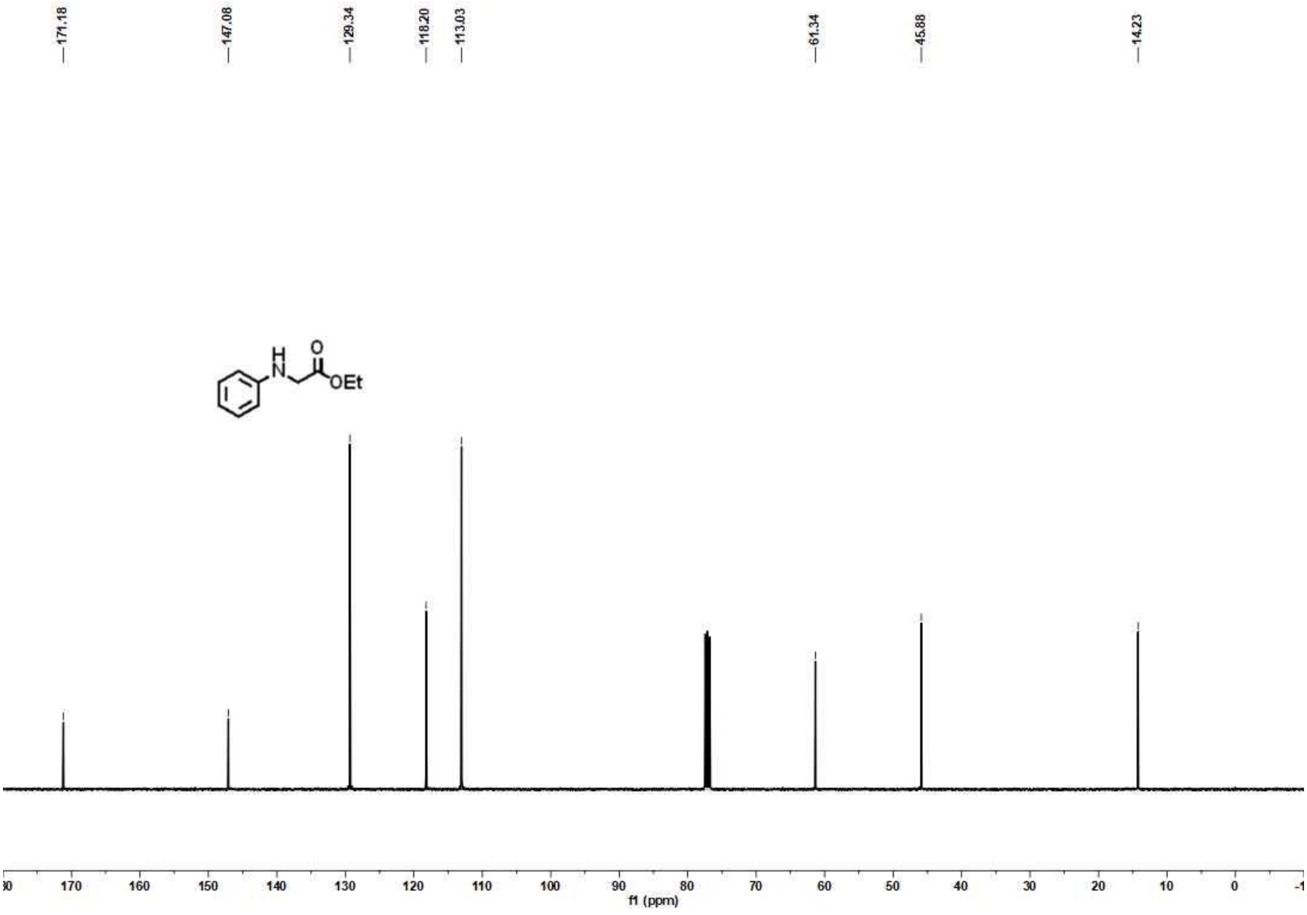

171.18
147.08
129.34
118.20
113.03
61.34
45.88
14.23
OEt
f1 (ppm)

5b

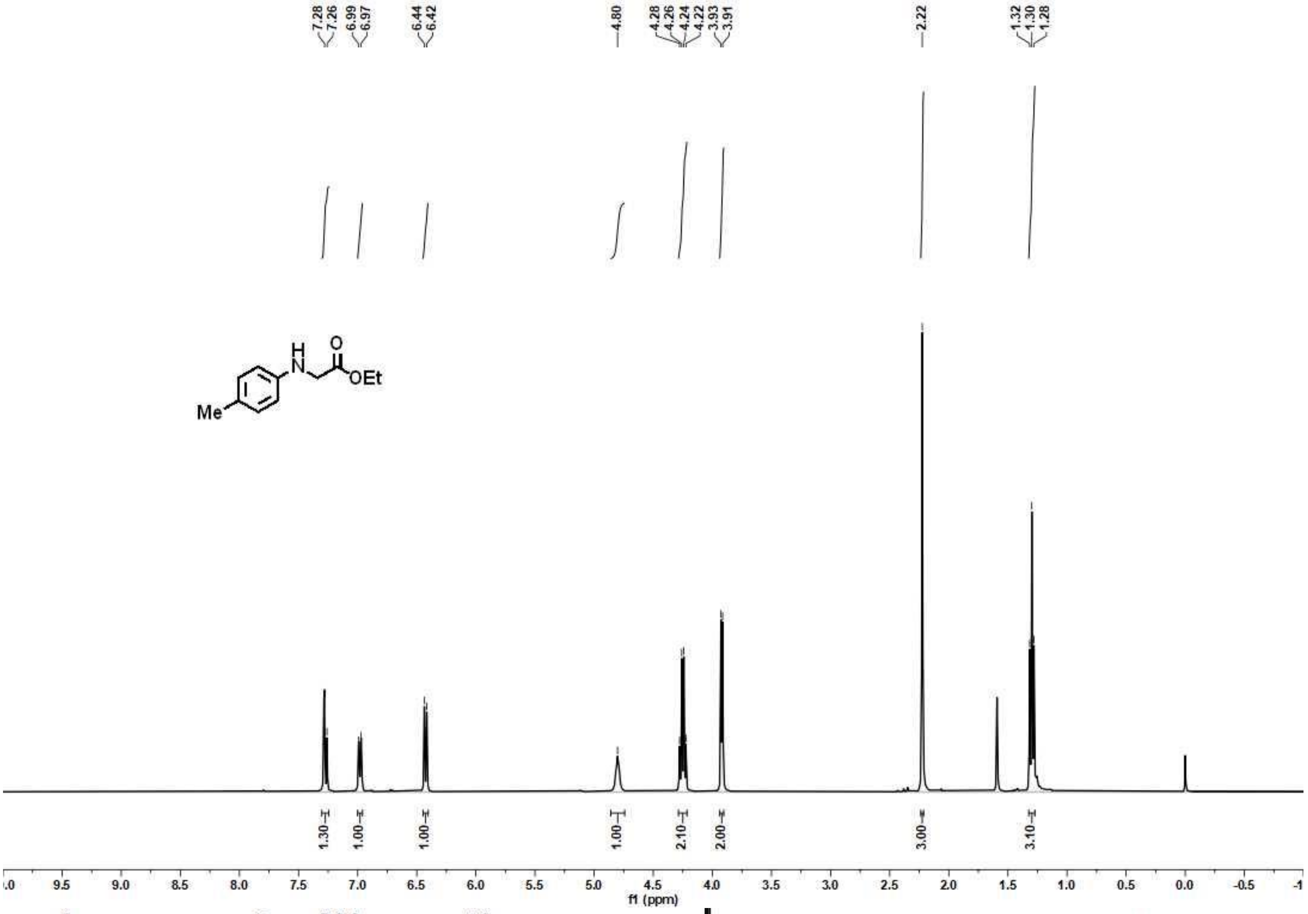

7.28
7.26
6.99
6.97
6.44
6.42
4.80
4.28
4.26
4.24
4.22
3.93
3.91
2.22
1.32
1.30
1.28
Me
N
H
O
OEt
1.30
1.00
1.00
1.00
2.10
2.00
3.00
3.10
f1 (ppm)

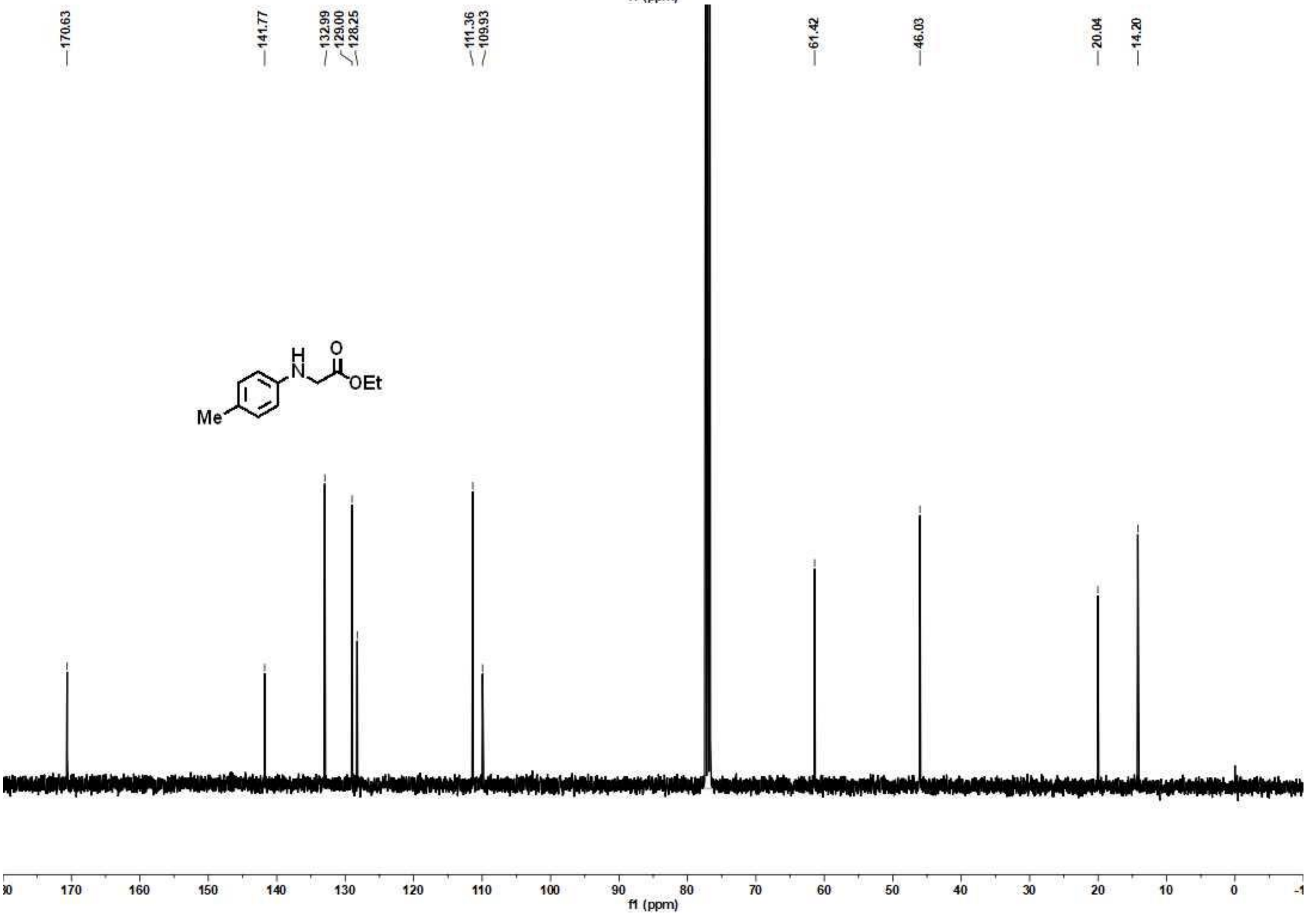

170.63
141.77
132.99
129.00
128.25
111.36
109.93
61.42
46.03
20.04
14.20
Me
N
H
O
OEt
f1 (ppm)

5c

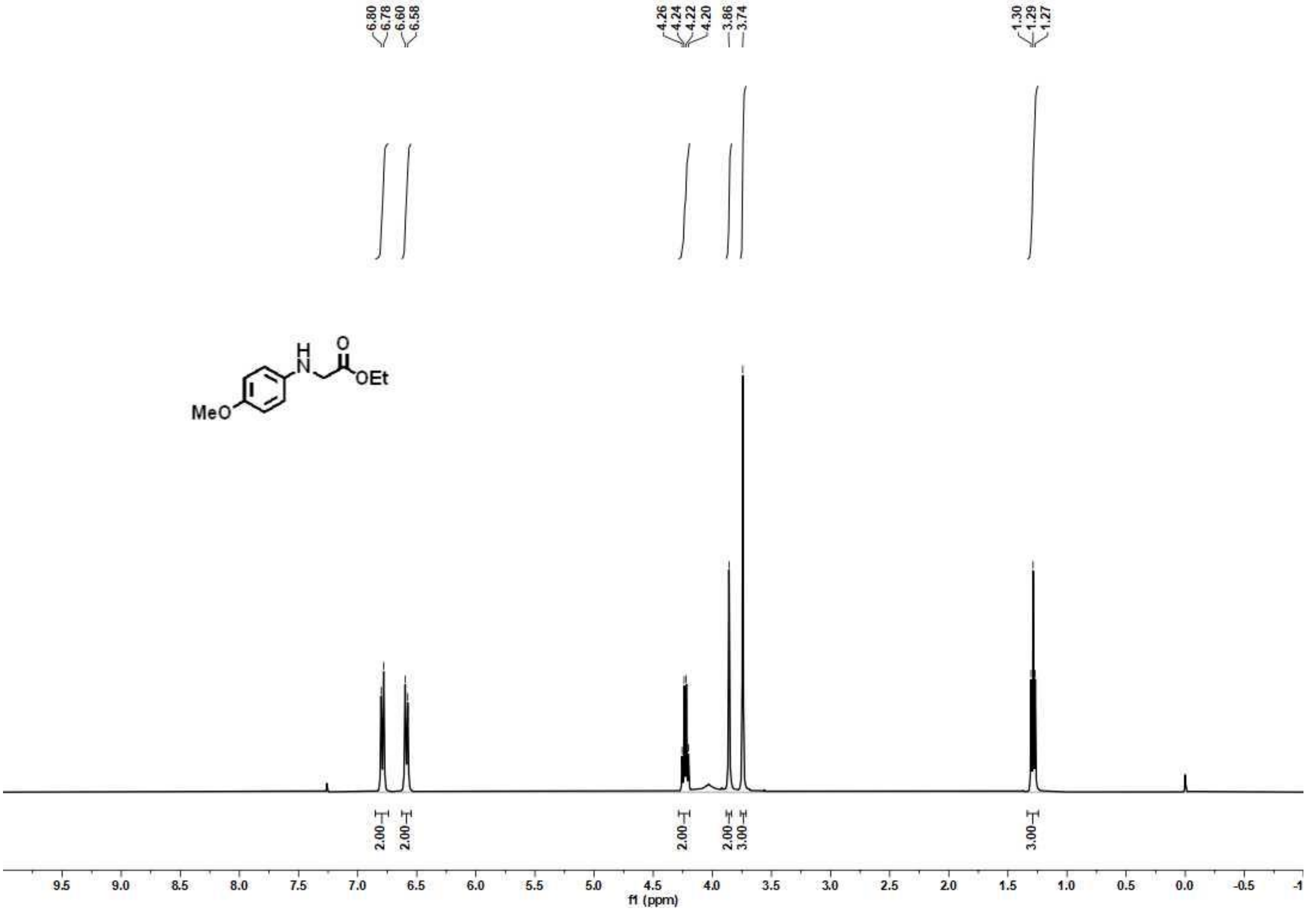
6.80
6.78
6.60
6.58
4.26
4.24
4.22
4.20
3.86
3.74
1.30
1.29
1.27
MeO
H
N
O
OEt
2.00
2.00
2.00
2.00
3.00
3.00
f1 (ppm)

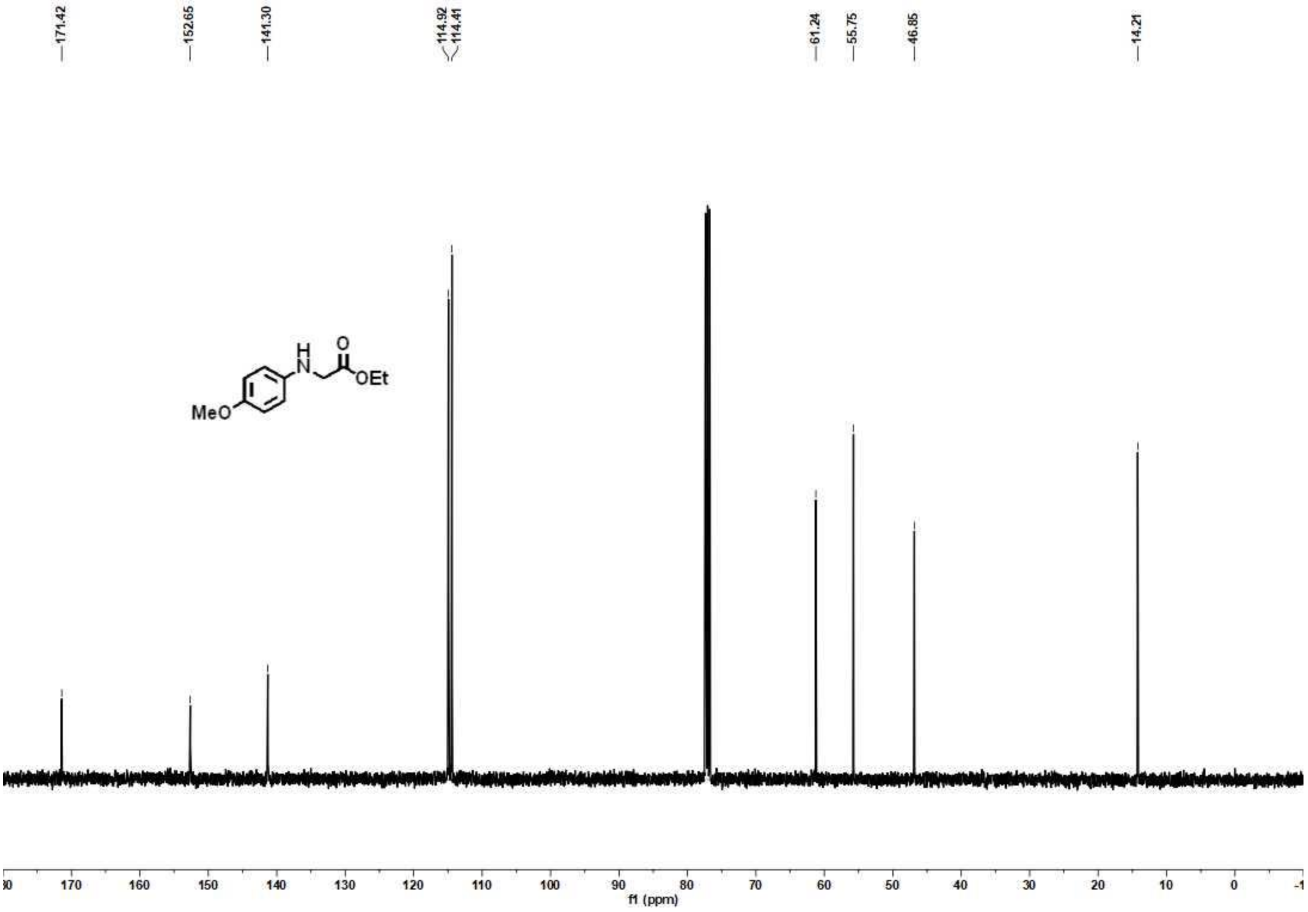
171.42
152.65
141.30
114.92
114.41
61.24
55.75
46.85
14.21
MeO
H
N
O
OEt
f1 (ppm)

5d

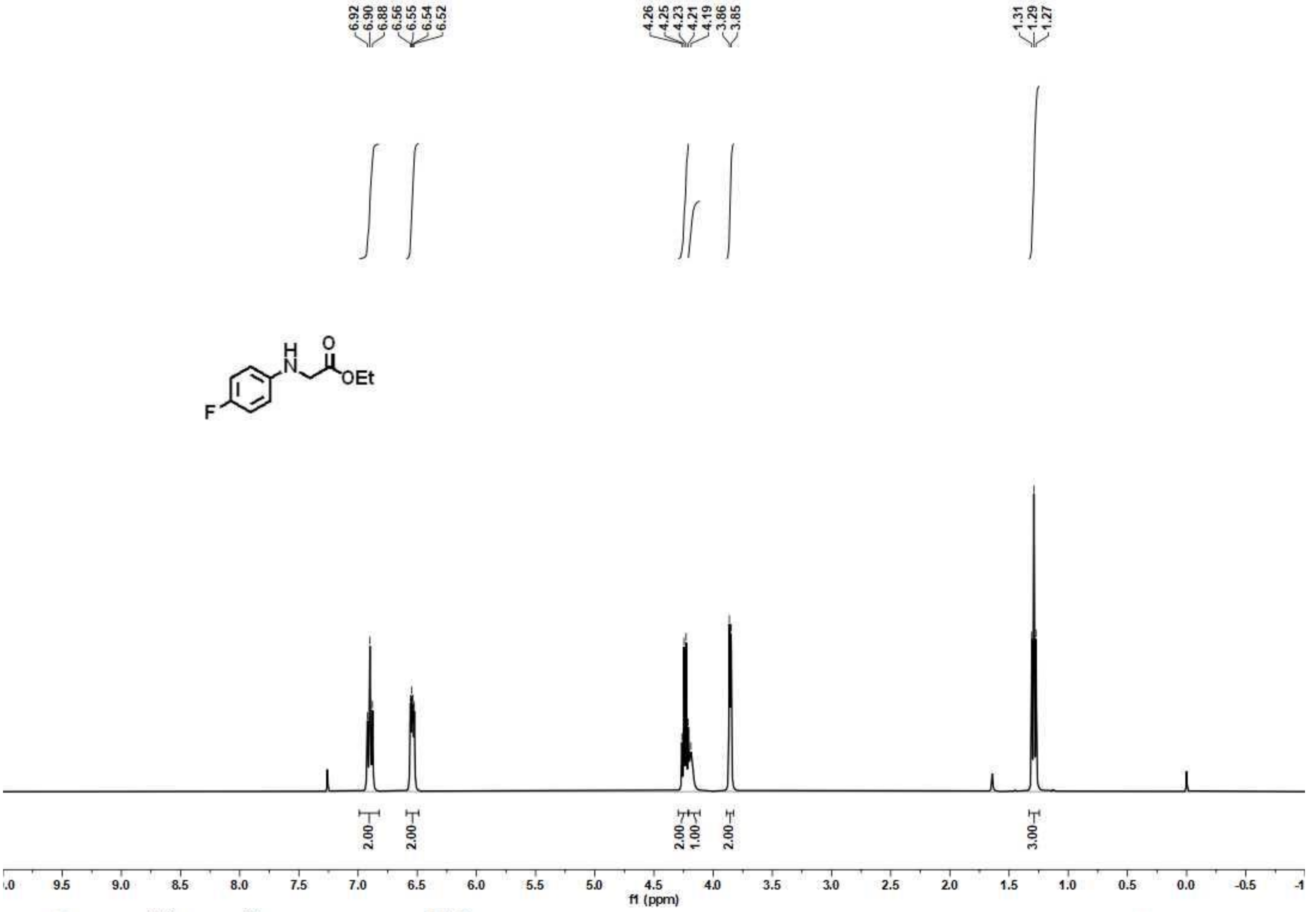
6.92
6.90
6.88
6.56
6.55
6.54
6.52
4.26
4.25
4.23
4.21
4.19
3.86
3.85
1.31
1.29
1.27
OEt
F
2.00
2.00
2.00
1.00
2.00
3.00
f1 (ppm)

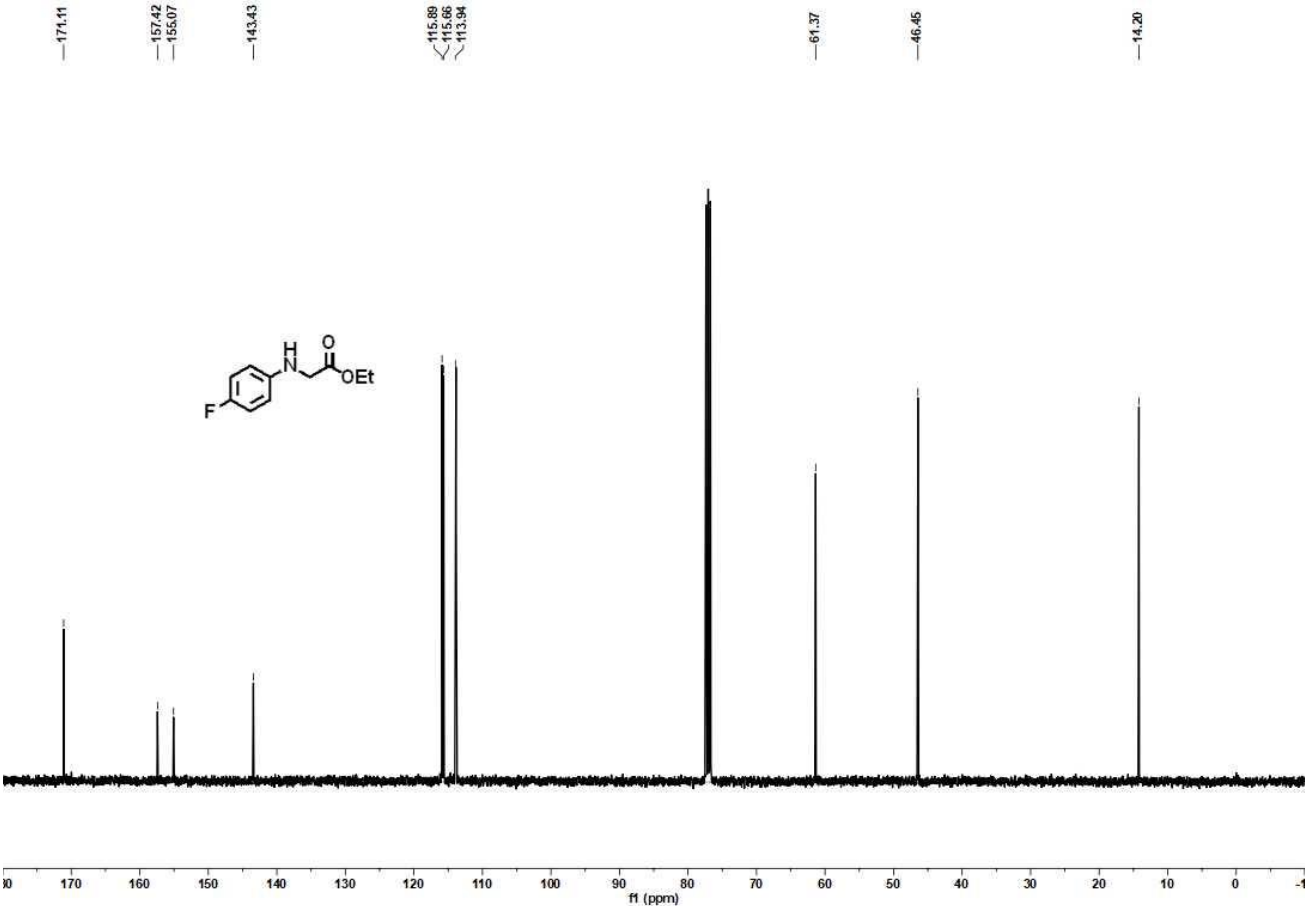
171.11
157.42
155.07
143.43
115.89
115.66
113.94
61.37
46.45
14.20
OEt
F
f1 (ppm)

5e

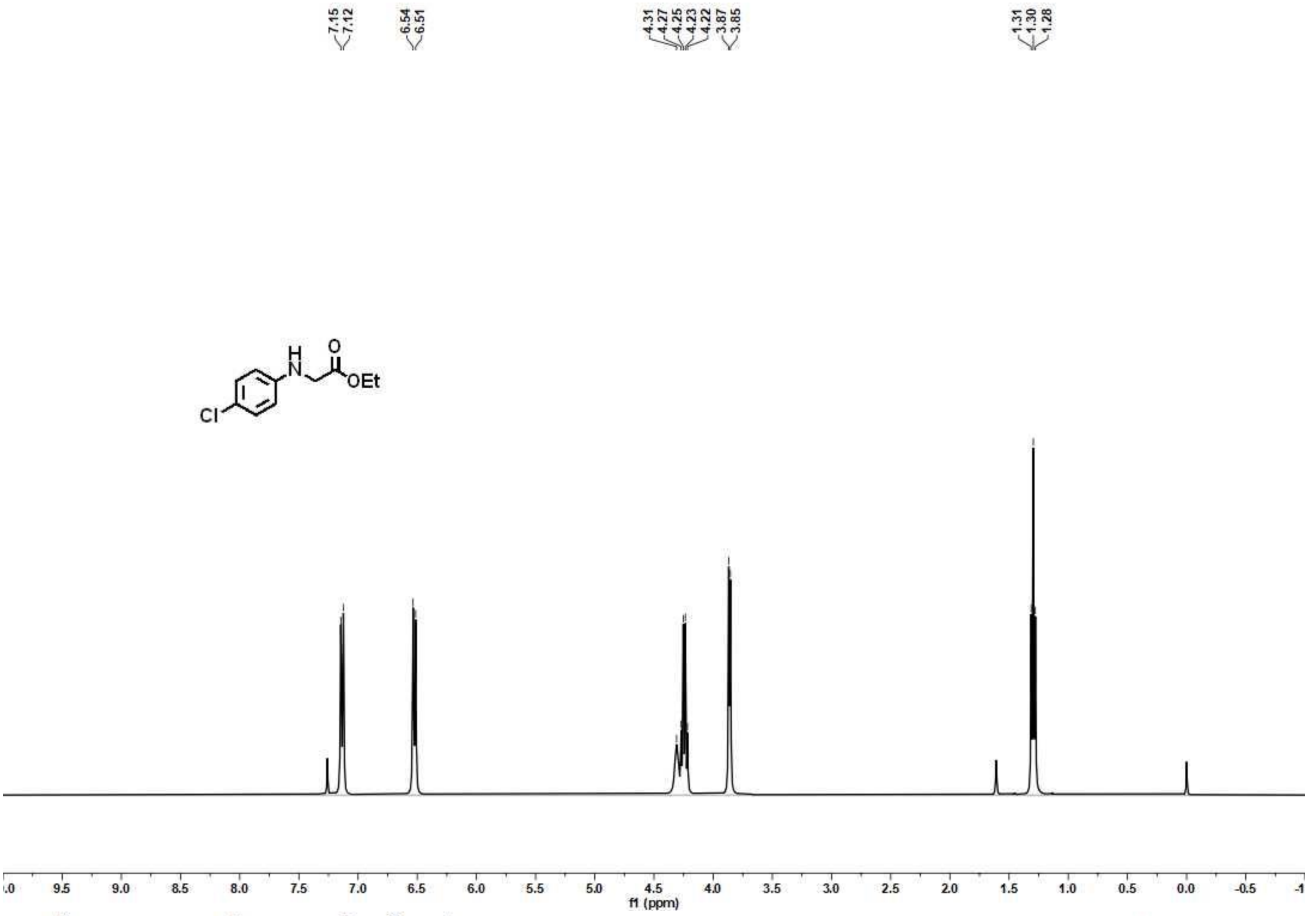
7.15
7.12
6.54
6.51
4.31
4.27
4.25
4.23
4.22
3.87
3.85
1.31
1.30
1.28
H
N
O
OEt
Cl
f1 (ppm)

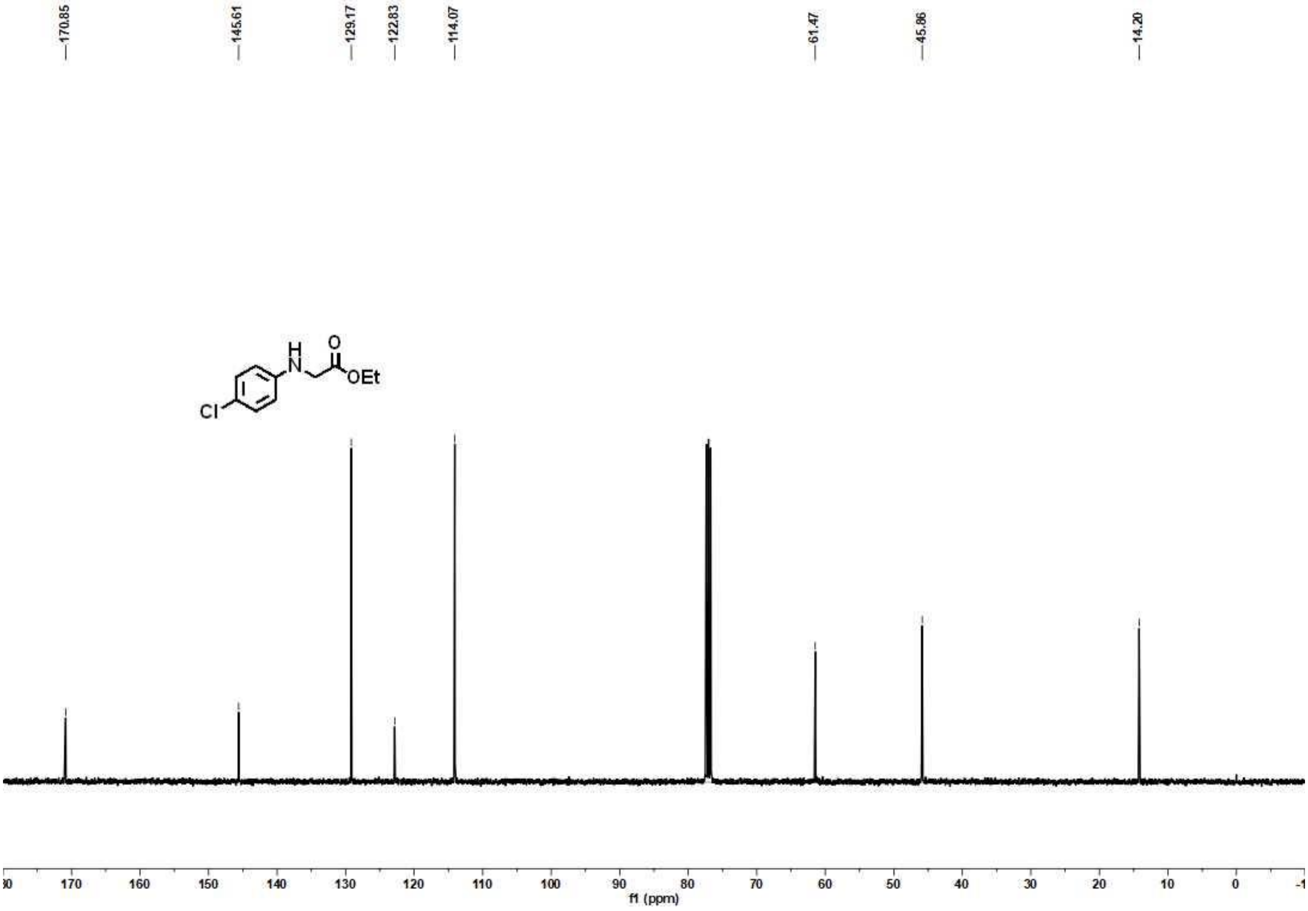
170.85
145.61
129.17
122.83
114.07
61.47
45.86
14.20
H
N
O
OEt
Cl
f1 (ppm)

5f

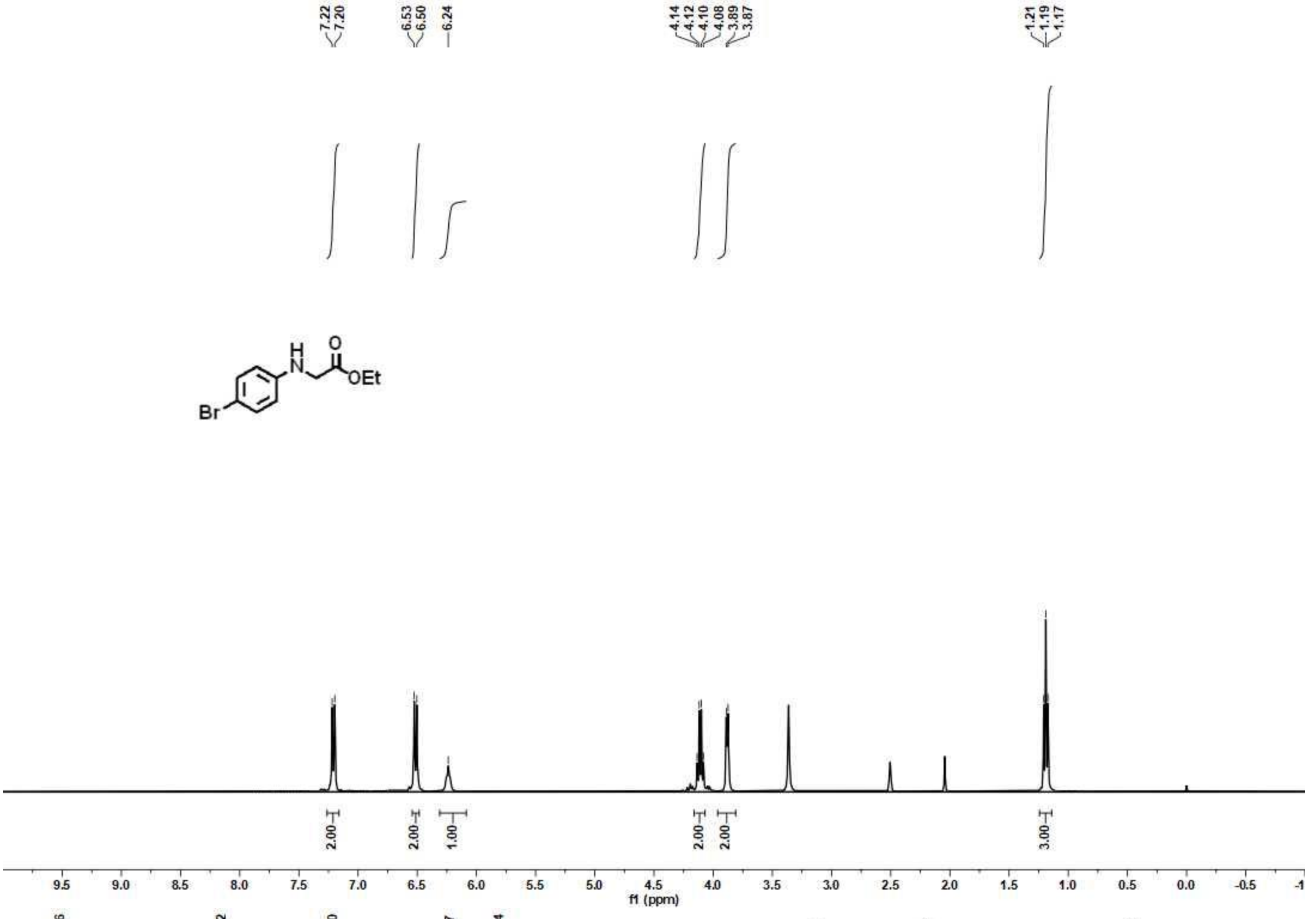

7.22
7.20
6.53
6.50
6.24
4.14
4.12
4.10
4.08
3.89
3.87
1.21
1.19
1.17
2.00
2.00
1.00
2.00
2.00
3.00
f1 (ppm)
OEt
Br

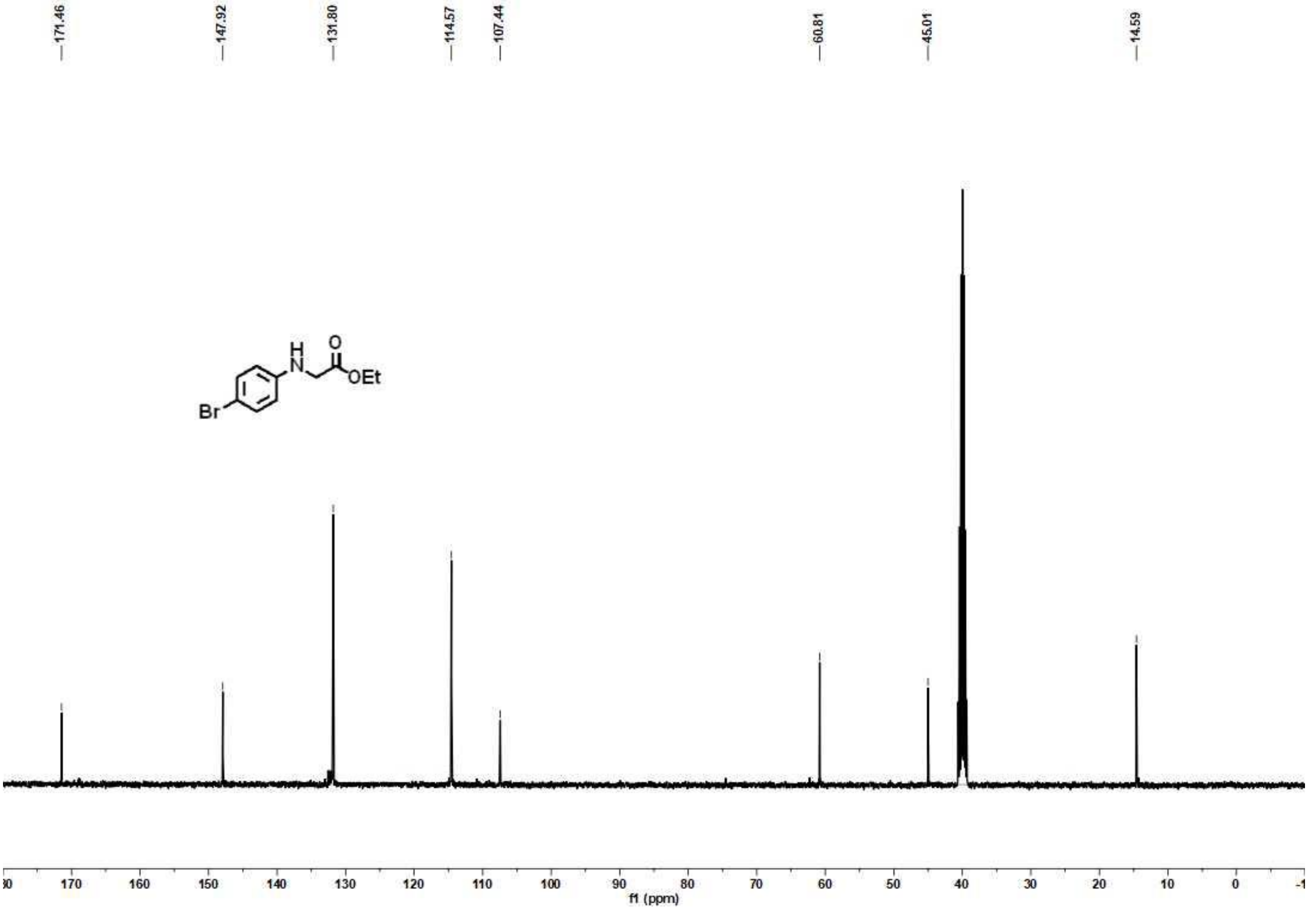

171.46
147.92
131.80
114.57
107.44
60.81
45.01
14.59
OEt
Br
f1 (ppm)

5g

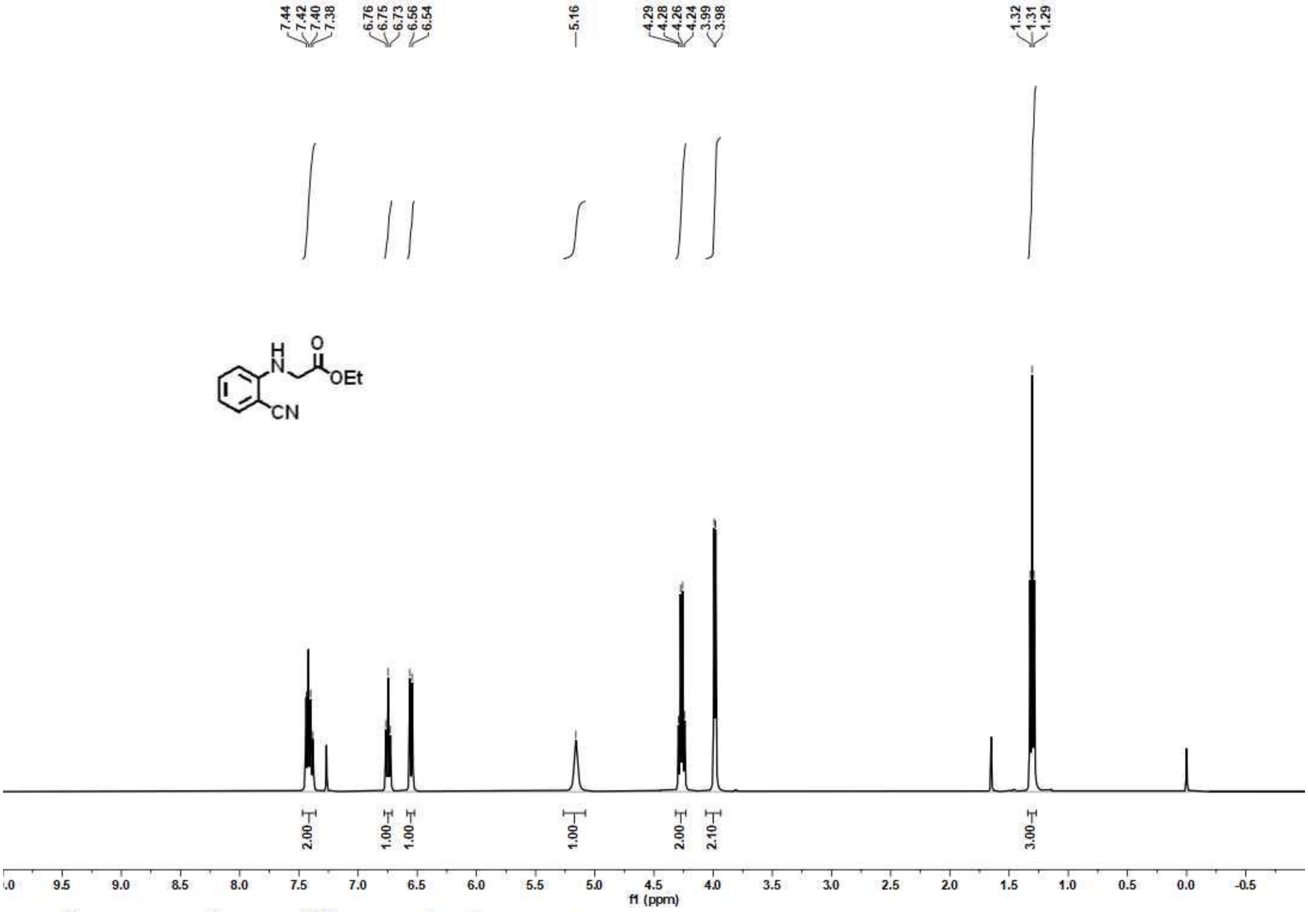
7.44
7.42
7.40
7.38
6.76
6.75
6.73
6.56
6.54
5.16
4.29
4.28
4.26
4.24
3.99
3.98
1.32
1.31
1.29
OEt
CN
2.00
1.00
1.00
1.00
2.00
2.10
3.00
f1 (ppm)

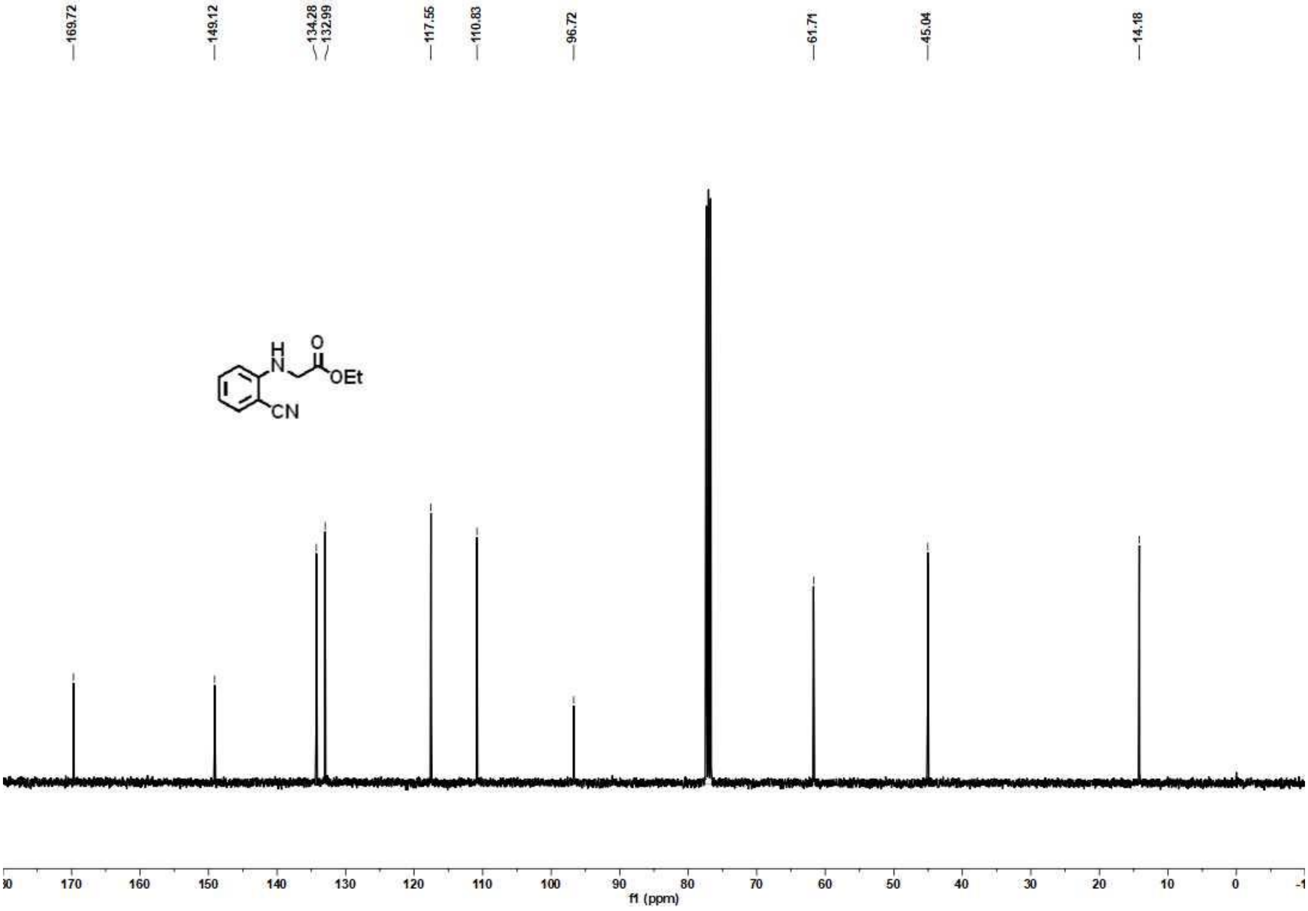
169.72
149.12
134.28
132.99
117.55
110.83
96.72
61.71
45.04
14.18
OEt
CN
f1 (ppm)

5h

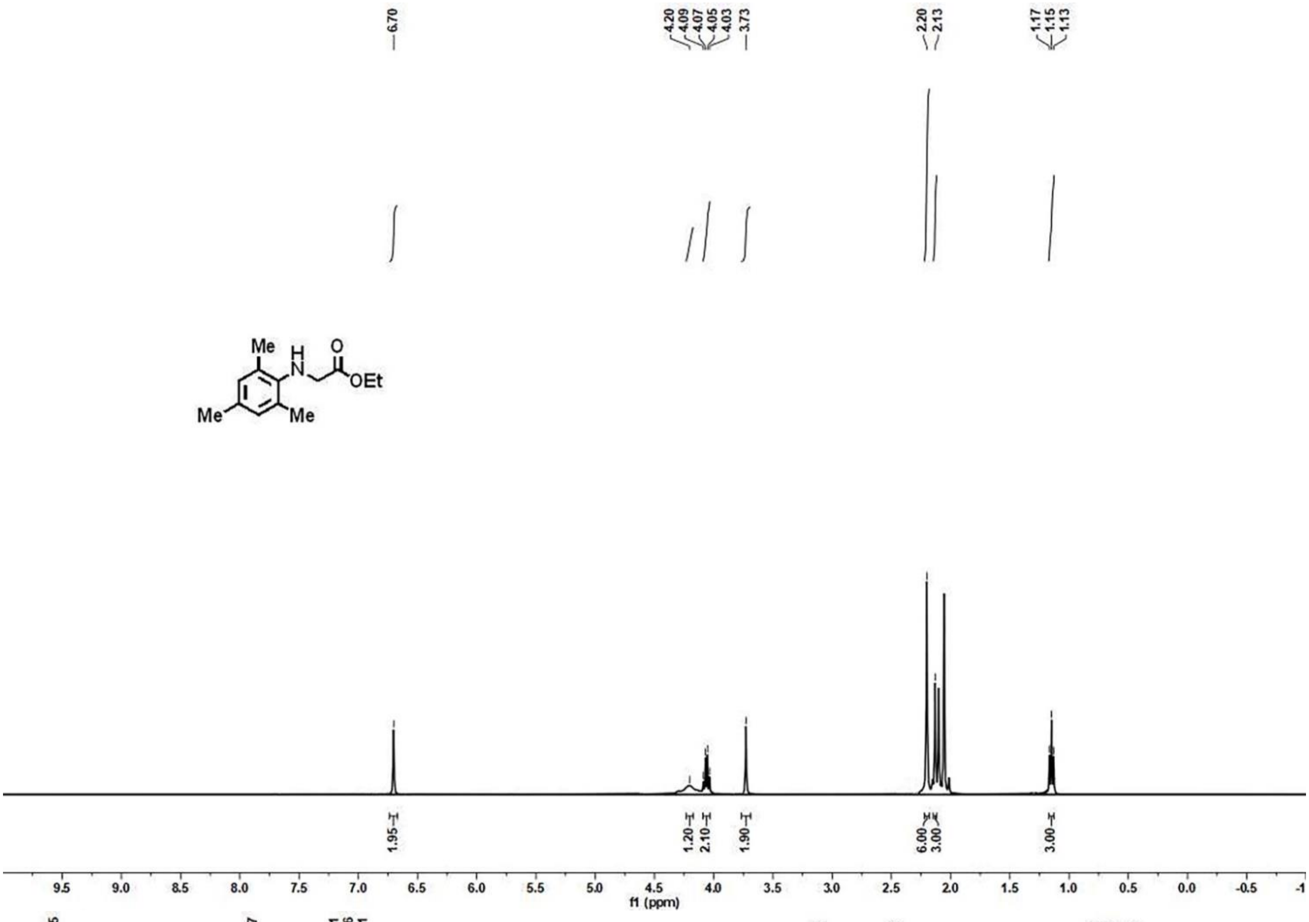

6.70
4.20
4.09
4.07
4.05
4.03
3.73
2.20
2.13
1.17
1.15
1.13
Me
H
N
O
OEt
Me
Me
1.95
1.20
2.10
1.90
6.00
3.00
3.00
9.5
9.0
8.5
8.0
7.5
7.0
6.5
6.0
5.5
5.0
4.5
4.0
3.5
3.0
2.5
2.0
1.5
1.0
0.5
0.0
-0.5
f1 (ppm)

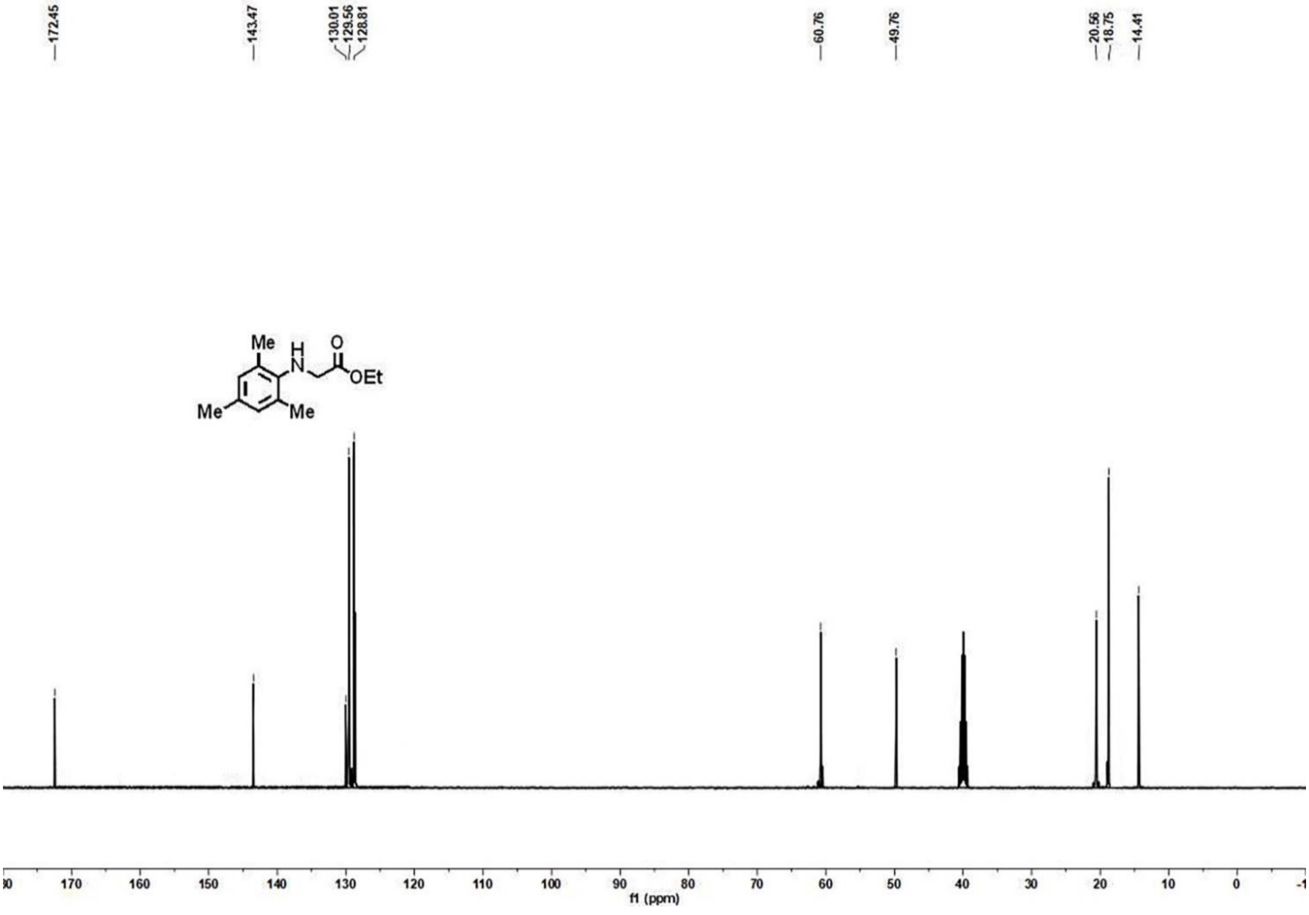

172.45
143.47
130.01
129.56
128.81
60.76
49.76
20.56
18.75
14.41
Me
H
N
O
OEt
Me
Me
170
160
150
140
130
120
110
100
90
80
70
60
50
40
30
20
10
0
f1 (ppm)

5i

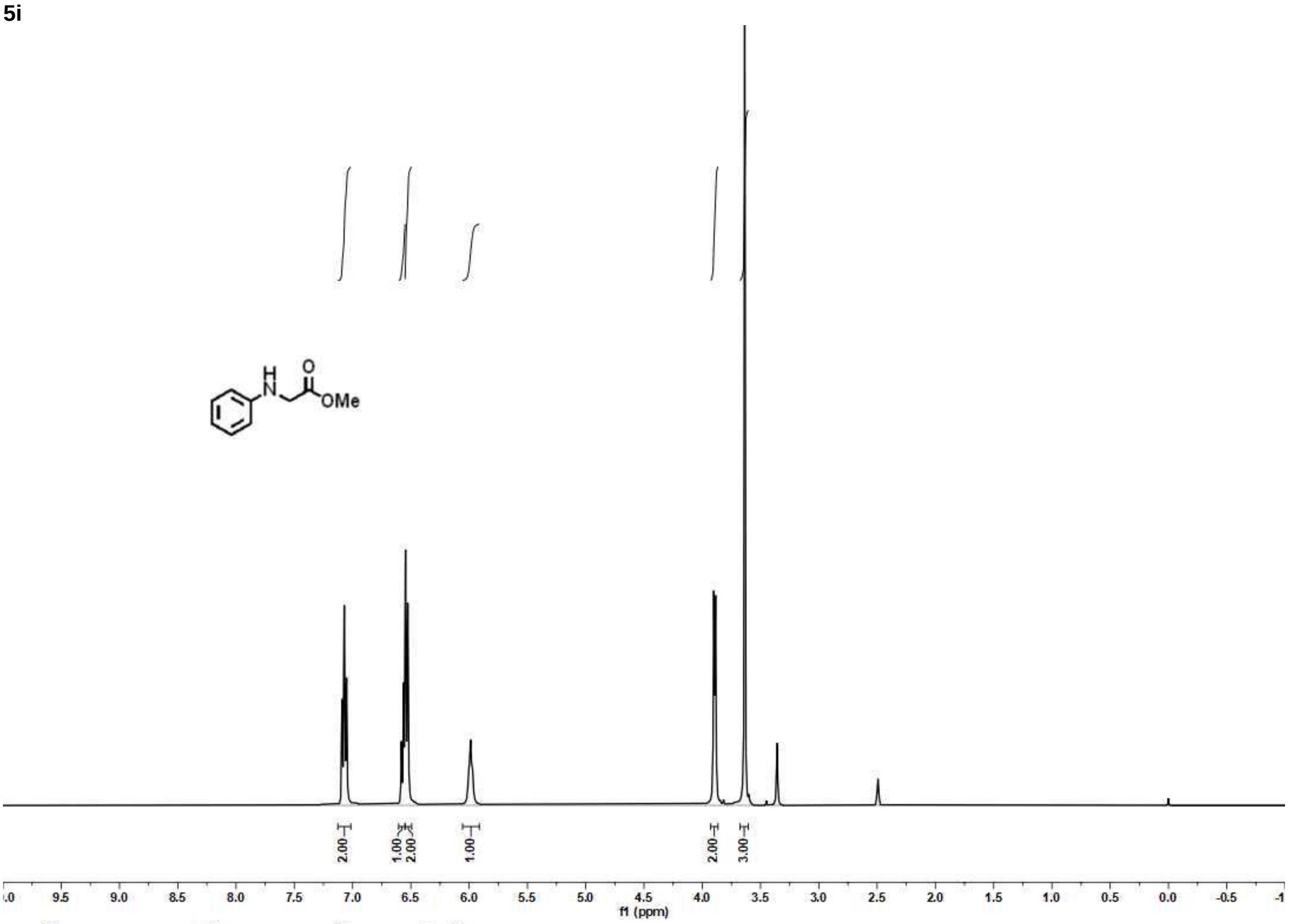

OMe
2.00
1.00
2.00
1.00
2.00
3.00
f1 (ppm)

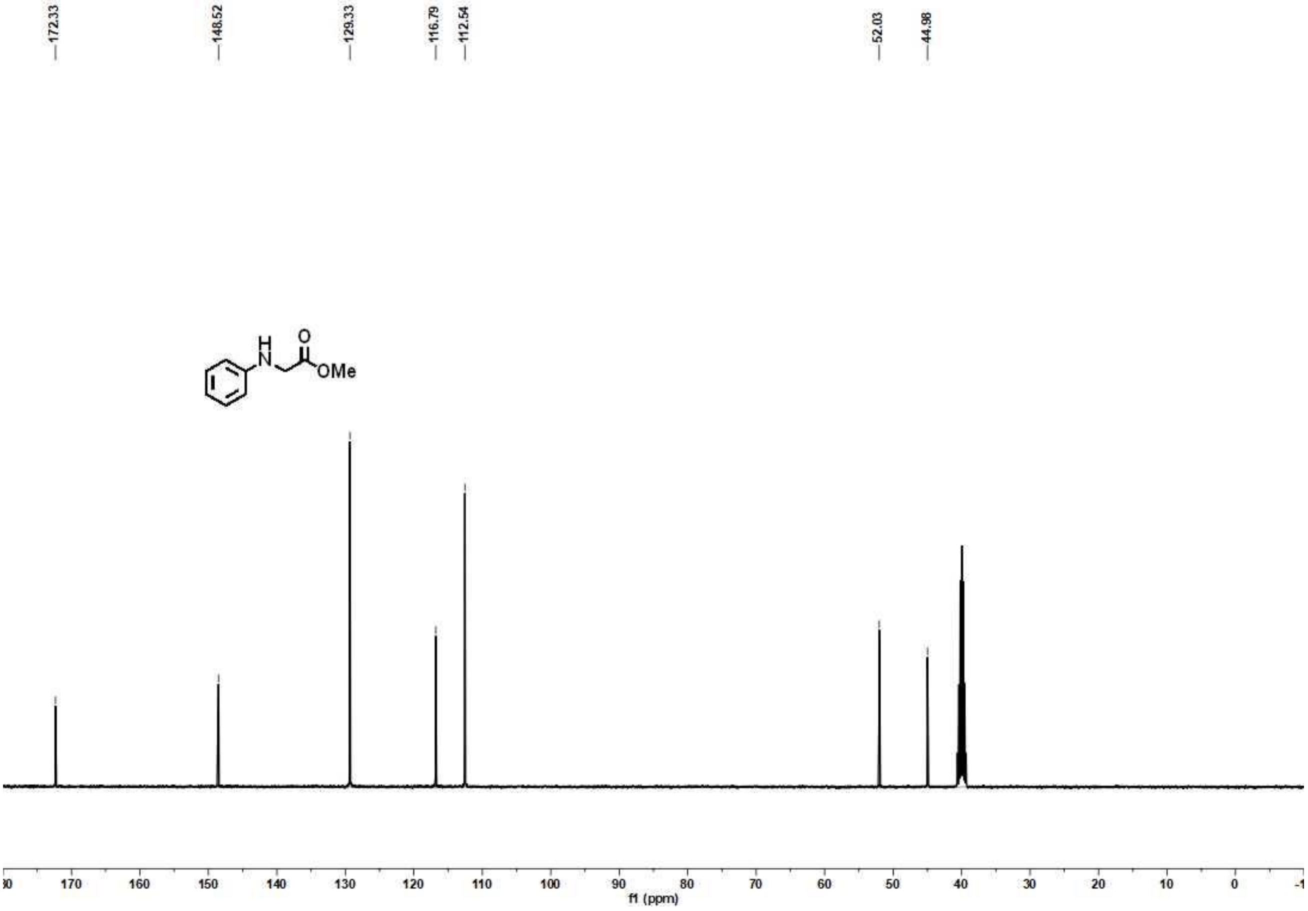

172.33
148.52
129.33
116.79
112.54
52.03
44.98
OMe
f1 (ppm)

3 References

[1] B. Li, R. Gong, W. Wang, X. Huang, W. Zhang, H. Li, C. Hu, B. Tan, *Macromolecules* **2011**, *44*, 2410-2414.

[2] a) V. V. Angeles-Dunham, D. M. Nickerson, D. M. Ray, A. E. Mattson, *Angew. Chem. Int. Ed.* **2014**, *53*, 14538-14541; b) M. Wang, B. Li, B. Gong, H. Yao, A. Lin, *Chem. Commun.* **2022**, *58*, 2850-2853.

[3] H. Moon, S. Han, S. L. Scott, *Chem. Sci.* **2020**, *11*, 3702-3712.

[4] a) C. Reichardt, *Chem. Rev.* **1994**, *94*, 2319-2358; b) C. Reichardt, T. Welton, *VCH* **2010**.

[5] J. Catalan, P. Perez, J. Laynez, F. G. Blanco, *J. Fluoresc.* **1991**, *1*, 215-223.

[6] a) G. Li, L. Tang, H. Liu, Y. Wang, G. Zhao, Z. Tang, *Org. Lett.* **2016**, *18*, 4526-4529; b) L. Guo, Z. Chen, H. Zhu, M. Li, Y. Gu, *Chin. Chem. Lett.* **2021**, *32*, 1419-1422.

[7] J. Mao, Z. Wang, X. Xu, G. Liu, R. Jiang, H. Guan, Z. Zheng, P. J. Walsh, *Angew. Chem. Int. Ed.* **2019**, *58*, 11033-11038.

[8] A. M. Prior, X. Yu, E.-J. Park, T. P. Kondratyuk, Y. Lin, J. M. Pezzuto, D. Sun, *Bioorg. Med. Chem. Lett.* **2017**, *27*, 5393-5399.

[9] S.-D. Yang, C.-L. Sun, Z. Fang, B.-J. Li, Y.-Z. Li, Z.-J. Shi, *Angew. Chem. Int. Ed.* **2008**, *47*, 1473-1476.

[10] H. Sakai, K. Tsutsumi, T. Morimoto, K. Kakiuchi, *Adv. Synth. Catal.* **2008**, *350*, 2498-2502.

[11] X.-H. Shan, H.-X. Zheng, B. Yang, L. Tie, J.-L. Fu, J.-P. Qu, Y.-B. Kang, *Nat. Commun.* **2019**, *10*, 908.

[12] T. Liu, K. Wu, L. Wang, Z. Yu, *Adv. Synth. Catal.* **2019**, *361*, 3958-3964.

[13] a) R. Rohlmann, T. Stopka, H. Richter, O. García Mancheño, *J. Org. Chem.* **2013**, *78*, 6050-6064; b) H. Tian, W. Xu, Y. Liu, Q. Wang, *Org. Lett.* **2020**, *22*, 5005-5008.

[14] R. Romagnoli, F. Prencipe, P. Oliva, M. Kimatrai Salvador, A. Brancale, S. Ferla, E. Hamel, G. Viola, R. Bortolozzi, L. Persoons, J. Balzarini, S. Liekens, D. Schols, *Bioorg. Chem.* **2020**, *97*, 103665.

[15] F. Zhao, Q. Yang, J. Zhang, W. Shi, H. Hu, F. Liang, W. Wei, S. Zhou, *Org. Lett.* **2018**, *20*, 7753-7757.
